# Antimicrobial Indole-3-Carboxamido-Polyamine Conjugates Target Bacterial Membranes and Are Antibiotic Potentiators

**DOI:** 10.3390/biom14030261

**Published:** 2024-02-22

**Authors:** Kenneth Sue, Melissa M. Cadelis, Florent Rouvier, Marie-Lise Bourguet-Kondracki, Jean Michel Brunel, Brent R. Copp

**Affiliations:** 1 School of Chemical Sciences, The University of Auckland, Private Bag 92019, Auckland 1142, New Zealand; 2 School of Medical Sciences, The University of Auckland, Private Bag 92019, Auckland 1142, New Zealand; 3 Membranes et Cibles Thérapeutiques, SSA, INSERM, Aix-Marseille Universite, 27 Bd Jean Moulin, 13385 Marseille, France; 4 Laboratoire Molécules de Communication et Adaptation des Micro-Organismes, UMR 7245 CNRS, Muséum National d’Histoire Naturelle, 57 Rue Cuvier (C.P. 54), 75005 Paris, France

**Keywords:** indole, potentiator, antimicrobial, polyamine, antibiotics, antifungal agents, structure–activity relationships

## Abstract

Small molecules that can restore the action of legacy antibiotics toward drug-resistant bacteria represent an area of ongoing research interest. We have previously reported indole-3-glyoxylamido and indole-3-acetamido-polyamine conjugates that exhibit intrinsic activity toward bacterial and fungal species, and the ability to enhance the action of doxycycline toward the Gram-negative bacteria *Pseudomonas aeruginosa*; however, these desirable activities were commonly associated with unfavorable cytotoxicity and/or red blood cell hemolytic properties. In this paper, we report the synthesis and biological investigation of a new class of α,ω-di(indole-3-carboxamido)polyamine derivatives, leading to the identification of several analogues that exhibit antimicrobial- and antibiotic-potentiating activities without detectable cytotoxic or hemolytic properties. 5-Bromo-substituted indole analogues **3** and **12**–**18** were generally more broad-spectrum in their activity than others in the set, with **13b** (polyamine PA-3-6-3) being particularly notable for its anti-*Staphylococcus aureus*, *Acinetobacter baumannii*, and *Cryptococcus neoformans* activities (MIC ≤ 0.28 µM). The same analogue also restored the action of doxycycline toward *P. aeruginosa* with a 21-fold enhancement, while the corresponding 5-bromo-indole-3-carboxamide-PA3-7-3 analogue was able to enhance the action of both doxycycline and erythromycin toward *P. aeruginosa* and *Escherichia coli*, respectively. The analogue **13b** was capable of disrupting the bacterial membrane of both *S. aureus* and methicillin-resistant *S. aureus* (MRSA) and the outer membrane of *P. aeruginosa*, suggesting that membrane perturbation could be a mechanism of action of both intrinsic antimicrobial activities and antibiotic potentiation.

## 1. Introduction

With the continuing rise in the incidence of infections caused by drug-resistant bacteria, there is an urgent need to identify and develop new antibiotics. The discovery of novel antibiotics, however, is a challenging process [1,2], with interest in natural products, the previous source of many of the frontline antibiotics, on the decline [3] and a lack of novel hit/lead discoveries [4,5] leading to a depleted development pipeline. There is an added complexity regarding the discovery of treatments of Gram-negative bacterial infections, with the microorganisms being inherently more resistant to antibiotics due to the low permeability of the outer membrane and the presence of transmembrane-spanning efflux pumps [6]. Rather than discovering new classes of antibiotics, an alternative treatment approach is the use of antibiotic adjuvants, also known as enhancers or potentiators, small molecules that can be used in combination with legacy antibiotics to restore their activity [7,8,9,10].

**Figure 1:**
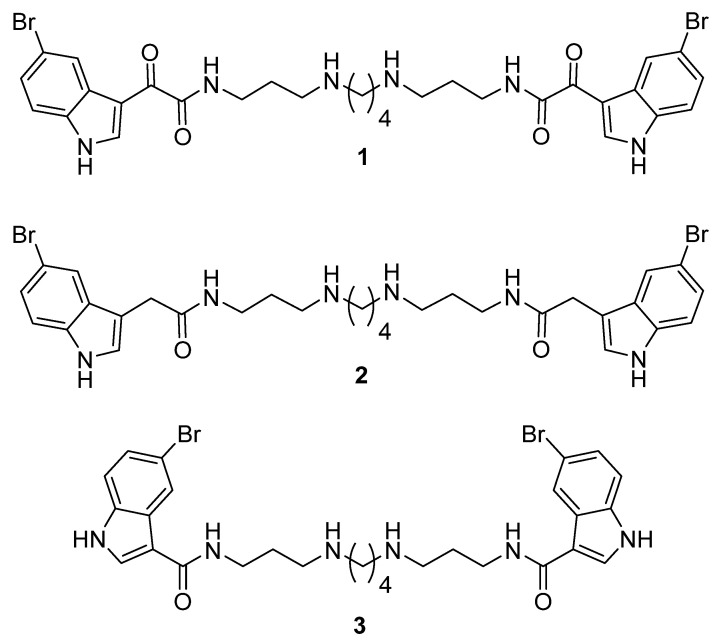
Structures of 5-bromo-substituted indole-3-glyoxylamido (**1**), indole-3-acetamido (**2**), and indole-3-carboxamido (**3**) polyamine conjugates.

Our interest in antibiotic adjuvants started with the discovery that 5-bromoindole-3-glyoxamido spermine **1** (Figure 1) exhibited intrinsic antimicrobial activities toward Gram-positive bacteria and fungi, enhanced the action of doxycycline toward the Gram-negative bacteria *Pseudomonas aeruginosa*, and could also disrupt the integrity of bacterial membranes [11]. The compound class unfortunately had associated cytotoxic and/or red blood cell hemolytic properties, which prompted the exploration of an analogous set of indole-3-acetamido-polyamine conjugates (e.g., **2**) that retained intrinsic antimicrobial- and antibiotic-enhancing properties [12]. While **2** itself was devoid of cytotoxicity and hemolytic properties, many examples in the set of indole-3-acetamido polyamines still exhibited hemolytic properties. Prior to the current study, a single example of a structurally simpler α,ω-di-(5-bromoindole-3-carboxamido)spermine (**3**) was prepared and found to exhibit intrinsic antimicrobial activity toward *Staphylococcus aureus* and methicillin-resistant *S. aureus* (MRSA) but not Gram-negative bacteria and to enhance the action of doxycycline, chloramphenicol, and nalidixic acid against *P. aeruginosa* with little to no cytotoxic/hemolytic properties [13] (Figure 1).

Based upon the favorable biological properties exhibited by polyamine derivative **3**, we prepared an extended set of analogues, exploring the variation in substitution on the indole-3-carboxamide group as well as variation in the length of the polyamine chain. The analogues were evaluated against a panel of bacteria (Gram-positive and Gram-negative) and two fungi for cytotoxicity and red blood cell hemolytic properties and for the ability to enhance the action of doxycycline against *P. aeruginosa* and erythromycin against *Escherichia coli*.

## 2. Materials and Methods

### 2.1. Chemistry Synthesis General Methods

Infrared spectra were recorded on a Perkin-Elmer spectrometer 100 Fourier-transform infrared spectrometer (Waltham, MA, USA) equipped with a universal ATR accessory. Mass spectra were acquired on a Bruker micrOTOF Q II spectrometer (Bremen, Germany). ^1^H and ^13^C NMR spectra were recorded at 298 K on a Bruker Avance III HD 400 spectrometer (Karlsruhe, Germany) using standard pulse sequences. Proto-deutero solvent signals were used as internal references (CD_3_OD: δ_H_ 3.31, δ_C_ 49.00). For ^1^H NMR, the data are quoted as position (δ), relative integral, multiplicity (s = singlet, d = doublet, t = triplet, q = quartet, dd = doublet of doublets, td = triplet of doublets, tt = triplet of triplets, m = multiplet, and br = broad), coupling constant (*J*, Hz), and assignment to the atom. The ^13^C NMR data are quoted as position (δ), and assignment to the atom. Flash column chromatography was carried out using Davisil silica gel (40–60 μm) or Merck LiChroprep RP-8 (40–63 µm) (Darmstadt, Germany). Thin layer chromatography was conducted on Merck DC Kieselgel 60 RP-18 F254S (Darmstadt, Germany) plates. All solvents used were of analytical grade or better and/or purified according to standard procedures. The chemical reagents used were purchased from standard chemical suppliers and used as purchased. All indole-3-carboxylic acids were commercially available. The protected polyamines di-*tert*-butyl butane-1,4-diylbis((3-aminopropyl)carbamate) (**11a**), di-*tert*-butyl hexane-1,6-diylbis((3-aminopropyl)carbamate) (**11b**), di-*tert*-butyl heptane-1,7-diylbis((3-aminopropyl)carbamate) (**11c**), di-*tert*-butyl octane-1,8-diylbis((3-aminopropyl)carbamate) (**11d**), di-*tert*-butyl decane-1,10-diylbis((3-aminopropyl)carbamate) (**11e**) and di-*tert*-butyl dodecane-1,12-diylbis((3-aminopropyl)carbamate) (**11f**) [11,12,13], and target polyamine-conjugate **3** [13] were synthesized according to the literature procedures.

#### 2.1.1. General Procedure A: Amide Bond Formation of Indole Analogues

A solution of the corresponding indole-3-carboxylic acid (2.2 equiv.), EDC·HCl (2.6 equiv.), HOBt (2.6 equiv.), and DIPEA (6 equiv.) was stirred in dry CH_2_Cl_2_ (1 mL) at 0 °C for 10 min under N_2_. To the reaction mixture was added the appropriate Boc-protected polyamine (1 equiv.) in dry CH_2_Cl_2_ (1 mL) and the solution was stirred overnight. To the reaction mixture was then added CH_2_Cl_2_ (20 mL), and the organic solvent phase was washed with saturated aq. NaHCO_3_ (30 mL) followed by H_2_O (2 × 30 mL), then dried under reduced pressure, and purified by silica gel column chromatography (4–8% MeOH/CH_2_Cl_2_) to obtain the desired product.

#### 2.1.2. General Procedure B—Boc Deprotection

A solution of *tert*-butyl-carbamate derivative in CH_2_Cl_2_ (2 mL) and TFA (0.2 mL) was stirred at room temperature under N_2_ for 2 h followed by solvent removal under reduced pressure. The crude product was purified by C_8_ reversed-phase flash column chromatography eluting with 0–50% MeOH/H_2_O (0.05% TFA) to obtain the corresponding polyamine as a di-TFA salt.

### 2.2. Synthesis of Compounds

See Supplementary File S1.

### 2.3. Antimicrobial Assays

The susceptibility of the bacterial strains *S. aureus* (ATCC 25923), *E. coli* (ATCC 25922), and *P. aeruginosa* (ATCC 27853) to antibiotics and compounds was determined in microplates using the standard broth dilution method in accordance with the recommendations of the Comité de l’AntibioGramme de la Société Française de Microbiologie (CA-SFM). Briefly, the minimal inhibitory concentrations (MICs) were determined with an inoculum of 10^5^ CFU in 200 µL of Mueller–Hinton broth (MHB) containing two-fold serial dilutions of each drug. The MIC was defined as the lowest concentration of the drug that completely inhibited visible growth after incubation for 18 h at 37 °C. To determine all MICs, the measurements were independently repeated in triplicate. Additional antimicrobial evaluations against MRSA (ATCC 43300), *Klebsiella pneumoniae* (ATCC 700603), *A. baumannii* (ATCC 19606), *Candida albicans* (ATCC 90028), and *Cryptococcus neoformans* (ATCC 208821) were undertaken at the Community for Open Antimicrobial Drug Discovery at The University of Queensland (Australia), according to their standard protocols as reported previously [14]. For antimicrobial assays, the tested strains were cultured in either Luria broth (LB) (In Vitro Technologies, USB75852, Melbourne, VIC, Australia), nutrient broth (NB) (Becton Dickson, 234000, Macquarie Park, NSW, Australia), or MHB at 37 °C overnight. A sample of the culture was then diluted 40-fold in fresh MHB and incubated at 37 °C for 1.5−2 h. The compounds were serially diluted 2-fold across the wells of 96-well plates (Corning 3641, non-binding surface, Gilbert, AZ, USA), with compound concentrations ranging from 0.015 to 64 μg/mL and plated in duplicate. The resultant mid-log phase cultures were diluted to the final concentration of 1 × 10^6^ CFU/mL; then, 50 μL was added to each well of the compound-containing plates, producing a final compound concentration ranging from 0.008 to 32 μg/mL and a cell density of 5 × 10^5^ CFU/mL. All plates were then covered and incubated at 37 °C for 18 h. Resazurin was added at a 0.001% final concentration to each well and incubated for 2 h before the MICs were read by eye.

For the antifungal assay, fungi strains were cultured for 3 days on YPD agar at 30 °C. A yeast suspension of 1 × 10^6^ to 5 × 10^6^ CFU/mL was prepared from five colonies. These stock suspensions were diluted with a yeast nitrogen base (YNB) (Becton Dickinson, 233520, New South Wales, Australia) broth to a final concentration of 2.5 × 10^3^ CFU/mL. The compounds were serially diluted 2-fold across the wells of 96-well plates (Corning 3641, non-binding surface), with compound concentrations ranging from 0.015 to 64 μg/mL and final volumes of 50 μL, plated in duplicate. Then, 50 μL of the fungi suspension that was previously prepared in the YNB broth to the final concentration of 2.5 × 10^3^ CFU/mL was added to each well of the compound-containing plates, producing a final compound concentration ranging from 0.008 to 32 μg/mL. The plates were covered and incubated at 35 °C for 36 h without shaking. *C. albicans* MICs were determined by measuring the absorbance at OD_530_. For *C. neoformans*, resazurin was added at a 0.006% final concentration to each well and incubated for a further 3 h before the MICs were determined by measuring the absorbance at OD_570–600_.

Colistin and vancomycin were used as positive bacterial inhibitor standards for Gram-negative and Gram-positive bacteria, respectively. Fluconazole was used as a positive fungal inhibitor standard for *C. albicans* and *C. neoformans*. The antibiotics were provided in 4 concentrations, with 3 above and 3 below their MIC values, and plated in the first 8 wells of column 23 of the 384-well NBS plates. The quality control (QC) of the assays was determined by the antimicrobial controls and the Z′-factor (using positive and negative controls). Each plate was deemed to fulfil the quality criteria (pass QC) if the Z′-factor was above 0.4, and the antimicrobial standards showed a full range of activity, with full growth inhibition at their highest concentration, and no growth inhibition at their lowest concentration.

### 2.4. Real-Time Growth Curves

The solutions of the compound at the concentrations of 2, 4, and 16 µg/mL were tested each in triplicate against *S. aureus* ATCC 25923. Typically, in a 96-well plate were placed 10 µL of 40, 80, and 320 µg/mL stock solutions of compound, as well as 190 µL of 5 × 10^5^ CFU/mL of the selected bacterial suspension in brain heart infusion (BHI) broth. Positive controls containing only 200 µL of 5 × 10^5^ CFU/mL of the bacterial suspension in BHI and negative controls containing only 200 µL of BHI broth were added. The plate was incubated at 37 °C in a TECAN Spark Reader (Roche Diagnostic, Meylan, France) and the real-time bacterial growth was followed by OD_590_ nm measurements every 20 min for 18 h.

### 2.5. Measurement of the ATP Efflux

A solution of the compound was prepared in twice-distilled water at a fixed concentration of 100 μg/mL. The different Gram-positive suspensions were prepared in MH II broth and were incubated at 37 °C. Then, 90 µL of each bacterial suspension was added to 10 µL of the compound solution and shaken for 20 s in the incubator at 37 °C. Subsequently, 50 µL of Luceferin–Luciferase reagent (Yelen, Marseille, France) was added to the mixture, and the luminescent signal was quantified with an Infinite M200 microplate reader (Tecan, Männedorf, Switzerland) for 6 readings spaced 30 s apart. The ATP concentration was quantified by internal sample addition. Squalamine (100 µg/mL) was used as the positive control to quantify the maximum level of ATP efflux and water as the negative control. This assay was performed in three independent experiments.

### 2.6. Determination of the MICs of Antibiotics in the Presence of Synergizing Compounds

Briefly, restoring enhancer concentrations were determined with an inoculum of 5 × 10^5^ CFU in 200 µL of MHB containing two-fold serial dilutions of each derivative in the presence of doxycycline at 4.5 μM (2 µg/mL) and erythromycin 2.7 μM (2 µg/mL). The lowest concentration of the polyamine adjuvant that completely inhibited visible growth after incubation for 18 h at 37 °C was determined. These measurements were independently repeated in triplicate.

### 2.7. Nitrocefin Hydrolysis Assay

Outer membrane permeabilization was measured using nitrocefin as a chromogenic substrate of periplasmic β-lactamase. A total of 10 milliliters of MH broth were inoculated with 0.1 mL of an overnight culture of PAO1 bacteria and grown at 37 °C until the OD_600_ reached 0.5. The remaining steps were performed at room temperature. The cells were recovered by centrifugation (4000 rpm for 20 min) and washed once in 20 mM potassium phosphate buffer (pH 7.2) containing MgCl_2_ (1 mM). After a second centrifugation, the pellet was resuspended and adjusted to OD_600_ of 0.5. Then, 50 µL of the desired compound was added to 100 µL of the cell suspension to obtain a final concentration varying from 3.9 µM to 250 µM. A total of 50 microliters of nitrocefin was then added to obtain a final concentration of 50 µg/mL. Nitrocefin hydrolysis was monitored spectrophotometrically by measuring the increase in absorbance at 490 nm. The assays were performed in 96-well plates using a M200 Pro Tecan (Tecan, Männedorf, Switzerland) spectrophotometer.

### 2.8. Cytotoxicity Assays

HEK293 cells were counted manually in a Neubauer hemocytometer and plated at a density of 5000 cells/well into each well of the 384-well plates containing the 25× (2 μL) concentrated compounds [14]. The medium used was Dulbecco’s modified Eagle’s medium (DMEM), supplemented with 10% fetal bovine serum (FBS). The cells were incubated together with the compounds for 20 h at 37 °C, 5% CO_2_. To measure cytotoxicity, 5 μL (equals 100 μM final) of resazurin was added to each well after incubation and incubated for a further 3 h at 37 °C with 5% CO_2_. After the final incubation, the fluorescence intensity was measured as Fex 560/10 nm, em 590/10 nm (F_560/590_), using a Tecan M1000 Pro (Tecan, Männedorf, Switzerland) monochromator plate reader. The CC_50_ values (concentration at 50% cytotoxicity) were calculated by normalizing the fluorescence readout, with 74 μg/mL tamoxifen as the negative control (0%) and normal cell growth as the positive control (100%). The concentration-dependent percentage cytotoxicity was fitted to a dose–response function (using Pipeline Pilot) and the CC_50_ values were determined.

### 2.9. Hemolytic Assay

Human whole blood was washed three times with three volumes of 0.9% NaCl and then resuspended in the same solution to a concentration of 0.5 × 10^8^ cells/mL, as determined by the manual cell count in a Neubauer hemocytometer [14]. The washed cells were then added to the 384-well compound-containing plates for a final volume of 50 μL. After a 10 min shake on a plate shaker, the plates were then incubated for 1 h at 37 °C. After incubation, the plates were centrifuged at 1000× *g* for 10 min to pellet cells and debris; 25 μL of the supernatant was then transferred to a polystyrene 384-well assay plate. Hemolysis was determined by measuring the supernatant absorbance at 405 mm (OD_405_). The absorbance was measured using a Tecan M1000 Pro monochromator plate reader. HC_10_ and HC_50_ (concentrations at 10% and 50% hemolysis, respectively) were calculated by a curve fitting the inhibition values vs. log (concentration) using a sigmoidal dose–response function with variable fitting values for the top, bottom, and slope.

## 3. Results and Discussion

### Chemistry

**Figure 2:**
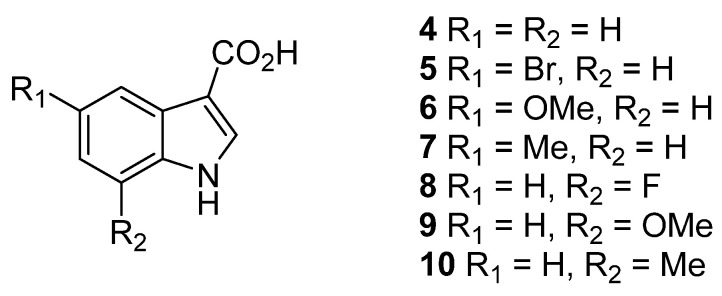
Substituted indole-3-carboxylic acids **4**–**10**.

**Figure 3:**
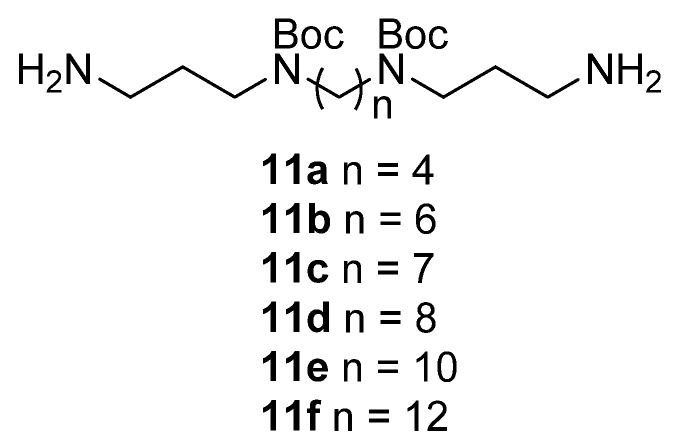
Boc-protected polyamines **11a**–**f**.

**Scheme 1:**
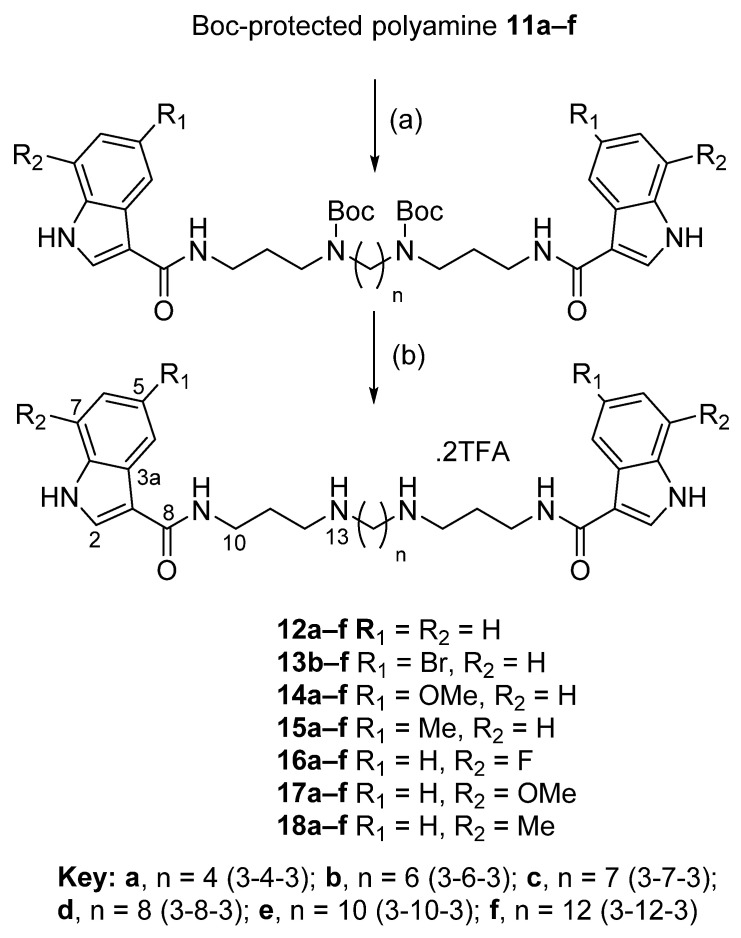
General method for the synthesis of the target polyamine analogues **3** and **12**–**18**. Reagents and conditions: (**a**) Carboxylic acid (**4**–**10**) (2.2 equiv.), Boc-protected polyamine (**11a**–**f**) (1 equiv.), EDC·HCl (2.6 equiv.), HOBt (2.6 equiv.), DIPEA (6 equiv.), CH_2_Cl_2_, rt, overnight (yields of 9–96%); (**b**) TFA (0.2 mL), CH_2_Cl_2_ (2 mL), N_2_, rt, 2 h (yields of 13–100%).

The target-substituted indole-3-carboxamide polyamines were prepared through the amide coupling of substituted indole-3-carboxylic acids **4**–**10** (Figure 2) to Boc-protected polyamines **11a**–**f** (Figure 3) mediated by EDC·HCl and HOBt (Scheme 1). The crude reaction products were subjected to purification by silica gel column chromatography (4–8% MeOH/CH_2_Cl_2_), obtaining the desired Boc-protected indole-3-carboxamides in highly variable yields (9–96%). The Boc-protected indole-3-carboxamides were then subjected to deprotection using 10:1 CH_2_Cl_2_/TFA and purified by RP C_8_ column chromatography (50–75% MeOH/H_2_O (0.05% TFA)) to obtain the desired indole-3-carboxamide polyamines **3** and **12**–**18** as di-TFA salts in a 13–100% yield.

All of the indole-3-carboxamide-polyamine conjugates **3** and **12**–**18** were evaluated for antimicrobial activity toward a panel of organisms comprising Gram-positive bacteria (*S. aureus* ATCC 25923 (Sa) and MRSA ATCC 43300), Gram-negative bacteria (*E. coli* ATCC 28922 (Ec), *P. aeruginosa* ATCC 27853 (Pa), *K. pneumoniae* ATCC 700603 (Kp), and *A. baumannii* ATCC 19606 (Ab)), and fungi (*C. albicans* ATCC 90028 (Ca) and *C. neoformans* ATCC 208821 (Cn)) (Table 1). The results are presented as the minimum inhibitory concentrations (MIC) with units in micromolarity. Overall, the library of analogues typically exhibited a pronounced growth inhibition of Gram-positive bacteria (Sa and MRSA) and the fungus *C. neoformans*, but only a limited subset of compounds exhibited activity toward the second fungal test strain (*C. albicans*) or Gram-negative bacteria. In the current sample set, the 5-bromo-substitited indole analogues **3** and **13b**–**f** were generally more broad-spectrum in their activity than the other functional-group substituted analogues, with the PA3-6-3 (**13b**) and PA3-12-3 (**13f**) analogues being particularly noteworthy. Of the other substituents on the indole ring, 5-methyl variants were consistently active against Sa and Cn irrespective of the polyamine chain length, while for most other substituents, or H, activity was associated with the polyamines of lengths greater than PA3-4-3 or PA3-6-3. In nearly all cases, there was a higher likelihood of Gram-negative and anti-*C. albicans* activity associated with the longer PA3-12-3 polyamine chain lengths. We observed similar activity profiles previously for α,ω-disubstituted polyamines containing indole-3-acetic acid [12] and indole-3-glyoxylic acid [11] capping groups.

**Figure 4:**
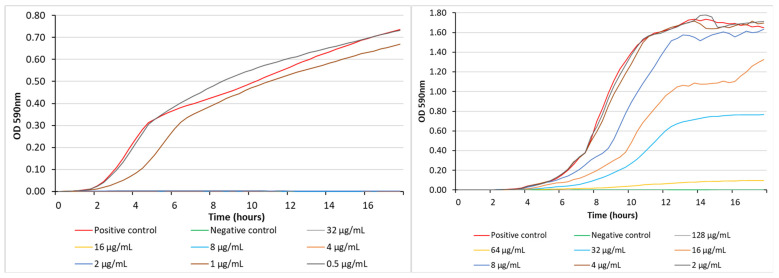
Bacterial growth inhibition exhibited by **13b** against *S. aureus* ATCC 25923 (**left**) and *P. aeruginosa* PAO1 (**right**) at different concentrations. The positive control was bacteria-only and the negative control was media-only.

The real-time growth inhibition profiles of **13b** against both *S. aureus* ATCC 25923 and *P. aeruginosa* PAO1 were then determined to evaluate the dynamics of its antibacterial activity. In the case of *S. aureus*, complete inhibition was achieved at concentrations ranging from 32 to 2 μg/mL (35.5 to 2.2 μM), with bacterial growth resuming after 2 h at a test concentration of 1 μg/mL (1.1 μM). It is interesting to note the step-change in growth characteristics of the organism in the presence of MIC dose (1 µg/mL) and 0.5×MIC (0.5 µg/mL). For the Gram-negative bacteria *P. aeruginosa*, growth inhibition was only observed at the higher concentrations of 128 and 64 μg/mL (142 and 70.9 μM) with bacterial growth resuming after 4 h at 32 μg/mL (35.5 μM) (Figure 4). The growth response of *P. aeruginosa* toward the test compound was more nuanced, with some growth inhibition observed at test concentrations down to 0.25×MIC. The traditional microdilution methodology yielded MIC values for **13b** of 2 μg/mL (2.2 μM) for *S. aureus* and 64 μg/mL (71 μM) for *P. aeruginosa*, both of which agreed with the data observed at the 18 h time point in the real-time growth inhibition curves.

**Figure 5:**
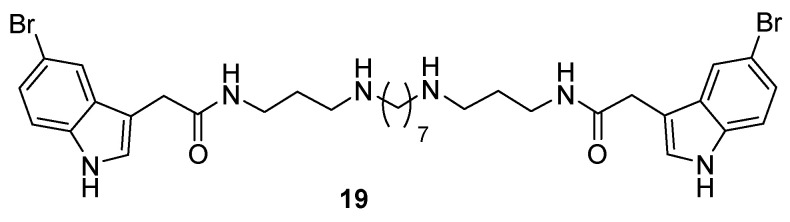
Structure of the 5-bromo-indole-3-acetamido-polyamine 3-7-3 conjugate **19**.

**Figure 6:**
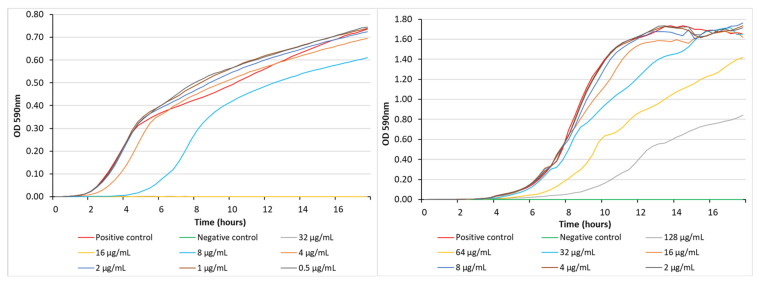
Bacterial growth inhibition exhibited by **19** against *S. aureus* ATCC 25923 (**left**) and *P. aeruginosa* PAO1 (**right**) at different concentrations. The positive control was bacteria-only and the negative control was media-only.

In a recent study, we described a series of α,ω-disubstituted indole-3-acetamido polyamine conjugates [12]. Among these compounds, a representative biologically active example was the 5-bromoindole 3-7-3 analogue, **19** (Figure 5), which demonstrated moderate activity against *S. aureus* ATCC 25923 (MIC 12.5 μg/mL, 12.7 μM) but was essentially inactive against *P. aeruginosa* ATCC 27853 (MIC 100 μg/mL, 106 μM) [12]. To compare **19** and **13b** from the current study, we also examined the real-time growth inhibition profiles of **19** against *S. aureus* ATCC 25923 and *P. aeruginosa* PAO1 (Figure 6). In keeping with the MIC values determined using microbroth dilution methods, the complete inhibition of *S. aureus* was achieved at concentrations of 32 and 16 μg/mL (33.9 and 16.9 μM, respectively), with bacterial growth resuming after 2 h at a concentration of 8 μg/mL (8.5 μM), while for *P. aeruginosa*, organism growth was still observed at the highest test concentration of 128 μg/mL (135.5 μM), with bacterial growth resuming after 4 h. Thus, the direct comparison of compounds **13b** and **19** identified the former as being more active against both bacterial strains.

**Figure 7:**
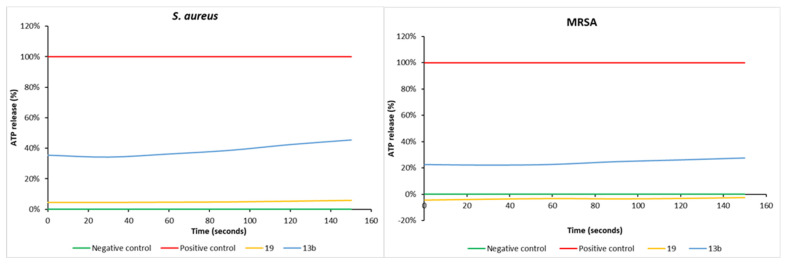
ATP release in *S. aureus* ATCC 25923 (**left**) and MRSA (**right**) exhibited by **13b** and **19**. Squalamine (100 µg/mL) was the positive control and water was the negative control. The compounds were tested at a fixed concentration of 100 µg/mL, and the results are reported as a percentage (%) relative to the positive control.

Substituted polyamines have previously demonstrated the ability to disrupt/permeabilize bacterial outer membranes [13,15,16,17,18]. Using a biochemical assay to detect the induced leakage of intracellular ATP, the single-dose (100 µg/mL) testing of **13b** and **19** identified the former to be the more potent of the two compounds in its ability to disrupt the bacterial outer membrane of both *S. aureus* ATCC 25923 and MRSA (Figure 7) in keeping with its stronger growth inhibition properties against the same organism (**13b** MIC of 2.2 µM; **19** MIC of 12.7 µM). Both compounds were noticeably less active than the positive control squalamine [19,20].

Next, the indole-3-carboxamide-polyamine conjugates **12**–**18** were assessed for their capacity to augment the effectiveness of doxycycline against *P. aeruginosa* ATCC 27853 and of erythromycin against *E. coli* ATCC 25922 (Table 2) at a fixed sub-MIC dosage of 2 µg/mL of doxycycline (4.5 µM, MIC of 90 µM) or erythromycin (2.7 µM, MIC of 174 µM). For the doxycycline–*P. aeruginosa* combination, significant improvements in effectiveness were observed for three analogues. The more active of these three, the 5-bromo 3-6-3 analogue **13b**, displayed an excellent 21-fold increase in activity, while the 5-bromo and 7-methyl PA3-7-3 analogues (**13c** and **18c**) both exhibited a moderate 16-fold enhancement in activity. By comparison, the previously reported 5-bromo 3-4-3 analogue, **3**, exhibited a 64-fold enhancement in activity [13]. However, unlike **3**, which had no impact on the efficacy of erythromycin against *E. coli*, one of the new analogues, **13c**, demonstrated a moderate 16-fold enhancement in activity.

**Figure 8:**
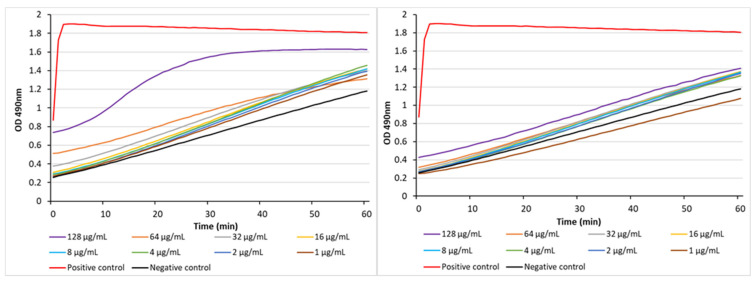
The abilities of **13b** (**left**) and **19** (**right**) to act as membrane disruptors in *P. aeruginosa* PAO1 as determined using a nitrocefin hydrolysis assay. Polymyxin B (PMB) was the positive control (128 µg/mL) and the negative control was bacteria with nitrocefin.

As noted in Table 2, indole-3-carboxamide **13b** can enhance the action of doxycycline toward the Gram-negative bacteria *P. aeruginosa* with an MIC of 3.46 µM, representing a 21-fold enhancement of activity. By comparison, we previously reported that indole-3-acetamide **19** was also active in the same assay, demonstrating a combined MIC of 6.6 µM, which corresponds to an 18-fold enhancement [12]. A potential mechanism by which these compounds could enhance the antibiotic action of doxycycline toward *P. aeruginosa* is to disrupt the bacterial outer membrane, facilitating the entry of the antibiotic. The compounds were assessed using a nitrocefin colorimetric assay, whereby membrane disruption leads to the entry of the chromogenic cephalosporin substrate with the subsequent β-lactam hydrolysis by periplasmic β-lactamases leading to a detectable color change from yellow to red. Carboxamide **13b** was able to disrupt the outer membrane only at the top dose (128 µg/mL), while indole-3-acetamide **19** was inactive at all test doses (Figure 8). We conclude from this that either Gram-negative bacterial outer membrane disruption is not a universal mechanism of antibiotic potentiation of α,ω-disubstituted polyamines or that the two test compounds enhance the action of doxycycline toward *P. aeruginosa* via different mechanisms.

Finally, the cytotoxic and hemolytic properties of the compound library were assessed, and the results are summarized in Table 3. Cytotoxicity was determined against the HEK293 cell line and is reported as the concentration of the compound causing 50% cytotoxicity (IC_50_), while the hemolytic properties were determined against human red blood cells and the results are presented as the concentration of the compound required to induce 10% hemolytic activity (HC_10_). Cytotoxic effects were only observed in the case of two analogues, **13f** (IC_50_ ≤ 0.25 μM) and **15f** (IC_50_ 0.55 μM), both of which were PA3-12-3 analogues, with **13f** also being the sole analogue to exhibit hemolytic properties (HC_10_ 14.2 μM). This set of indole-3-carboxamide polyamines was considerably less cytotoxic or hemolytic than the corresponding series of analogues bearing indole-3-acetic [12] or indole-3-glyoxylic [11] capping acids, identifying it as worthy of further investigation and optimization.

## 4. Conclusions

Our search to optimize the intrinsic antimicrobial- and antibiotic-enhancing properties of indole-3-glyoxylamido polyamines led to our current investigation of indole-3-carboxamides as capping acids. Compared to the compounds described in our first report, the results presented in this paper identify polyamines bearing the structurally simpler capping acid as being promising non-cytotoxic and non-hemolytic broad-spectrum antimicrobials that can also increase the activity of legacy antibiotics, possibly by a mechanism attributable to the alteration of the permeability of the bacterial outer membrane.

## Figures and Tables

**Table 1:** Antimicrobial activities (MIC, µM) of analogues **3** and **12**–**18**.

Compound	MIC (µM)							
	* Sa* ^a^	* MRSA* ^b^	* Pa* ^c^	* Ec* ^d^	* Kp* ^e^	*Ab* ^f^	*Ca* ^g^	*Cn* ^h^
**12a**	69.8	22.3	>279 ^i^	139	>45 ^j^	>45	>45	n.t ^k^
**12b**	268	≤0.34	>268	134	>43	>43	>43	≤0.34
**12c**	132	≤0.33	>263	65.9	>42	>42	>42	≤0.33
**12d**	64.7	≤0.32	>259	32.4	>41	>41	>41	≤0.32
**12e**	31.2	≤0.31	>250	125	>40	>40	>40	≤0.31
**12f**	3.77	≤0.30	121	15.1	>39	4.82	≤0.30	≤0.30
**3**	4.8 ^i^	12.4 ^i^	200 ^i^	200 ^i^	>50 ^i^	>50 ^i^	49.5 ^i^	12.4 ^i^
**13b**	2.2	≤0.28	71	6.92	>35	≤0.28	>35	≤0.28
**13c**	13.6	≤0.27	220	110	>35	>35	35	≤0.27
**13d**	3.36	≤0.27	107	53.7	>34	≤0.27	≤0.27	≤0.27
**13e**	3.26	≤0.26	104	52.2	>34	16.7	4.17	≤0.26
**13f**	3.17	≤0.25	203	>203	≤0.25	2.03	≤0.25	≤0.25
**14a**	257	10.3	>257	257	>41	>41	>41	n.t ^k^
**14b**	248	≤0.31	>248	248	>40	>40	>40	≤0.31
**14c**	122	>39	>244	>244	>39	>39	>39	n.t ^k^
**14d**	60.0	19.2	>240	120	>38	>38	>38	≤0.30
**14e**	29.0	≤0.29	232	58.1	>37	>37	>37	≤0.29
**14f**	3.52	≤0.28	112	14.1	>36	>36	≤0.28	≤0.28
**15a**	67.1	≤0.34	>269	>269	>43	>43	>43	≤0.34
**15b**	259	5.18	>259	129	>41	>41	>41	≤0.32
**15c**	63.5	≤0.32	>254	254	>41	>41	>41	≤0.32
**15d**	31.2	≤0.31	250	125	>40	>40	>40	≤0.31
**15e**	3.77	≤0.30	60.3	30.2	>39	19.3	≤0.30	≤0.30
**15f**	3.65	≤0.29	58.3	29.2	>37.3	≤0.29	≤0.29	≤0.29
**16a**	>266	>43	266	>266	>43	>43	>43	n.t ^k^
**16b**	256	>41	>256	256	>41	>41	>41	n.t ^k^
**16c**	>251	≤0.31	251	>251	>40	>40	>40	≤0.31
**16d**	247	9.89	247	247	>40	>40	>40	≤0.31
**16e**	239	≤0.30	239	239	>38	>38	≤0.30	≤0.30
**16f**	231	≤0.29	116	231	>37	>37	>37	≤0.29
**17a**	129	20.6	>257	>257	>41	>41	>41	n.t ^k^
**17b**	124	>40	>248	248	>40	>40	>40	n.t ^k^
**17c**	122	2.44	>244	244	>39	>39	>39	≤0.31
**17d**	120	≤0.30	>240	240	>38	>38	>38	≤0.30
**17e**	232	≤0.29	>232	>232	>37	>37	>37	≤0.29
**17f**	7.03	≤0.28	225	28.1	>36	>36	4.50	≤0.28
**18a**	>269	21.5	269	>269	>43	>43	>43	n.t ^k^
**18b**	129	>41	>259	129	>41	>41	>41	≤0.32
**18c**	>254	≤0.32	254	>254	>41	>41	>41	≤0.32
**18d**	>250	≤0.31	250	>250	>40	>40	>40	≤0.31
**18e**	30.2	≤0.30	60.3	30.2	>39	>39	4.83	≤0.30
**18f**	117	≤0.29	117	117	>37	9.33	≤0.29	≤0.29
Streptomycin	21.5		21.5	21.5				
Chloramphenicol	1.5–3							
Vancomycin		0.7						
Colistin			1	2	0.2	0.2		
Fluconazole							0.4	26

MIC data presented as an average of two or three independent experiments; ^a^ *S. aureus* ATCC 25923; ^b^ MRSA ATCC 43300; ^c^ *P. aeruginosa* ATCC 27853; ^d^ *E. coli* ATCC 25922; ^e^ *K. pneumoniae* ATCC 700603; ^f^ *A. baumannii* ATCC 19606; ^g^ *C. albicans* ATCC 90028; ^h^ *C. neoformans* ATCC 208821; ^i^ Data taken from Cadelis et al. [13]; ^j^ Not active at a single dose of 32 µg/mL; ^k^ Not tested.

**Table 2:** Doxycycline potentiation activity (MIC, µM) of analogues **3** and **12**–**18**.

Compound	Dox/Pa ^a^	Erythro/Ec ^b^	Compound	Dox/Pa ^a^	Erythro/Ec ^b^
**12a**	34.9 (>8)	34.9 (4)	**15d**	31.2 (8)	31.2 (4)
**12b**	33.6 (>8)	33.6 (4)	**15e**	15.1 (4)	7.54 (4)
**12c**	65.9 (>4)	65.9 (1)	**15f**	117 (0.5)	29.2 (1)
**12d**	32.3 (>8)	n.t ^c^	**16a**	266 (1)	>266 (1)
**12e**	62.4 (>4)	62.4 (2)	**16b**	64.0 (>4)	128 (2)
**12f**	15.1 (8)	7.54 (2)	**16c**	62.9 (>4)	252 (1)
**3**	3.125 ^c^ (64)	100 (2)	**16d**	30.9 (>8)	247 (1)
**13b**	3.46 (21)	6.92 (1)	**16e**	29.9 (8)	239 (1)
**13c**	13.6 (16)	6.82 (16)	**16f**	116 (1)	231 (1)
**13d**	13.4 (8)	6.72 (8)	**17a**	129 (>2)	>257 (1)
**13e**	26.1 (4)	6.52 (8)	**17b**	>248 (1)	>248 (1)
**13f**	50.7 (4)	25.3 (>8)	**17c**	30.5 (>8)	244 (1)
**14a**	64.4 (>4)	257 (1)	**17d**	60.0 (>4)	120 (2)
**14b**	31.1 (>8)	62.1 (4)	**17e**	58.1 (>4)	>232 (1)
**14c**	61.1 (>4)	244 (>1)	**17f**	56.2 (4)	56.2 (0.5)
**14d**	30.0 (>8)	240 (0.5)	**18a**	269 (1)	>269 (1)
**14e**	29.0 (8)	116 (0.5)	**18b**	32.4 (>8)	32.4 (4)
**14f**	56.2 (2)	14.1 (1)	**18c**	15.9 (16)	>254 (1)
**15a**	33.6 (>8)	67.1 (>4)	**18d**	31.2 (8)	250 (1)
**15b**	32.4 (>8)	32.4 (4)	**18e**	15.1 (4)	15.1(2)
**15c**	63.5 (>4)	63.5 (4)	**18f**	29.2 (4)	116.7 (1)

^a^ Concentration (µM) required to restore doxycycline activity at 2 µg/mL (4.5 µM) against *P. aeruginosa* ATCC 27853. The MIC of doxycycline alone is 90 µM. Fold-change shown in parentheses is the ratio between the intrinsic MIC of the test compound and the combination MIC. Values presented as the mean (n = 3); ^b^ Concentration (µM) required to restore erythromycin activity at 2 µg/mL (2.7 µM) against *E. coli* ATCC 28922. The MIC of erythromycin alone is 174 µM. Fold-change shown in parentheses is the ratio between the intrinsic MIC of the test compound and the combination MIC. Values presented as the mean (n = 3); ^c^ Data taken from Cadelis et al. [13].

**Table 3:** Cytotoxicity (IC_50_, µM) and hemolytic activity (HC_10_, µM) of analogues **3** and **12**–**18**.

Compound	Cytotoxicity ^a^	Hemolysis ^b^	Compound	Cytotoxicity ^a^	Hemolysis ^b^
**12a**	>45	>45	**15d**	>40	>40
**12b**	>43	>43	**15e**	>39	>39
**12c**	>42	>42	**15f**	0.55	>37
**12d**	>41	>41	**16a**	>43	>43
**12e**	>40	>40	**16b**	>41	>41
**12f**	>39	>39	**16c**	>40	>40
**3**	>50 ^c^	>50 ^c^	**16d**	>40	>40
**13b**	>35	>35	**16e**	>38	>38
**13c**	>35	>35	**16f**	>37	>37
**13d**	>34	>34	**17a**	>41	>41
**13e**	>33	>33	**17b**	>40	>40
**13f**	≤0.25	14.2	**17c**	>39	>39
**14a**	>41	>41	**17d**	>38	>38
**14b**	>40	>40	**17e**	>37	>37
**14c**	>39	>39	**17f**	>36	>36
**14d**	>38	>38	**18a**	>43	>43
**14e**	>37	>37	**18b**	>41	>41
**14f**	>36	>36	**18c**	>41	>41
**15a**	>43	>43	**18d**	>40	>40
**15b**	>41	>41	**18e**	>39	>39
**15c**	>41	>41	**18f**	>37	>37

All values presented as the mean (n = 2); ^a^ Concentration of compound at 50% cytotoxicity on HEK293 human embryonic kidney cells with tamoxifen as the positive control (IC_50_ 24 μM); ^b^ Concentration of the compound at 10% hemolytic activity on human red blood cells with melittin as the positive control (HC_10_ 0.95 μM); ^c^ Data taken from Cadelis et al. [13].

## Data Availability

Data are contained within the article or Supplementary Materials.

## Supplementary Materials

The following supporting information can be downloaded at: https://www.mdpi.com/article/10.3390/biom14030261/s1. Figure S1: ^1^H (CD_3_OD, 400 MHz) and ^13^C (CD_3_OD, 100 MHz) NMR spectra for **12a**; Figure S2: ^1^H (CD_3_OD, 400 MHz) and ^13^C (CD_3_OD, 100 MHz) NMR spectra for **12b**; Figure S3: ^1^H (CD_3_OD, 400 MHz) and ^13^C (CD_3_OD, 100 MHz) NMR spectra for **12c**; Figure S4: ^1^H (CD_3_OD, 400 MHz) and ^13^C (CD_3_OD, 100 MHz) NMR spectra for **12d**; Figure S5: ^1^H (CD_3_OD, 400 MHz) and ^13^C (CD_3_OD, 100 MHz) NMR spectra for **12e**; Figure S6: ^1^H (CD_3_OD, 400 MHz) and ^13^C (CD_3_OD, 100 MHz) NMR spectra for **12f**; Figure S7: ^1^H (CD_3_OD, 400 MHz) and ^13^C (CD_3_OD, 100 MHz) NMR spectra for **13b**; Figure S8: ^1^H (CD_3_OD, 400 MHz) and ^13^C (CD_3_OD, 100 MHz) NMR spectra for **13c**; Figure S9: ^1^H (CD_3_OD, 400 MHz) and ^13^C (CD_3_OD, 100 MHz) NMR spectra for **13d**; Figure S10: ^1^H (CD_3_OD, 400 MHz) and ^13^C (CD_3_OD, 100 MHz) NMR spectra for **13e**; Figure S11: ^1^H (CD_3_OD, 400 MHz) and ^13^C (CD_3_OD, 100 MHz) NMR spectra for **13f**; Figure S12: ^1^H (CD_3_OD, 400 MHz) and ^13^C (CD_3_OD, 100 MHz) NMR spectra for **14a**; Figure S13: ^1^H (CD_3_OD, 400 MHz) and ^13^C (CD_3_OD, 100 MHz) NMR spectra for **14b**; Figure S14: ^1^H (CD_3_OD, 400 MHz) and ^13^C (CD_3_OD, 100 MHz) NMR spectra for **14c**; Figure S15: ^1^H (CD_3_OD, 400 MHz) and ^13^C (CD_3_OD, 100 MHz) NMR spectra for **14d**; Figure S16: ^1^H (CD_3_OD, 400 MHz) and ^13^C (CD_3_OD, 100 MHz) NMR spectra for **14e**; Figure S17: ^1^H (CD_3_OD, 400 MHz) and ^13^C (CD_3_OD, 100 MHz) NMR spectra for **14f**; Figure S18: ^1^H (CD_3_OD, 400 MHz) and ^13^C (CD_3_OD, 100 MHz) NMR spectra for **15a**; Figure S19: ^1^H (CD_3_OD, 400 MHz) and ^13^C (CD_3_OD, 100 MHz) NMR spectra for **15b**; Figure S20: ^1^H (CD_3_OD, 400 MHz) and ^13^C (CD_3_OD, 100 MHz) NMR spectra for **15c**; Figure S21: ^1^H (CD_3_OD, 400 MHz) and ^13^C (CD_3_OD, 100 MHz) NMR spectra for **15d**; Figure S22: ^1^H (CD_3_OD, 400 MHz) and ^13^C (CD_3_OD, 100 MHz) NMR spectra for **15e**; Figure S23: ^1^H (CD_3_OD, 400 MHz) and ^13^C (CD_3_OD, 100 MHz) NMR spectra for **15f**; Figure S24: ^1^H (CD_3_OD, 400 MHz) and ^13^C (CD_3_OD, 100 MHz) NMR spectra for **16a**; Figure S25: ^1^H (CD_3_OD, 400 MHz) and ^13^C (CD_3_OD, 100 MHz) NMR spectra for **16b**; Figure S26: ^1^H (CD_3_OD, 400 MHz) and ^13^C (CD_3_OD, 100 MHz) NMR spectra for **16c**; Figure S27: ^1^H (CD_3_OD, 400 MHz) and ^13^C (CD_3_OD, 100 MHz) NMR spectra for **16d**; Figure S28: ^1^H (CD_3_OD, 400 MHz) and ^13^C (CD_3_OD, 100 MHz) NMR spectra for **16e**; Figure S29: ^1^H (CD_3_OD, 400 MHz) and ^13^C (CD_3_OD, 100 MHz) NMR spectra for **16f**; Figure S30: ^1^H (CD_3_OD, 400 MHz) and ^13^C (CD_3_OD, 100 MHz) NMR spectra for **17a**; Figure S31: ^1^H (CD_3_OD, 400 MHz) and ^13^C (CD_3_OD, 100 MHz) NMR spectra for **17b**; Figure S32: ^1^H (CD_3_OD, 400 MHz) and ^13^C (CD_3_OD, 100 MHz) NMR spectra for **17c**; Figure S33: ^1^H (CD_3_OD, 400 MHz) and ^13^C (CD_3_OD, 100 MHz) NMR spectra for **17d**; Figure S34: ^1^H (CD_3_OD, 400 MHz) and ^13^C (CD_3_OD, 100 MHz) NMR spectra for **17e**; Figure S35: ^1^H (CD_3_OD, 400 MHz) and ^13^C (CD_3_OD, 100 MHz) NMR spectra for **17f**; Figure S36: ^1^H (CD_3_OD, 400 MHz) and ^13^C (CD_3_OD, 100 MHz) NMR spectra for **18a**; Figure S37: ^1^H (CD_3_OD, 400 MHz) and ^13^C (CD_3_OD, 100 MHz) NMR spectra for **18b**; Figure S38: ^1^H (CD_3_OD, 400 MHz) and ^13^C (CD_3_OD, 100 MHz) NMR spectra for **18c**; Figure S39: ^1^H (CD_3_OD, 400 MHz) and ^13^C (CD_3_OD, 100 MHz) NMR spectra for **18d**; Figure S40: ^1^H (CD_3_OD, 400 MHz) and ^13^C (CD_3_OD, 100 MHz) NMR spectra for **18e**; Figure S41: ^1^H (CD_3_OD, 400 MHz) and ^13^C (CD_3_OD, 100 MHz) NMR spectra for **18f**.
