# Supplementary material for: Antimicrobial Indole-3-Carboxamido-Polyamine Conjugates Target Bacterial Membranes and Are Antibiotic Potentiators
Source: Biomolecules. 2024 Feb 22;14(3):261. doi: 10.3390/biom14030261 (PMC10967822; doi:10.3390/biom14030261)
Supplement: Supplementary file 1 [file biomolecules-14-00261-s001.zip › supplemetary File S1.pdf]

Supplementary File

Antimicrobial Indole-3-carboxamido-Polyamine Conjugates Target Bacterial Membranes and are Antibiotic Potentiators

Kenneth Sue [1], Melissa M. Cadelis [1,2], Florent Rouvier [3], Marie-Lise Bourguet-Kondracki [4], Jean Michel Brunel [3] and Brent R. Copp [1,*]

[1] School of Chemical Sciences, The University of Auckland, Private Bag 92019, Auckland 1142, New Zealand
[2] School of Medical Sciences, The University of Auckland, Private Bag 92019, Auckland 1142, New Zealand
[3] Membranes et Cibles Thérapeutiques, INSERM, Aix-Marseille Universite, 27 bd Jean Moulin, 13385 Marseille, France
[4] Laboratoire Molécules de Communication et Adaptation des Micro-organismes, UMR 7245 CNRS, Muséum National d'Histoire Naturelle, 57 rue Cuvier (C.P. 54), 75005 Paris, France
* Correspondence: b.copp@auckland.ac.nz

Materials and Methods

Synthesis of compounds

N^1,N^4-Bis(3-(1*H*-indole-3-carboxamido)propyl)butane-1,4-diaminium 2,2,2-trifluoroacetate (**12a**)

Following general procedure A, reaction of 1*H*-indole-3-carboxylic acid (0.050 g, 0.310 mmol) with EDC·HCl (0.070 g, 0.367 mmol), HOBt (0.047 g, 0.367 mmol), DIPEA (0.15 mL, 0.846 mmol) and di-*tert*-butyl butane-1,4-diylbis((3-aminopropyl)carbamate) **11a** (0.057 g, 0.141 mmol) afforded di-*tert*-butyl butane-1,4-diylbis((3-(1*H*-indole-3-carboxamido)propyl)carbamate) (0.032 g, 33%) as a clear colorless oil. Following general procedure B, a subsample of this product (0.016 g, 0.023 mmol) was reacted with TFA in CH_2Cl_2 to afford, after chromatography, the di-TFA salt **12a** (0.013 g, 78%) as a brown oil. R_f (RP-18, 10% *aq* HCl:MeOH 1:3) 0.65; IR (ATR) ν_{max} 3267, 2828, 1671, 1621, 1575, 1544, 1494, 1433, 1340, 1320, 1274, 1198, 1182, 1130, 1010, 836, 800, 749, 721 cm^{-1}; ^{1}H NMR (CD_3OD, 400 MHz) δ 8.10 (2H, d, *J* = 7.5 Hz, H-4), 7.90 (2H, s, H-2), 7.43 (2H, d, *J* = 7.8 Hz, H-7), 7.17 (4H, dd, *J* = 7.8, 7.5 Hz, H-5, H-6), 3.52 (4H, t, *J* = 6.2 Hz, H_2-10), 3.09–3.06 (8H, m, H_2-12, H_2-14), 1.99 (4H, tt, *J* = 6.6, 6.5 Hz, H_2-11), 1.89 (4H, tt, *J* = 3.6, 3.5 Hz, H_2-15); ^{13}C NMR (CD_3OD, 100 MHz) δ 169.7 (C-8), 138.1 (C-7a), 129.4 (C-2), 127.1 (C-3a), 123.6 (C-6), 122.2 (C-5), 121.7 (C-4), 112.9 (C-7), 111.0 (C-3), 48.1 (C-14), 46.2 (C-12), 36.5 (C-10), 28.2 (C-11), 24.4 (C-15); (+)-HRESIMS $[M+H]^+$ *m/z* 489.2972 (calcd for $C_{28}H_{37}N_6O_2$, 489.2973).

N^1,N^6-Bis(3-(1*H*-indole-3-carboxamido)propyl)hexane-1,6-diaminium 2,2,2-trifluoroacetate (**12b**)

Following general procedure A, reaction of 1*H*-indole-3-carboxylic acid (0.050 g, 0.310 mmol) with EDC·HCl (0.070 g, 0.367 mmol), HOBt (0.047 g, 0.367 mmol), DIPEA (0.15 mL, 0.846 mmol) and di-*tert*-butyl hexane-1,6-diylbis((3-aminopropyl)carbamate) **11b** (0.061 g, 0.141 mmol) afforded di-*tert*-butyl hexane-1,6-diylbis((3-(1*H*-indole-3-carboxamido)propyl)carbamate) (0.033 g, 33%) as a clear colorless oil. Following general procedure B, a subsample of this product (0.016 g, 0.022 mmol) was reacted with TFA in CH_2Cl_2 to afford, after chromatography, the di-TFA salt **12b** (0.015 g, 90%) as a yellow oil. R_f (RP-18, 10% *aq* HCl:MeOH 1:3) 0.65; IR (ATR) ν_{max} 3272, 2830, 1671, 1621, 1577, 1543, 1494, 1433, 1340, 1320, 1274, 1198, 1181, 1129, 1010, 835, 800, 749, 721 cm^{-1}; ^{1}H NMR (CD_3OD, 400 MHz) δ 8.11 (2H, d, *J* = 7.5 Hz, H-4), 7.91 (2H, s, H-2), 7.43 (2H, d, *J* = 7.5 Hz, H-7), 7.18 (2H, dd, *J* = 7.6, 7.5 Hz, H-6), 7.15 (2H, dd, *J* = 7.6, 7.5 Hz, H-5), 3.51 (4H, t, *J* = 6.1 Hz, H_2-10), 3.03 (4H, t, *J* = 6.9 Hz, H_2-12), 2.98 (4H, t, *J* = 7.6

Hz, H_2-14), 1.98 (4H, tt, *J* = 6.5, 6.5 Hz, H_2-11), 1.77–1.70 (4H, m, H_2-15), 1.50–1.45 (4H, m, H_2-16); ^{13}C NMR (CD_3OD, 100 MHz) δ 169.6 (C-8), 138.1 (C-7a), 129.4 (C-2), 127.1 (C-3a), 123.6 (C-6), 122.1 (C-5), 121.7 (C-4), 112.9 (C-7), 111.0 (C-3), 48.7 (C-14), 46.2 (C-12), 36.5 (C-10), 28.1 (C-11), 27.04 (C-16), 27.00 (C-15); (+)-HRESIMS $[M+H]^+$ *m/z* 517.3287 (calcd for $C_{30}H_{41}N_6O_2$, 517.3286).

N^1,N^7-Bis(3-(1*H*-indole-3-carboxamido)propyl)heptane-1,7-diaminium 2,2,2-trifluoroacetate (**12c**)

Following general procedure A, reaction of 1*H*-indole-3-carboxylic acid (0.050 g, 0.310 mmol) with EDC·HCl (0.070 g, 0.367 mmol), HOBt (0.047 g, 0.367 mmol), DIPEA (0.15 mL, 0.846 mmol) and di-*tert*-butyl heptane-1,7-diylbis((3-aminopropyl)carbamate) **11c** (0.063 g, 0.141 mmol) afforded di-*tert*-butyl heptane-1,7-diylbis((3-(1*H*-indole-3-carboxamido)propyl)carbamate) (0.073 g, 71%) as a clear colorless oil. Following general procedure B, a subsample of this product (0.036 g, 0.049 mmol) was reacted with TFA in CH_2Cl_2 to afford, after chromatography, the di-TFA salt **12c** (0.037 g, 99%) as a yellow oil. R_f (RP-18, 10% *aq* HCl:MeOH 1:3) 0.63; IR (ATR) ν_{max} 3262, 2827, 1671, 1621, 1577, 1543, 1494, 1433, 1339, 1320, 1273, 1198, 1181, 1128, 1010, 940, 835, 799, 749, 721 cm^{-1}; ^{1}H NMR (CD_3OD, 400 MHz) δ 8.10 (2H, d, *J* = 8.1 Hz, H-4), 7.92 (2H, s, H-2), 7.44 (2H, d, *J* = 7.8 Hz, H-7), 7.19 (2H, dd, *J* = 7.8, 7.3 Hz, H-6), 7.15 (2H, dd, *J* = 8.1, 7.3 Hz, H-5), 3.51 (4H, t, *J* = 6.3 Hz, H_2-10), 3.04 (4H, t, *J* = 7.0 Hz, H_2-12), 2.97 (4H, t, *J* = 7.7 Hz, H_2-14), 1.98 (4H, tt, *J* = 6.6, 6.6 Hz, H_2-11), 1.72 (4H, tt, *J* = 7.1, 6.9 Hz, H_2-15), 1.46–1.40 (6H, m, H_2-16, H_2-17); ^{13}C NMR (CD_3OD, 100 MHz) δ 169.6 (C-8), 138.1 (C-7a), 129.4 (C-2), 127.1 (C-3a), 123.6 (C-6), 122.1 (C-5), 121.7 (C-4), 112.9 (C-7), 111.0 (C-3), 49.8 (C-14), 46.1 (C-12), 36.6 (C-10), 29.6 (C-17), 28.1 (C-11), 27.2 (C-16), 27.1 (C-15); (+)-HRESIMS $[M+H]^+$ *m/z* 531.3443 (calcd for $C_{31}H_{43}N_6O_2$, 531.3342).

N^1,N^8-Bis(3-(1*H*-indole-3-carboxamido)propyl)octane-1,8-diaminium 2,2,2-trifluoroacetate (**12d**)

Following general procedure A, reaction of 1*H*-indole-3-carboxylic acid (0.050 g, 0.310 mmol) with EDC·HCl (0.070 g, 0.367 mmol), HOBt (0.047 g, 0.367 mmol), DIPEA (0.15 mL, 0.846 mmol) and di-*tert*-butyl octane-1,8-diylbis((3-aminopropyl)carbamate) **11d** (0.065 g, 0.141 mmol) afforded di-*tert*-butyl octane-1,8-diylbis((3-(1*H*-indole-3-carboxamido)propyl)carbamate) (0.034 g, 32%) as a clear colorless oil. Following general procedure B, a subsample of this product (0.017 g, 0.023 mmol) was reacted with TFA in CH_2Cl_2 to afford, after chromatography, the di-TFA salt **12d** (0.017 g, 96%) as a yellow oil. R_f (RP-18, 10% *aq* HCl:MeOH 1:3) 0.60; IR (ATR) ν_{max} 3269, 2828, 1671, 1621, 1576, 1543, 1494, 1433, 1339, 1320, 1274, 1198, 1181, 1128, 1010, 940, 835, 799, 749, 721 cm^{-1}; ^{1}H NMR (CD_3OD, 400 MHz) δ 8.11 (2H, d, *J* = 7.7 Hz, H-4), 7.91 (2H, s, H-2), 7.43 (2H, d, *J* = 7.8 Hz, H-7), 7.19 (2H, dd, *J* = 8.0, 7.8 Hz, H-6), 7.15 (2H, dd, *J* = 8.0, 7.7 Hz, H-5), 3.52 (4H, t, *J* = 6.1 Hz, H_2-10), 3.04 (4H, t, *J* = 6.9 Hz, H_2-12), 2.98 (4H, t, *J* = 7.6 Hz, H_2-14), 1.98 (4H, tt, *J* = 6.4, 6.4 Hz, H_2-11), 1.73 (4H, tt, *J* = 6.4, 6.4 Hz, H_2-15), 1.47–1.36 (8H, m, H_2-16, H_2-17); ^{13}C NMR (CD_3OD, 100 MHz) δ 169.6 (C-8), 138.1 (C-7a), 129.4 (C-2), 127.1 (C-3a), 123.6 (C-6), 122.1 (C-5), 121.7 (C-4), 112.9 (C-7), 111.0 (C-3), 49.8 (C-14), 46.2 (C-12), 36.5 (C-10), 29.9 (C-17), 28.2 (C-11), 27.4 (C-16), 27.3 (C-15); (+)-HRESIMS $[M+H]^+$ *m/z* 545.3599 (calcd for $C_{32}H_{45}N_6O_2$, 545.3599).

N^1,N^{10}-Bis(3-(1*H*-indole-3-carboxamido)propyl)decane-1,10-diaminium 2,2,2-trifluoroacetate (**12e**)

Following general procedure A, reaction of 1*H*-indole-3-carboxylic acid (0.050 g, 0.310 mmol) with EDC·HCl (0.070 g, 0.367 mmol), HOBt (0.047 g, 0.367 mmol), DIPEA (0.15 mL, 0.846 mmol) and di-*tert*-butyl decane-1,10-diylbis((3-aminopropyl)carbamate) **11e** (0.069 g, 0.141 mmol) afforded di-*tert*-butyl decane-1,10-diylbis((3-(1*H*-indole-3-carboxamido)propyl)carbamate) (0.031 g, 28%) as a clear colorless oil. Following general procedure B, a subsample of this product (0.015 g, 0.019 mmol) was reacted with TFA in CH_2Cl_2 to afford, after chromatography, the di-TFA salt **12e** (0.009 g, 58%) as a yellow oil. R_f (RP-18, 10% *aq* HCl:MeOH 1:3) 0.50; IR (ATR) ν_{max} 2932, 2957, 1674, 1622, 1543, 1435, 1321, 1201, 1133, 836, 800, 750, 721 cm^{-1}; ^{1}H NMR (CD_3OD, 400 MHz) δ 8.11 (2H, d, *J* = 7.4 Hz, H-4), 7.91 (2H, s, H-2), 7.44 (2H, d, *J* = 7.3 Hz, H-7), 7.22–7.17 (2H, m, H-6), 7.17–7.13 (2H, m, H-5), 3.52 (4H, t, *J* = 6.3 Hz, H_2-10), 3.05 (4H, t, *J* = 6.9 Hz, H_2-12), 2.99 (4H, t, *J* = 7.8 Hz, H_2-14), 1.98 (4H, tt, *J* = 6.6, 6.6 Hz, H_2-11), 1.73 (4H, tt, *J* = 7.6, 7.5 Hz, H_2-15), 1.48–1.40 (4H, m, H_2-16), 1.40–1.34 (8H, m, H_2-17, H_2-18); ^{13}C NMR (CD_3OD, 100 MHz) δ 169.7 (C-8), 138.1 (C-7a), 129.3 (C-2), 127.1 (C-3a), 123.6 (C-6), 122.1 (C-5), 121.7 (C-4), 112.9 (C-7), 111.0 (C-3), 48.9 (C-14, obscured by solvent), 46.2 (C-12), 36.5 (C-10), 30.3 (C-18), 30.2 (C-17), 28.2 (C-11), 27.5 (C-16), 27.4 (C-15); (+)-HRESIMS $[M+H]^+$ *m/z* 573.3916 (calcd for $C_{34}H_{49}N_6O_2$, 573.3912).

N[1],*N*[12]-Bis(3-(1*H*-indole-3-carboxamido)propyl)dodecane-1,12-diaminium 2,2,2-trifluoroacetate (**12f**)

Following general procedure A, reaction of 1*H*-indole-3-carboxylic acid (0.050 g, 0.310 mmol) with EDC·HCl (0.070 g, 0.367 mmol), HOBt (0.047 g, 0.367 mmol), DIPEA (0.15 mL, 0.846 mmol) and di-*tert*-butyl dodecane-1,12-diylbis((3-aminopropyl)carbamate) **11f** (0.073 g, 0.141 mmol) afforded di-*tert*-butyl dodecane-1,12-diylbis((3-(1*H*-indole-3-carboxamido)propyl)carbamate) (0.068 g, 60%) as a clear colorless oil. Following general procedure B, a subsample of this product (0.034 g, 0.042 mmol) was reacted with TFA in CH_2Cl_2 to afford, after chromatography, the di-TFA salt **12f** (0.014 g, 40%) as a yellow oil. R_f (RP-18, 10% *aq* HCl:MeOH 1:3) 0.40; IR (ATR) ν_{max} 2931, 2857, 1674, 1622, 1544, 1435, 1321, 1201, 1133, 836, 800, 750, 722 cm^{-1}; ^1H NMR (CD_3OD, 400 MHz) δ 8.11 (2H, d, *J* = 7.4 Hz, H-4), 7.91 (2H, s, H-2), 7.44 (2H, d, *J* = 8.2 Hz, H-7), 7.22–7.17 (2H, m, H-6), 7.17–7.13 (2H, m, H-5), 3.53 (4H, t, *J* = 6.3 Hz, H_2-10), 3.06 (4H, t, *J* = 6.9 Hz, H_2-12), 2.99 (4H, t, *J* = 7.6 Hz, H_2-14), 1.99 (4H, tt, *J* = 6.6, 6.6 Hz, H_2-11), 1.73 (4H, tt, *J* = 7.6, 7.5 Hz, H_2-15), 1.47–1.39 (4H, m, H_2-16), 1.39–1.30 (12H, m, H_2-17, H_2-18, H_2-19); ^{13}C NMR (CD_3OD, 100 MHz) δ 169.7 (C-8), 138.1 (C-7a), 129.3 (C-2), 127.1 (C-3a), 123.6 (C-6), 122.1 (C-5), 121.7 (C-4), 112.9 (C-7), 111.0 (C-3), 48.9 (C-14, obscured by solvent), 46.2 (C-12), 36.5 (C-10), 30.6 (C-19), 30.5 (C-18), 30.2 (C-17), 28.2 (C-11), 27.5 (C-16), 27.4 (C-15); (+)-HRESIMS $[M+H]^+$ *m/z* 601.4226 (calcd for $C_{36}H_{53}N_6O_2$, 601.4225).

N[1],*N*[6]-Bis(3-(5-bromo-1*H*-indole-3-carboxamido)propyl) hexane-1,6-diaminium 2,2,2-trifluoroacetate (**13b**)

Following general procedure A, reaction of 5-bromo-1*H*-indole-3-carboxylic acid (0.100 g, 0.416 mmol) with EDC·HCl (0.047 g, 0.246 mmol), HOBt (0.032 g, 0.246 mmol), DIPEA (0.10 mL, 0.568 mmol) and di-*tert*-butyl hexane-1,6-diylbis((3-aminopropyl)carbamate) **11b** (0.082 g, 0.189 mmol) afforded di-*tert*-butyl hexane-1,6-diylbis((3-(5-bromo-1*H*-indole-3-carboxamido)propyl)carbamate) (0.105 g, 63%) as a clear pale yellow oil. Following general procedure B, a subsample of this product (0.054 g, 0.062 mmol) was reacted with TFA in CH_2Cl_2 to afford, after chromatography, the di-TFA salt **13b** (0.056 g, 100%) as a red oil. R_f (RP-18, 10% *aq* HCl:MeOH 1:3) 0.45; IR (ATR) ν_{max} 3283, 2941, 2863, 1671, 1616, 1568, 1543, 1446, 1432, 1347, 1327, 1300, 1263, 1198, 1182, 1131, 1050, 887, 836, 800, 772, 721 cm^{-1}; ^1H NMR (CD_3OD, 400 MHz) δ 8.29 (2H, d, *J* = 1.9 Hz, H-4), 7.94 (2H, s, H-2), 7.35 (2H, d, *J* = 8.3 Hz, H-7), 7.27 (2H, dd, *J* = 8.6, 1.9 Hz, H-6), 3.50 (4H, t, *J* = 6.3 Hz, H_2-10), 3.05–2.99 (8H, m, H_2-12 and H_2-14), 1.97 (4H, tt, *J* = 6.6, 6.5 Hz, H_2-11), 1.77 (4H, tt, *J* = 7.3, 7.3 Hz, H_2-15), 1.51 (4H, tt, *J* = 6.5, 3.6 Hz, H_2-16); ^{13}C NMR (CD_3OD, 100 MHz) δ 168.9 (C-8), 136.7 (C-7a), 130.3 (C-2), 129.1 (C-3a), 126.4 (C-6), 124.5 (C-4), 115.5 (C-5), 114.6 (C-7), 110.6 (C-3), 49.8 (C-14), 46.1 (C-12), 36.5 (C-10), 28.0 (C-11), 27.1 (C-15 and C-16); (+)-HRESIMS $[M+H]^+$ *m/z* 673.1492 (calcd for $C_{30}H_{39}{}^{79}Br_2N_6O_2$, 673.1496), 675.1453 (calcd for $C_{30}H_{39}{}^{79}Br^{81}BrN_6O_2$, 675.1478), 677.1458 (calcd for $C_{30}H_{39}{}^{81}Br_2N_6O_2$, 677.1464).

N[1],*N*[7]-Bis(3-(5-bromo-1*H*-indole-3-carboxamido)propyl) heptane-1,7-diaminium 2,2,2-trifluoroacetate (**13c**)

Following general procedure A, reaction of 5-bromo-1*H*-indole-3-carboxylic acid (0.050 g, 0.208 mmol) with EDC·HCl (0.047 g, 0.246 mmol), HOBt (0.032 g, 0.246 mmol), DIPEA (0.10 mL, 0.568 mmol) and di-*tert*-butyl heptane-1,7-diylbis((3-aminopropyl)carbamate) **11c** (0.042 g, 0.097 mmol) afforded di-*tert*-butyl heptane-1,7-diylbis((3-(5-bromo-1*H*-indole-3-carboxamido)propyl)carbamate) (0.028 g, 33%) as a clear colorless oil. Following general procedure B, a subsample of this product (0.023 g, 0.026 mmol) was reacted with TFA in CH_2Cl_2 to afford, after chromatography, the di-TFA salt **13c** (0.015 g, 63%) as a clear colorless oil. R_f (RP-18, 10% *aq* HCl:MeOH 1:3) 0.40; IR (ATR) ν_{max} 3268, 2942, 2963, 1671, 1616, 1568, 1543, 1447, 1432, 1347, 1329, 1300, 1263, 1198, 1182, 1131, 1050, 887, 836, 800, 772, 721 cm^{-1}; ^1H NMR (CD_3OD, 400 MHz) δ 8.31 (2H, d, *J* = 1.8 Hz, H-4), 7.93 (2H, s, H-2), 7.36 (2H, d, *J* = 8.8 Hz, H-7), 7.29 (2H, dd, *J* = 8.6, 6.9 Hz, H-6), 3.51 (4H, t, *J* = 6.3 Hz, H_2-10), 3.05 (4H, t, *J* = 6.8 Hz, H_2-12), 3.02 (4H, t, *J* = 7.7 Hz, H_2-14), 1.97 (4H, tt, *J* = 6.5, 6.4 Hz, H_2-11), 1.78 (4H, tt, *J* = 6.9, 6.8 Hz, H_2-15), 1.55–1.49 (6H, m, H_2-16, H_2-17); ^{13}C NMR (CD_3OD, 100 MHz) δ 169.1 (C-8), 136.7 (C-7a), 130.3 (C-2), 129.1 (C-3a), 126.5 (C-6), 124.6 (C-4), 115.5 (C-5), 114.6 (C-7), 110.6 (C-3), 48.6 (C-14, obscured by solvent), 46.1 (C-12), 36.4 (C-10), 29.8 (C-17), 28.2 (C-11), 27.4 (C-16), 27.3 (C-15); (+)-HRESIMS $[M+H]^+$ *m/z* 687.1660 (calcd for

$C_{31}H_{41}{}^{79}Br_2N_6O_2$, 687.1652), 689.1517 (calcd for $C_{31}H_{41}{}^{79}Br^{81}BrN_6O_2$, 689.1517), 691.1634 (calcd for $C_{31}H_{41}{}^{81}Br_2N_6O_2$, 691.1621).

N[1],*N*[8]-Bis(3-(5-bromo-1*H*-indole-3-carboxamido)propyl) octane-1,8-diaminium 2,2,2-trifluoroacetate (**13d**)

Following general procedure A, reaction of 5-bromo-1*H*-indole-3-carboxylic acid (0.050 g, 0.208 mmol) with EDC·HCl (0.047 g, 0.246 mmol), HOBt (0.032 g, 0.246 mmol), DIPEA (0.10 mL, 0.568 mmol) and di-*tert*-butyl octane-1,8-diylbis((3-aminopropyl)carbamate) **11d** (0.043 g, 0.097 mmol) afforded di-*tert*-butyl octane-1,8-diylbis((3-(5-bromo-1*H*-indole-3-carboxamido)propyl)carbamate) (0.025 g, 29%) as a clear colorless oil. Following general procedure B, a subsample of this product (0.012 g, 0.013 mmol) was reacted with TFA in CH_2Cl_2 to afford, after chromatography, the di-TFA salt **13d** (0.009 g, 73%) as a clear colorless oil. R_f (RP-18, 10% *aq* HCl:MeOH 1:3) 0.33; IR (ATR) ν_{max} 3280, 2942, 2864, 1671, 1616, 1568, 1543, 1446, 1433, 1347, 1326, 1300, 1263, 1198, 1181, 1131, 1050, 887, 836, 799, 772, 721 cm^{-1}; ^1H NMR (CD_3OD, 400 MHz) δ 8.31 (2H, d, *J* = 1.8 Hz, H-4), 7.94 (2H, s, H-2), 7.36 (2H, d, *J* = 8.8 Hz, H-7), 7.29 (2H, dd, *J* = 8.7, 1.9 Hz, H-6), 3.51 (4H, t, *J* = 6.2 Hz, H_2-10), 3.04 (4H, t, *J* = 7.1 Hz, H_2-12), 3.00 (4H, t, *J* = 7.7 Hz, H_2-14), 1.97 (4H, tt, *J* = 6.6, 6.6 Hz, H_2-11), 1.75 (4H, tt, *J* = 7.6, 7.6 Hz, H_2-15), 1.51–1.41 (8H, m, H_2-16, H_2-17); ^{13}C NMR (CD_3OD, 100 MHz) δ 169.1 (C-8), 136.7 (C-7a), 130.3 (C-2), 129.1 (C-3a), 126.5 (C-6), 124.6 (C-4), 115.5 (C-5), 114.6 (C-7), 110.6 (C-3), 49.2 (C-14), 46.1 (C-12), 36.5 (C-10), 30.0 (C-17), 28.2 (C-11), 27.5 (C-16), 27.4 (C-15); (+)-HRESIMS $[M+H]^+$ *m/z* 701.1813 (calcd for $C_{32}H_{43}{}^{79}Br_2N_6O_2$, 701.1089), 703.1640 (calcd for $C_{32}H_{43}{}^{79}Br^{81}BrN_6O_2$, 703.1791), 705.1789 (calcd for $C_{32}H_{43}{}^{81}Br_2N_6O_2$, 705.1778).

N[1],*N*[10]-Bis(3-(5-bromo-1*H*-indole-3-carboxamido)propyl) decane-1,10-diaminium 2,2,2-trifluoroacetate (**13e**)

Following general procedure A, reaction of 5-bromo-1*H*-indole-3-carboxylic acid (0.050 g, 0.208 mmol) with EDC·HCl (0.047 g, 0.246 mmol), HOBt (0.032 g, 0.246 mmol), DIPEA (0.10 mL, 0.568 mmol) and di-*tert*-butyl decane-1,10-diylbis((3-aminopropyl)carbamate) **11e** (0.046 g, 0.097 mmol) afforded di-*tert*-butyl decane-1,10-diylbis((3-(5-bromo-1*H*-indole-3-carboxamido)propyl)carbamate) (0.052 g, 59%) as a clear colorless oil. Following general procedure B, a subsample of this product (0.026 g, 0.028 mmol) was reacted with TFA in CH_2Cl_2 to afford, after chromatography, the di-TFA salt **13e** (0.008 g, 30%) as a clear colorless oil. R_f (RP-18, 10% *aq* HCl:MeOH 1:3) 0.23; IR (ATR) ν_{max} 3228, 2937, 2856, 1672, 1557, 1513,1434, 1344, 1325, 1301, 1263, 1191, 1132, 886, 864, 836, 799, 770, 719 cm^{-1}; ^1H NMR (CD_3OD, 400 MHz) δ 8.31 (2H, d, *J* = 1.8 Hz, H-4), 7.94 (2H, s, H-2), 7.36 (2H, d, *J* = 8.8 Hz, H-7), 7.29 (2H, dd, *J* = 8.6, 1.9 Hz, H-6), 3.51 (4H, t, *J* = 6.4 Hz, H_2-10), 3.05 (4H, t, *J* = 7.1 Hz, H_2-12), 3.00 (4H, t, *J* = 7.6 Hz, H_2-14), 1.98 (4H, tt, *J* = 6.8, 6.5 Hz, H_2-11), 1.73 (4H, tt, *J* = 7.5, 7.5 Hz, H_2-15), 1.47–1.35 (12H, m, H_2-16, H_2-17, H_2-18); ^{13}C NMR (CD_3OD, 100 MHz) δ 169.0 (C-8), 136.7 (C-7a), 130.3 (C-2), 129.1 (C-3a), 126.5 (C-6), 124.6 (C-4), 115.5 (C-5), 114.6 (C-7), 110.6 (C-3), 49.8 (C-14), 46.1 (C-12), 36.5 (C-10), 30.4 (C-18), 30.2 (C-17), 28.2 (C-11), 27.6 (C-16), 27.4 (C-15); (+)-HRESIMS $[M+H]^+$ *m/z* 729.2121 (calcd for $C_{34}H_{47}{}^{79}Br_2N_6O_2$, 729.2122), 731.2092 (calcd for $C_{34}H_{47}{}^{79}Br^{81}BrN_6O_2$, 731.2104), 733.2087 (calcd for $C_{34}H_{47}{}^{81}Br_2N_6O_2$, 733.2092).

N^1,N^{12}-Bis(3-(5-bromo-1*H*-indole-3-carboxamido)propyl) dodecane-1,12-diaminium 2,2,2-trifluoroacetate (**13f**)

Following general procedure A, reaction of 5-bromo-1*H*-indole-3-carboxylic acid (0.050 g, 0.208 mmol) with EDC·HCl (0.047 g, 0.246 mmol), HOBt (0.032 g, 0.246 mmol), DIPEA (0.10 mL, 0.568 mmol) and di-*tert*-butyl dodecane-1,12-diylbis((3-aminopropyl)carbamate) **11f** (0.049 g, 0.097 mmol) afforded di-*tert*-butyl dodecane-1,12-diylbis((3-(5-bromo-1*H*-indole-3-carboxamido)propyl)carbamate) (0.032 g, 35%) as a clear colorless oil. Following general procedure B, a subsample of this product (0.016 g, 0.017 mmol) was reacted with TFA in CH_2Cl_2 to afford, after chromatography, the di-TFA salt **13f** (0.011 g, 67%) as a clear colorless oil. R_f (RP-18, 10% *aq* HCl:MeOH 1:3) 0.15; IR (ATR) ν_{max} 3235, 3024, 2938, 2851, 1670, 1614, 1557, 1433, 1343, 1325, 1301, 1263, 1192, 1133, 886, 864, 836, 800, 769, 719 cm^{-1}; ^1H NMR (CD_3OD, 400 MHz) δ 8.31 (2H, d, *J* = 1.8 Hz, H-4), 7.94 (2H, s, H-2), 7.36 (2H, d, *J* = 8.3 Hz, H-7), 7.29 (2H, dd, *J* = 8.7, 1.9 Hz, H-6), 3.51 (4H, t, *J* = 6.3 Hz, H_2-10), 3.05 (4H, t, *J* = 7.6 Hz, H_2-12), 3.00 (4H, t, *J* = 7.6 Hz, H_2-14), 1.98 (4H, tt, *J* = 6.6, 6.5 Hz, H_2-11), 1.73 (4H, tt, *J* = 7.6, 7.5 Hz, H_2-15), 1.48–1.38 (4H, m, H_2-16), 1.38–1.31 (12H, m, H_2-17, H_2-18, H_2-19); ^{13}C NMR (CD_3OD, 100 MHz) δ 169.1 (C-8), 136.8 (C-7a), 130.3 (C-2), 129.2 (C-3a), 126.5 (C-6), 124.6 (C-4), 115.6 (C-5), 114.6 (C-7), 110.7 (C-3), 49.1 (C-14), 46.2 (C-12), 36.5 (C-10), 30.6 (C-19), 30.5 (C-18), 28.2 (C-11), 27.6 (C-17), 27.5 (C-16), 27.4 (C-15); (+)-HRESIMS $[M+H]^+$ *m/z* 757.2430 (calcd for $C_{36}H_{51}{}^{79}Br_2N_6O_2$, 757.2435), 759.2378 (calcd for $C_{36}H_{51}{}^{79}Br^{81}BrN_6O_2$, 759.2418), 761.2400 (calcd for $C_{36}H_{51}{}^{81}Br_2N_6O_2$, 761.2406).

N^1,N^4-Bis(3-(5-methoxy-1*H*-indole-3-carboxamido)propyl)butane-1,4-diaminium 2,2,2-trifluoroacetate (**14a**)

Following general procedure A, reaction of 5-methoxy-1*H*-indole-3-carboxylic acid (0.040 g, 0.209 mmol) with EDC·HCl (0.047 g, 0.247 mmol), HOBt (0.032 g, 0.247 mmol), DIPEA (0.10 mL, 0.571 mmol) and di-*tert*-butyl butane-1,4-diylbis((3-aminopropyl)carbamate) **11a** (0.038 g, 0.095 mmol) afforded di-*tert*-butyl butane-1,4-diylbis((3-(5-methoxy-1*H*-indole-3-carboxamido)propyl)carbamate) (0.034 g, 48%) as a clear colorless oil. Following general procedure B, a subsample of this product (0.017 g, 0.023 mmol) was reacted with TFA in CH_2Cl_2 to afford, after chromatography, the di-TFA salt **14a** (0.012, 68%) as a brown oil. R_f (RP-18, 10% *aq* HCl:MeOH 1:3) 0.67; IR (ATR) ν_{max} 3320, 1677, 1624, 1579, 1546, 1481, 1467, 1431, 1246, 1189, 1127, 1089, 1044, 1022, 920, 838, 822, 798, 777, 721 cm^{-1}; ^1H NMR (CD_3OD, 400 MHz) δ 7.86 (2H, s, H-2), 7.63 (2H, d, *J* = 2.4 Hz, H-4), 7.32 (2H, d, *J* = 8.8 Hz, H-7), 6.85 (2H, dd, *J* = 8.8, 2.5 Hz, H-6), 3.81 (6H, s, OMe), 3.51 (4H, t, *J* = 6.4 Hz, H_2-10), 3.08 (8H, t, *J* = 7.0 Hz, H_2-12, H_2-14), 1.99 (4H, tt, *J* = 6.4, 6.4 Hz, H_2-11), 1.88 (4H, tt, *J* = 3.5, 3.5 Hz, H_2-15); ^{13}C NMR (CD_3OD, 100 MHz) δ 169.8 (C-8), 156.7 (C-5), 133.2 (C-7a), 129.7 (C-2), 127.9 (C-3a), 113.6 (C-6/C-7), 113.5 (C-6/C-7), 110.6 (C-3), 104.1 (C-4), 56.2 (OMe), 48.0 (C-14), 46.2 (C-12), 36.5 (C-10), 28.2 (C-11), 24.4 (C-15); (+)-HRESIMS $[M+H]^+$ *m/z* 549.3185 (calcd for $C_{30}H_{41}N_6O_4$, 549.3184).

N^1,N^6-Bis(3-(5-methoxy-1*H*-indole-3-carboxamido)propyl) hexane-1,6-diaminium 2,2,2-trifluoroacetate (**14b**)

Following general procedure A, reaction of 5-methoxy-1*H*-indole-3-carboxylic acid (0.040 g, 0.209 mmol) with EDC·HCl (0.047 g, 0.247 mmol), HOBt (0.032 g, 0.247 mmol), DIPEA (0.10 mL, 0.571 mmol) and di-*tert*-butyl hexane-1,6-diylbis((3-aminopropyl)carbamate) **11b** (0.041 g, 0.095 mmol) afforded di-*tert*-butyl hexane-1,6-diylbis((3-(5-methoxy-1*H*-indole-3-carboxamido)propyl)carbamate) (0.054 g, 73%) as a clear colorless oil. Following general procedure B, a subsample of this product (0.027 g, 0.035 mmol) was reacted with TFA in CH_2Cl_2 to afford, after chromatography, the di-TFA salt **14b** (0.021 g, 75%) as a red-brown oil. R_f (RP-18, 10% *aq* HCl:MeOH 1:3) 0.65; IR (ATR) ν_{max} 3288, 1673, 1622, 1540, 1471, 1436, 1281, 1199, 1131, 1027, 924, 835, 800, 773, 721 cm^{-1}; ^1H NMR (CD_3OD, 400 MHz) δ 7.87 (2H, s, H-2), 7.65 (2H, d, *J* = 2.5 Hz, H-4), 7.32 (2H, d, *J* = 8.9 Hz, H-7), 6.84 (2H, dd, *J* = 8.8, 2.5 Hz, H-6), 3.82 (6H, s, OMe), 3.51 (4H, t, *J* = 6.3 Hz, H_2-10), 3.04 (4H, t, *J* = 7.0 Hz, H_2-12), 2.97 (4H, t, *J* = 7.7 Hz, H_2-14), 1.98 (4H, tt, *J* = 6.7, 6.6 Hz, H_2-11), 1.72 (4H, tt, *J* = 7.2, 6.8 Hz, H_2-15), 1.46–1.36 (4H, m, H_2-16); ^{13}C NMR (CD_3OD, 100 MHz) δ 169.7 (C-8), 156.7 (C-5), 133.1 (C-7a), 129.5 (C-2), 128.0 (C-3a), 113.7 (C-6/C-7), 113.6 (C-6/C-7), 110.6 (C-3), 103.9 (C-4), 56.2 (OMe), 49.8 (C-14), 46.1 (C-12), 36.5 (C-10), 28.1 (C-11), 27.3 (C-16), 27.2 (C-15); (+)-HRESIMS $[M+H]^+$ *m/z* 577.3497 (calcd for $C_{32}H_{45}N_6O_4$, 577.3497).

N[1],*N*[7]-Bis(3-(5-methoxy-1*H*-indole-3-carboxamido)propyl) heptane-1,7-diaminium 2,2,2-trifluoroacetate (**14c**)

Following general procedure A, reaction of 5-methoxy-1*H*-indole-3-carboxylic acid (0.040 g, 0.209 mmol) with EDC·HCl (0.047 g, 0.247 mmol), HOBt (0.032 g, 0.247 mmol), DIPEA (0.10 mL, 0.571 mmol) and di-*tert*-butyl heptane-1,7-diylbis((3-aminopropyl)carbamate) **11c** (0.042 g, 0.095 mmol) afforded di-*tert*-butyl heptane-1,7-diylbis((3-(5-methoxy-1*H*-indole-3-carboxamido)propyl)carbamate) (0.062 g, 82%) as a clear colorless oil. Following general procedure B, a subsample of this product (0.031 g, 0.039 mmol) was reacted with TFA in CH_2Cl_2 to afford, after chromatography, the di-TFA salt **14c** (0.010 g, 31%) as a dark yellow oil. R_f (RP-18, 10% *aq* HCl:MeOH 1:3) 0.65; IR (ATR) ν_{max} 3280, 1673, 1622, 1540, 1470, 1436, 1281, 1199, 1176, 1130, 1027, 924, 835, 880, 774, 721 cm^{-1}; ^1H NMR (CD_3OD, 400 MHz) δ 7.86 (2H, s, H-2), 7.65 (2H, d, *J* = 2.4 Hz, H-4), 7.32 (2H, d, *J* = 8.8 Hz, H-7), 6.85 (2H, dd, *J* = 8.8, 2.5 Hz, H-6), 3.82 (6H, s, OMe), 3.51 (4H, t, *J* = 6.3 Hz, H_2-10), 3.06–2.99 (8H, m, H_2-12, H_2-14), 1.98 (4H, tt, *J* = 6.6, 6.5 Hz, H_2-11), 1.80–1.72 (4H, m, H_2-15), 1.52–1.47 (6H, m, H_2-16, H_2-17); ^{13}C NMR (CD_3OD, 100 MHz) δ 169.8 (C-8), 156.7 (C-5), 133.2 (C-7a), 129.6 (C-2), 128.0 (C-3a), 113.7 (C-6/C-7), 113.6 (C-6/C-7), 110.6 (C-3), 104.0 (C-4), 56.2 (OMe), 48.7 (C-14), 46.2 (C-12), 36.5 (C-10), 28.2 (C-11), 27.12 (C-15/C-16/C-17), 27.10 (C-15/C-16/C-17); (+)-HRESIMS $[M+H]^+$ *m/z* 591.3652 (calcd for $C_{33}H_{47}N_6O_4$, 591.3653).

N[1],*N*[8]-Bis(3-(5-methoxy-1*H*-indole-3-carboxamido)propyl) octane-1,8-diaminium 2,2,2-trifluoroacetate (**14d**)

Following general procedure A, reaction of 5-methoxy-1*H*-indole-3-carboxylic acid (0.040 g, 0.209 mmol) with EDC·HCl (0.047 g, 0.247 mmol), HOBt (0.032 g, 0.247 mmol), DIPEA (0.10 mL, 0.571 mmol) and di-*tert*-butyl octane-1,8-diylbis((3-aminopropyl)carbamate) **11d** (0.044 g, 0.095 mmol) afforded di-*tert*-butyl octane-1,8-diylbis((3-(5-methoxy-1*H*-indole-3-carboxamido)propyl)carbamate) (0.054 g, 71%) as a clear colorless oil. Following general procedure B, a subsample of this product (0.027 g, 0.034 mmol) was reacted with TFA in CH_2Cl_2 to afford, after chromatography, the di-TFA salt **14d** (0.025 g, 95%) as a dark yellow oil. R_f (RP-18, 10% *aq* HCl:MeOH 1:3) 0.60; IR (ATR) ν_{max} 3260, 1673, 1622, 1540, 1471, 1435, 1281, 1199, 1175, 1130, 1028, 924, 835, 799, 774, 721 cm^{-1}; ^1H NMR (CD_3OD, 400 MHz) δ 7.88 (2H, s, H-2), 7.66 (2H, d, *J* = 2.4 Hz, H-4), 7.32 (2H, d, *J* = 8.8 Hz, H-7), 6.84 (2H, dd, *J* = 8.8, 2.5 Hz, H-6), 3.82 (6H, s, OMe), 3.51 (4H, t, *J* = 6.3 Hz, H_2-10), 3.04 (4H, t, *J* = 7.0 Hz, H_2-12), 2.97 (4H, t, *J* = 7.7 Hz, H_2-14), 1.98 (4H, tt, *J* = 6.6, 6.5 Hz, H_2-11), 1.71 (4H, tt, *J* = 6.6, 6.5 Hz, H_2-15), 1.41–1.37 (8H, m, H_2-16, H_2-17); ^{13}C NMR (CD_3OD, 100 MHz) δ 169.7 (C-8), 156.7 (C-5), 133.1 (C-7a), 129.5 (C-2), 128.0 (C-3a), 113.8 (C-6/C-7), 113.7 (C-6/C-7), 110.6 (C-3), 103.8 (C-4), 56.2 (OMe), 48.9 (C-14), 46.1 (C-12), 36.5 (C-10), 29.9 (C-17), 28.1 (C-11), 27.4 (C-16), 27.3 (C-15); (+)-HRESIMS $[M+H]^+$ *m/z* 605.3809 (calcd for $C_{34}H_{49}N_6O_4$, 605.3810).

N[1],*N*[10]-Bis(3-(5-methoxy-1*H*-indole-3-carboxamido)propyl) decane-1,10-diaminium 2,2,2-trifluoroacetate (**14e**)

Following general procedure A, reaction of 5-methoxy-1*H*-indole-3-carboxylic acid (0.040 g, 0.209 mmol) with EDC·HCl (0.047 g, 0.247 mmol), HOBt (0.032 g, 0.247 mmol), DIPEA (0.10 mL, 0.571 mmol) and di-*tert*-butyl decane-1,10-diylbis((3-aminopropyl)carbamate) **11e** (0.046 g, 0.095 mmol) afforded di-*tert*-butyl decane-1,10-diylbis((3-(5-methoxy-1*H*-indole-3-carboxamido)propyl)carbamate) (0.050 g, 63%) as a clear colorless oil. Following general procedure B, a subsample of this product (0.025 g, 0.030 mmol) was reacted with TFA in CH_2Cl_2 to afford, after chromatography, the di-TFA salt **14e** (0.020 g, 77%) as a dark brown oil. R_f (RP-18, 10% *aq* HCl:MeOH 1:3) 0.60; IR (ATR) ν_{max} 3269, 2934, 2856, 1673, 1622, 1540, 1483, 1470, 1438, 1361, 1335, 1304, 1281, 1199, 1174, 1131, 1029, 924, 835, 799, 773, 721 cm^{-1}; ^1H NMR (CD_3OD, 400 MHz) δ 7.87 (2H, s, H-2), 7.66 (2H, d, *J* = 2.4 Hz, H-4), 7.32 (2H, d, *J* = 8.8 Hz, H-7), 6.85 (2H, dd, *J* = 8.8, 2.5 Hz, H-6), 3.85 (6H, s, OMe), 3.51 (4H, t, *J* = 6.2 Hz, H_2-10), 3.05 (4H, t, *J* = 6.9 Hz, H_2-12), 2.99 (4H, t, *J* = 7.7 Hz, H_2-14), 1.98 (4H, tt, *J* = 6.6, 6.4 Hz, H_2-11), 1.72 (4H, tt, *J* = 7.6, 7.6 Hz, H_2-15), 1.44–1.32 (12H, m, H_2-16, H_2-17, H_2-18); ^{13}C NMR (CD_3OD, 100 MHz) δ 169.7 (C-8), 156.7 (C-5), 133.1 (C-7a), 129.5 (C-2), 128.0 (C-3a), 113.8 (C-6), 113.5 (C-7), 110.6 (C-3), 103.8 (C-4), 56.1 (OMe), 48.9 (C-14, obscured by solvent), 46.2 (C-12), 36.5 (C-10), 30.3 (C-18), 30.2 (C-17), 28.1 (C-11), 27.6 (C-16), 27.4 (C-15); (+)-HRESIMS $[M+H]^+$ *m/z* 633.4123 (calcd for $C_{36}H_{53}N_6O_4$, 633.4123).

N[1],*N*[12]-Bis(3-(5-methoxy-1*H*-indole-3-carboxamido)propyl) dodecane-1,12-diaminium 2,2,2-trifluoroacetate (**14f**)

Following general procedure A, reaction of 5-methoxy-1*H*-indole-3-carboxylic acid (0.040 g, 0.209 mmol) with EDC·HCl (0.047 g, 0.247 mmol), HOBt (0.032 g, 0.247 mmol), DIPEA (0.10 mL, 0.571 mmol) and di-*tert*-butyl dodecane-1,12-diylbis((3-aminopropyl)carbamate) **11f** (0.049 g, 0.095 mmol) afforded di-*tert*-butyl dodecane-1,12-diylbis((3-(5-methoxy-1*H*-indole-3-carboxamido)propyl)carbamate) (0.051 g, 62%) as a clear colorless oil. Following general procedure B, a subsample of this product (0.025 g, 0.029 mmol) was reacted with TFA in CH_2Cl_2 to afford, after chromatography, the di-TFA salt **14f** (0.022 g, 85%) as a brown-orange oil. R_f (RP-18, 10% *aq* HCl:MeOH 1:3) 0.53; IR (ATR) ν_{max} 3281, 2934, 2856, 1673, 1622, 1540, 1483, 1470, 1438, 1361, 1335, 1304, 1281, 1199, 1174, 1130, 1029, 924, 834, 799, 774, 720 cm^{-1}; ^{1}H NMR (CD_3OD, 400 MHz) δ 7.87 (2H, s, H-2), 7.66 (2H, d, *J* = 2.4 Hz, H-4), 7.32 (2H, d, *J* = 8.8 Hz, H-7), 6.85 (2H, dd, *J* = 8.9, 2.5 Hz, H-6), 3.85 (6H, s, OMe), 3.51 (4H, t, *J* = 6.3 Hz, H_2-10), 3.05 (4H, t, *J* = 6.9 Hz, H_2-12), 2.98 (4H, t, *J* = 7.7 Hz, H_2-14), 1.98 (4H, tt, *J* = 6.6, 6.5 Hz, H_2-11), 1.72 (4H, tt, *J* = 7.6, 7.5 Hz, H_2-15), 1.42–1.28 (16H, m, H_2-16, H_2-17, H_2-18, H_2-19); ^{13}C NMR (CD_3OD, 100 MHz) δ 169.7 (C-8), 156.7 (C-5), 133.1 (C-7a), 129.5 (C-2), 128.0 (C-3a), 113.8 (C-6), 113.5 (C-7), 110.6 (C-3), 103.8 (C-4), 56.1 (OMe), 48.7 (C-14, obscured by solvent), 46.2 (C-12), 36.5 (C-10), 30.3 (C-19), 30.22 (C-18), 30.16 (C-17), 28.1 (C-11), 27.6 (C-16), 27.4 (C-15); (+)-HRESIMS $[M+H]^+$ *m/z* 661.4434 (calcd for $C_{38}H_{57}N_6O_4$, 661.4436).

N[1],*N*[4]-Bis(3-(5-methyl-1*H*-indole-3-carboxamido)propyl)butane-1,4-diaminium 2,2,2-trifluoroacetate (**15a**)

Following general procedure A, reaction of 5-methyl-1*H*-indole-3-carboxylic acid (0.050 g, 0.285 mmol) with EDC·HCl (0.065 g, 0.337 mmol), HOBt (0.044 g, 0.337 mmol), DIPEA (0.14 mL, 0.778 mmol) and di-*tert*-butyl butane-1,4-diylbis((3-aminopropyl)carbamate) **11a** (0.052 g, 0.130 mmol) afforded di-*tert*-butyl butane-1,4-diylbis((3-(5-methyl-1*H*-indole-3-carboxamido)propyl)carbamate) (0.030 g, 32%) as a clear colorless oil. Following general procedure B, a subsample of this product (0.015 g, 0.021 mmol) was reacted with TFA in CH_2Cl_2 to afford, after chromatography, the di-TFA salt **15a** (0.015 g, 96%) as a pale yellow oil. R_f (RP-18, 10% *aq* HCl:MeOH 1:3) 0.62; IR (ATR) ν_{max} 3319, 1684, 1578, 1546, 1430, 1357, 1307, 1202, 1183, 1128, 919, 810, 796, 778, 723 cm^{-1}; ^{1}H NMR (CD_3OD, 400 MHz) δ 7.89 (2H, s, H-4), 7.85 (2H, s, H-2), 7.31 (2H, d, *J* = 8.3 Hz, H-7), 7.02 (2H, dd, *J* = 8.3, 1.5 Hz, H-6), 3.52 (4H, t, *J* = 6.2 Hz, H_2-10), 3.09–3.06 (8H, m, H_2-12, H_2-14), 2.42 (6H, s, Me), 1.99 (4H, tt, *J* = 6.5, 6.5 Hz, H_2-11), 1.89 (4H, tt, *J* = 3.6, 3.6 Hz, H_2-15); ^{13}C NMR (CD_3OD, 100 MHz) δ 169.8 (C-8), 136.5 (C-7a), 131.5 (C-5), 129.5 (C-2), 127.2 (C-3a), 125.2 (C-6), 121.2 (C-4), 112.6 (C-7), 110.5 (C-3), 48.0 (C-14), 46.2 (C-12), 36.5 (C-10), 28.1 (C-11), 24.4 (C-15), 21.8 (Me); (+)-HRESIMS $[M+H]^+$ *m/z* 517.3286 (calcd for $C_{30}H_{41}N_6O_2$, 517.3286).

N[1],*N*[6]-Bis(3-(5-methyl-1*H*-indole-3-carboxamido)propyl) hexane-1,6-diaminium 2,2,2-trifluoroacetate (**15b**)

Following general procedure A, reaction of 5-methyl-1*H*-indole-3-carboxylic acid (0.050 g, 0.285 mmol) with EDC·HCl (0.065 g, 0.337 mmol), HOBt (0.044 g, 0.337 mmol), DIPEA (0.14 mL, 0.778 mmol) and di-*tert*-butyl hexane-1,6-diylbis((3-aminopropyl)carbamate) **11b** (0.056 g, 0.130 mmol) afforded di-*tert*-butyl hexane-1,6-diylbis((3-(5-methyl-1*H*-indole-3-carboxamido)propyl)carbamate) (0.077 g, 80%) as a clear colorless oil. Following general procedure B, a subsample of this product (0.038 g, 0.051 mmol) was reacted with TFA in CH_2Cl_2 to afford, after chromatography, the di-TFA salt **15b** (0.005 g, 13%) as a yellow oil. R_f (RP-18, 10% *aq* HCl:MeOH 1:3) 0.55; IR (ATR) ν_{max} 3321, 1684, 1578, 1546, 1430, 1357, 1307, 1202, 1183, 1126, 919, 840, 810, 796, 778, 723 cm^{-1}; ^{1}H NMR (CD_3OD, 400 MHz) δ 7.90 (2H, s, H-4), 7.85 (2H, s, H-2), 7.32 (2H, d, *J* = 8.3 Hz, H-7), 7.03 (2H, dd, *J* = 8.3, 1.4 Hz, H-6), 3.51 (4H, t, *J* = 6.3 Hz, H_2-10), 3.04 (4H, t, *J* = 7.0 Hz, H_2-12), 3.01 (4H, t, *J* = 7.7 Hz, H_2-14), 2.43 (6H, s, Me), 1.98 (4H, tt, *J* = 6.6, 6.5 Hz, H_2-11), 1.77 (4H, tt, *J* = 7.2, 7.2 Hz, H_2-15), 1.52 (4H, tt, *J* = 3.6, 3.6 Hz, H_2-16); ^{13}C NMR (CD_3OD, 100 MHz) δ 169.8 (C-8), 136.5 (C-7a), 131.5 (C-5), 129.5 (C-2), 127.3 (C-3a), 125.2 (C-6), 121.3 (C-4), 112.6 (C-7), 110.5 (C-3), 48.7 (C-14), 46.1 (C-12), 36.5 (C-10), 28.2 (C-11), 27.13 (C-16), 27.09 (C-15), 21.8 (Me); (+)-HRESIMS $[M+H]^+$ *m/z* 545.3599 (calcd for $C_{32}H_{45}N_6O_2$, 545.3599).

N^1,N^7-Bis(3-(5-methyl-1*H*-indole-3-carboxamido)propyl) heptane-1,7-diaminium 2,2,2-trifluoroacetate (**15c**)

Following general procedure A, reaction of 5-methyl-1*H*-indole-3-carboxylic acid (0.050 g, 0.285 mmol) with EDC·HCl (0.065 g, 0.337 mmol), HOBt (0.044 g, 0.337 mmol), DIPEA (0.14 mL, 0.778 mmol) and di-*tert*-butyl heptane-1,7-diylbis((3-aminopropyl)carbamate) **11c** (0.058 g, 0.130 mmol) afforded di-*tert*-butyl heptane-1,7-diylbis((3-(5-methyl-1*H*-indole-3-carboxamido)propyl)carbamate) (0.069 g, 70%) as a clear colorless oil. Following general procedure B, a subsample of this product (0.034 g, 0.045 mmol) was reacted with TFA in CH_2Cl_2 to afford, after chromatography, the di-TFA salt **15c** (0.030 g, 85%) as a pale-yellow oil. R_f (RP-18, 10% *aq* HCl:MeOH 1:3) 0.52; IR (ATR) ν_{max} 3259, 2931, 2857, 1671, 1622, 1543, 1470, 1433, 1354, 1307, 1276, 1199, 1178, 1130, 921, 835, 799, 776, 721 cm^{-1}; ^{1}H NMR (CD_3OD, 400 MHz) δ 7.91 (2H, d, *J* = 0.9 Hz, H-4), 7.89 (2H, s, H-2), 7.31 (2H, d, *J* = 8.3 Hz, H-7), 7.02 (2H, dd, *J* = 8.4, 1.5 Hz, H-6), 3.51 (4H, t, *J* = 6.3 Hz, H_2-10), 3.03 (4H, t, *J* = 7.0 Hz, H_2-12), 2.96 (4H, t, *J* = 7.7 Hz, H_2-14), 2.42 (6H, s, Me), 1.97 (4H, tt, *J* = 6.7, 6.6 Hz, H_2-11), 1.72 (4H, tt, *J* = 7.3, 7.2 Hz, H_2-15), 1.49–1.44 (6H, m, H_2-16, H_2-17); ^{13}C NMR (CD_3OD, 100 MHz) δ 169.7 (C-8), 136.4 (C-7a), 131.5 (C-5), 129.4 (C-2), 127.3 (C-3a), 125.2 (C-6), 121.3 (C-4), 112.6 (C-7), 110.5 (C-3), 48.3 (C-14, obscured by solvent), 46.1 (C-12), 36.5 (C-10), 29.6 (C-17), 28.1 (C-11), 27.3 (C-16), 27.1 (C-15), 21.8 (Me); (+)-HRESIMS $[M+H]^+$ *m/z* 559.3754 (calcd for $C_{33}H_{47}N_6O_2$, 559.3755).

N^1,N^8-Bis(3-(5-methyl-1*H*-indole-3-carboxamido)propyl) octane-1,8-diaminium 2,2,2-trifluoroacetate (**15d**)

Following general procedure A, reaction of 5-methyl-1*H*-indole-3-carboxylic acid (0.050 g, 0.285 mmol) with EDC·HCl (0.065 g, 0.337 mmol), HOBt (0.044 g, 0.337 mmol), DIPEA (0.14 mL, 0.778 mmol) and di-*tert*-butyl octane-1,8-diylbis((3-aminopropyl)carbamate) **11d** (0.060 g, 0.130 mmol) afforded di-*tert*-butyl octane-1,8-diylbis((3-(5-methyl-1*H*-indole-3-carboxamido)propyl)carbamate) (0.068 g, 68%) as a clear colorless oil. Following general procedure B, a subsample of this product (0.039 g, 0.051 mmol) was reacted with TFA in CH_2Cl_2 to afford, after chromatography, the di-TFA salt **15d** (0.037 g, 92%) as a yellow oil. R_f (RP-18, 10% *aq* HCl:MeOH 1:3) 0.50; IR (ATR) ν_{max} 3259, 2930, 2857, 1671, 1622, 1541, 1469, 1432, 1354, 1328, 1307, 1276, 1199, 1178, 1130, 921, 835, 799, 777, 721 cm^{-1}; ^{1}H NMR (CD_3OD, 400 MHz) δ 7.91 (2H, br s, H-4), 7.87 (2H, s, H-2), 7.31 (2H, d, *J* = 8.4 Hz, H-7), 7.02 (2H, dd, *J* = 8.4, 1.5 Hz, H-6), 3.51 (4H, t, *J* = 6.3 Hz, H_2-10), 3.03 (4H, t, *J* = 7.0 Hz, H_2-12), 2.96 (4H, t, *J* = 7.7 Hz, H_2-14), 2.43 (6H, s, Me), 1.97 (4H, tt, *J* = 6.6, 6.5 Hz, H_2-11), 1.71 (4H, tt, *J* = 7.5, 7.5 Hz, H_2-15), 1.46–1.36 (8H, m, H_2-16, H_2-17); ^{13}C NMR (CD_3OD, 100 MHz) δ 169.7 (C-8), 136.4 (C-7a), 131.5 (C-5), 129.4 (C-2), 127.3 (C-3a), 125.2 (C-6), 121.3 (C-4), 112.6 (C-7), 110.5 (C-3), 48.9 (C-14), 46.1 (C-12), 36.5 (C-10), 29.9 (C-17), 28.1 (C-11), 27.4 (C-16), 27.3 (C-15), 21.8 (Me); (+)-HRESIMS $[M+H]^+$ *m/z* 573.3912 (calcd for $C_{34}H_{49}N_6O_2$, 573.3912).

N^1,N^{10}-Bis(3-(5-methyl-1*H*-indole-3-carboxamido)propyl) decane-1,10-diaminium 2,2,2-trifluoroacetate (**15e**)

Following general procedure A, reaction of 5-methyl-1*H*-indole-3-carboxylic acid (0.050 g, 0.285 mmol) with EDC·HCl (0.065 g, 0.337 mmol), HOBt (0.044 g, 0.337 mmol), DIPEA (0.14 mL, 0.778 mmol) and di-*tert*-butyl decane-1,10-diylbis((3-aminopropyl)carbamate) **11e** (0.063 g, 0.130 mmol) afforded di-*tert*-butyl decane-1,10-diylbis((3-(5-methyl-1*H*-indole-3-carboxamido)propyl)carbamate) (0.075 g, 72%) as a clear colorless oil. Following general procedure B, a subsample of this product (0.037 g, 0.046 mmol) was reacted with TFA in CH_2Cl_2 to afford, after chromatography, the di-TFA salt **15e** (0.037 g, 97%) as a brown oil. R_f (RP-18, 10% *aq* HCl:MeOH 1:3) 0.40; IR (ATR) ν_{max} 3260, 2931, 2857, 1671, 1622, 1542, 1469, 1432, 1353, 1328, 1307, 1276, 1199, 1178, 1129, 921, 835, 799, 777, 720 cm^{-1}; ^{1}H NMR (CD_3OD, 400 MHz) δ 7.92 (2H, br s, H-4), 7.87 (2H, s, H-2), 7.31 (2H, d, *J* = 8.4 Hz, H-7), 7.02 (2H, dd, *J* = 8.3, 1.5 Hz, H-6), 3.51 (4H, t, *J* = 6.3 Hz, H_2-10), 3.03 (4H, t, *J* = 7.0 Hz, H_2-12), 2.97 (4H, t, *J* = 7.8 Hz, H_2-14), 2.43 (6H, s, Me), 1.98 (4H, tt, *J* = 6.6, 6.5 Hz, H_2-11), 1.71 (4H, tt, *J* = 7.6, 7.5 Hz, H_2-15), 1.43–1.33 (12H, m, H_2-16, H_2-17, H_2-18); ^{13}C NMR (CD_3OD, 100 MHz) δ 169.7 (C-8), 136.4 (C-7a), 131.5 (C-5), 129.4 (C-2), 127.3 (C-3a), 125.2 (C-6), 121.3 (C-4), 112.6 (C-7), 110.5 (C-3), 48.7 (C-14, obscured by solvent), 46.1 (C-12), 36.5 (C-10), 30.3 (C-18), 30.1 (C-17), 28.1 (C-11), 27.5 (C-16), 27.3 (C-15), 21.8 (Me); (+)-HRESIMS $[M+H]^+$ *m/z* 601.4224 (calcd for $C_{36}H_{53}N_6O_2$, 601.4225).

N[1],*N*[12]-Bis(3-(5-methyl-1*H*-indole-3-carboxamido)propyl) dodecane-1,12-diaminium 2,2,2-trifluoroacetate (**15f**)

Following general procedure A, reaction of 5-methyl-1*H*-indole-3-carboxylic acid (0.050 g, 0.285 mmol) with EDC·HCl (0.065 g, 0.337 mmol), HOBt (0.044 g, 0.337 mmol), DIPEA (0.14 mL, 0.778 mmol) and di-*tert*-butyl dodecane-1,12-diylbis((3-aminopropyl)carbamate) **11f** (0.067 g, 0.130 mmol) afforded di-*tert*-butyl dodecane-1,12-diylbis((3-(5-methyl-1*H*-indole-3-carboxamido)propyl)carbamate) (0.023 g, 21%) as a clear colorless oil. Following general procedure B, a subsample of this product (0.011 g, 0.013 mmol) was reacted with TFA in CH_2Cl_2 to afford, after chromatography, the di-TFA salt **15f** (0.007 g, 62%) as an orange oil. R_f (RP-18, 10% *aq* HCl:MeOH 1:3) 0.30; IR (ATR) ν_{max} 3265, 2930, 2857, 1671, 1622, 1542, 1468, 1432, 1353, 1328, 1307, 1276, 1199, 1177, 1129, 921, 835, 799, 776, 720 cm^{-1}; ^1H NMR (CD_3OD, 400 MHz) δ 7.91 (2H, br s, H-4), 7.86 (2H, s, H-2), 7.32 (2H, d, *J* = 8.3 Hz, H-7), 7.03 (2H, d, *J* = 8.4 Hz, H-6), 3.51 (4H, t, *J* = 6.3 Hz, H_2-10), 3.05 (4H, t, *J* = 6.8 Hz, H_2-12), 2.99 (4H, t, *J* = 7.7 Hz, H_2-14), 2.43 (6H, s, Me), 1.98 (4H, tt, *J* = 6.6, 6.5 Hz, H_2-11), 1.73 (4H, tt, *J* = 7.6, 7.6 Hz, H_2-15), 1.45–1.31 (16H, m, H_2-16, H_2-17, H_2-18, H_2-19); ^{13}C NMR (CD_3OD, 100 MHz) δ 169.8 (C-8), 136.5 (C-7a), 131.5 (C-5), 129.4 (C-2), 127.4 (C-3a), 125.2 (C-6), 121.3 (C-4), 112.6 (C-7), 110.5 (C-3), 48.7 (C-14, obscured by solvent), 46.1 (C-12), 36.5 (C-10), 30.6 (C-19), 30.5 (C-18), 30.2 (C-17), 28.2 (C-11), 27.6 (C-16), 27.4 (C-15), 21.8 (Me); (+)-HRESIMS $[M+H]^+$ *m/z* 629.4543 (calcd for $C_{38}H_{57}N_6O_2$, 629.4538).

N[1],*N*[4]-Bis(3-(7-fluoro-1*H*-indole-3-carboxamido)propyl)butane-1,4-diaminium 2,2,2-trifluoroacetate (**16a**)

Following general procedure A, reaction of 7-fluoro-1*H*-indole-3-carboxylic acid (0.040 g, 0.223 mmol) with EDC·HCl (0.051 g, 0.264 mmol), HOBt (0.034 g, 0.264 mmol), DIPEA (0.11 mL, 0.609 mmol) and di-*tert*-butyl butane-1,4-diylbis((3-aminopropyl)carbamate) **11a** (0.041 g, 0.101 mmol) afforded di-*tert*-butyl butane-1,4-diylbis((3-(7-fluoro-1*H*-indole-3-carboxamido)propyl)carbamate) (0.050 g, 68%) as a clear colorless oil. Following general procedure B, a subsample of this product (0.017 g, 0.024 mmol) was reacted with TFA in CH_2Cl_2 to afford, after chromatography, the di-TFA salt **16a** (0.016 g, 91%) as a red oil. R_f (RP-18, 10% *aq* HCl:MeOH 1:3) 0.60; IR (ATR) ν_{max} 3205, 2866, 1671, 1622, 1582, 1548, 1505, 1440, 1312, 1241, 1199, 1182, 1129, 1045, 961, 836, 792, 721 cm^{-1}; ^1H NMR (CD_3OD, 400 MHz) δ 7.93 (2H, s, H-2), 7.89 (2H, d, *J* = 8.2 Hz, H-4), 7.09 (2H, td, *J* = 7.9, 4.8 Hz, H-5), 6.92 (4H, dd, *J* = 11.2, 7.8 Hz, H-6), 3.52 (4H, t, *J* = 6.3 Hz, H_2-10), 3.11–3.07 (8H, m, H_2-12, H_2-14), 2.00 (4H, tt, *J* = 6.8, 6.5 Hz, H_2-11), 1.89 (4H, tt, *J* = 3.6, 3.5 Hz, H_2-15); ^{13}C NMR (CD_3OD, 100 MHz) δ 169.2 (C-8), 151.1 (d, $^1J_{CF}$ = 244.2 Hz, C-7), 130.8 (d, $^3J_{CF}$ = 4.8 Hz, C-3a), 130.1 (C-2), 126.2 (d, $^2J_{CF}$ = 13.6 Hz, C-7a), 122.5 (d, $^3J_{CF}$ = 6.1 Hz, C-5), 117.8 (d, $^4J_{CF}$ = 3.5 Hz, C-4), 108.2 (d, $^2J_{CF}$ = 16.0 Hz, C-6), 48.1 (C-14), 46.2 (C-12), 36.6 (C-10), 28.1 (C-11), 24.4 (C-15); (+)-HRESIMS $[M+H]^+$ *m/z* 525.2783 (calcd for $C_{28}H_{35}F_2N_6O_2$, 525.2784).

N[1],*N*[6]-Bis(3-(7-fluoro-1*H*-indole-3-carboxamido)propyl) hexane-1,6-diaminium 2,2,2-trifluoroacetate (**16b**)

Following general procedure A, reaction of 7-fluoro-1*H*-indole-3-carboxylic acid (0.040 g, 0.223 mmol) with EDC·HCl (0.051 g, 0.264 mmol), HOBt (0.034 g, 0.264 mmol), DIPEA (0.11 mL, 0.609 mmol) and di-*tert*-butyl hexane-1,6-diylbis((3-aminopropyl)carbamate) **11b** (0.044 g, 0.101 mmol) afforded di-*tert*-butyl hexane-1,6-diylbis((3-(7-fluoro-1*H*-indole-3-carboxamido)propyl)carbamate) (0.021 g, 27%) as a clear colorless oil. Following general procedure B, a subsample of this product (0.018 g, 0.024 mmol) was reacted with TFA in CH_2Cl_2 to afford, after chromatography, the di-TFA salt **16b** (0.016 g, 86%) as a red oil. R_f (RP-18, 10% *aq* HCl:MeOH 1:3) 0.57; IR (ATR) ν_{max} 3197, 2868, 1671, 1622, 1582, 1548, 1505, 1437, 1312, 1241, 1199, 1181, 1129, 1045, 962, 835, 792, 721 cm^{-1}; ^1H NMR (CD_3OD, 400 MHz) δ 7.94 (2H, s, H-2), 7.90 (2H, d, *J* = 8.0 Hz, H-4), 7.09 (2H, td, *J* = 8.0, 4.9 Hz, H-5), 6.92 (4H, dd, *J* = 11.2, 7.7 Hz, H-6), 3.52 (4H, t, *J* = 6.3 Hz, H_2-10), 3.06 (4H, t, *J* = 7.8 Hz, H_2-12), 3.02 (4H, t, *J* = 7.8 Hz, H_2-14), 1.99 (4H, tt, *J* = 6.7, 6.6 Hz, H_2-11), 1.77 (4H, tt, *J* = 7.4, 7.3 Hz, H_2-15), 1.50 (4H, tt, *J* = 3.6, 3.6 Hz, H_2-16); ^{13}C NMR (CD_3OD, 100 MHz) δ 169.1 (C-8), 151.1 (d, $^1J_{CF}$ = 244.2 Hz, C-7), 130.9 (d, $^3J_{CF}$ = 5.0 Hz, C-3a), 130.0 (C-2), 126.2 (d, $^2J_{CF}$ = 13.9 Hz, C-7a), 122.5 (d, $^3J_{CF}$ = 6.1 Hz, C-5), 117.8 (d, $^4J_{CF}$ = 3.4 Hz, C-4), 108.2 (d, $^2J_{CF}$ = 16.0

Hz, C-6), 48.6 (C-14, obscured by solvent), 46.2 (C-12), 36.6 (C-10), 28.1 (C-11), 27.11 (C-16), 27.06 (C-15); (+)-HRESIMS $[M+H]^+$ *m/z* 553.3097 (calcd for $C_{30}H_{39}F_2N_6O_2$, 553.3097).

N[1],*N*[7]-Bis(3-(7-fluoro-1*H*-indole-3-carboxamido)propyl) heptane-1,7-diaminium 2,2,2-trifluoroacetate (**16c**)

Following general procedure A, reaction of 7-fluoro-1*H*-indole-3-carboxylic acid (0.040 g, 0.223 mmol) with EDC·HCl (0.051 g, 0.264 mmol), HOBt (0.034 g, 0.264 mmol), DIPEA (0.11 mL, 0.609 mmol) and di-*tert*-butyl heptane-1,7-diylbis((3-aminopropyl)carbamate) **11c** (0.045 g, 0.101 mmol) afforded di-*tert*-butyl heptane-1,7-diylbis((3-(7-fluoro-1*H*-indole-3-carboxamido)propyl)carbamate) (0.062 g, 80%) as a clear colorless oil. Following general procedure B, a subsample of this product (0.031 g, 0.040 mmol) was reacted with TFA in CH_2Cl_2 to afford, after chromatography, the di-TFA salt **16c** (0.017 g, 53%) as a pale-yellow oil. R_f (RP-18, 10% *aq* HCl:MeOH 1:3) 0.55; IR (ATR) ν_{max} 3027, 2865, 1671, 1622, 1582, 1548, 1505, 1437, 1312, 1241, 1199, 1180, 1129, 1045, 961, 836, 792, 721 cm^{-1}; ^1H NMR (CD_3OD, 400 MHz) δ 7.95 (2H, s, H-2), 7.91 (2H, d, *J* = 8.0 Hz, H-4), 7.09 (2H, td, *J* = 8.0, 4.9 Hz, H-5), 6.92 (4H, dd, *J* = 11.2, 7.9 Hz, H-6), 3.52 (4H, t, *J* = 6.3 Hz, H_2-10), 3.06 (4H, t, *J* = 7.0 Hz, H_2-12), 3.00 (4H, t, *J* = 7.7 Hz, H_2-14), 1.99 (4H, tt, *J* = 6.6, 6.6 Hz, H_2-11), 1.73 (4H, tt, *J* = 7.1, 6.7 Hz, H_2-15), 1.48–1.41 (6H, m, H_2-16, H_2-17); ^{13}C NMR (CD_3OD, 100 MHz) δ 169.0 (C-8), 151.1 (d, $^1J_{CF}$ = 244.3 Hz, C-7), 130.9 (d, $^3J_{CF}$ = 4.9 Hz, C-3a), 130.0 (C-2), 126.2 (d, $^2J_{CF}$ = 13.8 Hz, C-7a), 122.5 (d, $^3J_{CF}$ = 6.1 Hz, C-5), 117.8 (d, $^4J_{CF}$ = 3.5 Hz, C-4), 108.2 (d, $^2J_{CF}$ = 15.9 Hz, C-6), 48.9 (C-14), 46.2 (C-12), 36.6 (C-10), 29.6 (C-17), 28.1 (C-11), 27.3 (C-16), 27.2 (C-15); (+)-HRESIMS $[M+H]^+$ *m/z* 567.3253 (calcd for $C_{31}H_{41}F_2N_6O_2$, 567.3254).

N[1],*N*[8]-Bis(3-(7-fluoro-1*H*-indole-3-carboxamido)propyl) octane-1,8-diaminium 2,2,2-trifluoroacetate (**16d**)

Following general procedure A, reaction of 7-fluoro-1*H*-indole-3-carboxylic acid (0.040 g, 0.223 mmol) with EDC·HCl (0.051 g, 0.264 mmol), HOBt (0.034 g, 0.264 mmol), DIPEA (0.11 mL, 0.609 mmol) and di-*tert*-butyl octane-1,8-diylbis((3-aminopropyl)carbamate) **11d** (0.047 g, 0.101 mmol) afforded di-*tert*-butyl octane-1,8-diylbis((3-(7-fluoro-1*H*-indole-3-carboxamido)propyl)carbamate) (0.076 g, 96%) as a clear colorless oil. Following general procedure B, a subsample of this product (0.040 g, 0.051 mmol) was reacted with TFA in CH_2Cl_2 to afford, after chromatography, the di-TFA salt **16d** (0.035 g, 84%) as a yellow oil. R_f (RP-18, 10% *aq* HCl:MeOH 1:3) 0.52; IR (ATR) ν_{max} 3353, 3009, 2863, 1666, 1609, 1557, 1530, 1497, 1470, 1433, 1400, 1346, 1311, 1298, 1237, 1192, 1116, 1043, 955, 864, 837, 794, 719 cm^{-1}; ^1H NMR (CD_3OD, 400 MHz) δ 7.95 (2H, s, H-2), 7.91 (2H, d, *J* = 8.0 Hz, H-4), 7.09 (2H, td, *J* = 8.0, 4.7 Hz, H-5), 6.92 (4H, dd, *J* = 11.5, 7.9 Hz, H-6), 3.52 (4H, t, *J* = 6.3 Hz, H_2-10), 3.06 (4H, t, *J* = 7.0 Hz, H_2-12), 2.99 (4H, t, *J* = 7.7 Hz, H_2-14), 1.99 (4H, tt, *J* = 6.7, 6.6 Hz, H_2-11), 1.72 (4H, tt, *J* = 7.4, 7.4 Hz, H_2-15), 1.43–1.39 (8H, m, H_2-16, H_2-17); ^{13}C NMR (CD_3OD, 100 MHz) δ 169.0 (C-8), 151.1 (d, $^1J_{CF}$ = 244.2 Hz, C-7), 130.9 (d, $^3J_{CF}$ = 4.8 Hz, C-3a), 130.0 (C-2), 126.2 (d, $^2J_{CF}$ = 13.8 Hz, C-7a), 122.4 (d, $^3J_{CF}$ = 6.2 Hz, C-5), 117.8 (d, $^4J_{CF}$ = 3.4 Hz, C-4), 108.2 (d, $^2J_{CF}$ = 15.9 Hz, C-6), 48.9 (C-14), 46.2 (C-12), 36.6 (C-10), 29.9 (C-17), 28.0 (C-11), 27.4 (C-16), 27.3 (C-15); (+)-HRESIMS $[M+H]^+$ *m/z* 581.3414 (calcd for $C_{32}H_{43}F_2N_6O_2$, 581.3410).

N[1],*N*[10]-Bis(3-(7-fluoro-1*H*-indole-3-carboxamido)propyl) decane-1,10-diaminium 2,2,2-trifluoroacetate (**16e**)

Following general procedure A, reaction of 7-fluoro-1*H*-indole-3-carboxylic acid (0.040 g, 0.223 mmol) with EDC·HCl (0.051 g, 0.264 mmol), HOBt (0.034 g, 0.264 mmol), DIPEA (0.11 mL, 0.609 mmol) and di-*tert*-butyl decane-1,10-diylbis((3-aminopropyl)carbamate) **11e** (0.049 g, 0.101 mmol) afforded di-*tert*-butyl decane-1,10-diylbis((3-(7-fluoro-1*H*-indole-3-carboxamido)propyl)carbamate) (0.024 g, 29%) as a clear colorless oil. Following general procedure B, a subsample of this product (0.012 g, 0.015 mmol) was reacted with TFA in CH_2Cl_2 to afford, after chromatography, the di-TFA salt **16e** (0.005 g, 40%) as a brown oil. R_f (RP-18, 10% *aq* HCl:MeOH 1:3) 0.47; IR (ATR) ν_{max} 3354, 3013, 2863, 1667, 1609, 1557, 1530, 1498, 1470, 1433, 1311, 1237, 1192, 1116, 1043, 970, 846, 794, 719 cm^{-1}; ^1H NMR (CD_3OD, 400 MHz) δ 7.94 (2H, s, H-2), 7.91 (2H, d, *J* = 8.0 Hz, H-4), 7.10 (2H, td, *J* = 7.9, 4.8 Hz, H-5), 6.93 (4H, dd, *J* = 11.2, 7.8 Hz, H-6), 3.52 (4H, t, *J* = 6.3 Hz, H_2-10), 3.06 (4H, t, *J* = 6.9 Hz, H_2-12), 3.01 (4H, t, *J* = 7.7 Hz, H_2-14), 1.99 (4H, tt, *J* = 6.5, 6.5 Hz, H_2-11), 1.73 (4H, tt, *J* = 7.6, 7.6 Hz, H_2-15), 1.45–1.36 (12H, m, H_2-16, H_2-17,

H_2-18); ^{13}C NMR (CD_3OD, 100 MHz) δ 169.1 (C-8), 151.1 (d, $^1J_{CF}$ = 244.3 Hz, C-7), 130.9 (d, $^3J_{CF}$ = 5.2 Hz, C-3a), 130.0 (C-2), 126.2 (d, $^2J_{CF}$ = 13.9 Hz, C-7a), 122.5 (d, $^3J_{CF}$ = 6.1 Hz, C-5), 117.9 (d, $^4J_{CF}$ = 3.4 Hz, C-4), 108.2 (d, $^2J_{CF}$ = 16.1 Hz, C-6), 48.8 (C-14, obscured by solvent), 46.2 (C-12), 36.6 (C-10), 30.4 (C-18), 30.2 (C-17), 28.1 (C-11), 27.5 (C-16), 27.4 (C-15); (+)-HRESIMS $[M+H]^+$ *m/z* 609.3721 (calcd for $C_{34}H_{47}F_2N_6O_2$, 609.3723).

N^1,*N*^{12}-Bis(3-(7-fluoro-1*H*-indole-3-carboxamido)propyl) dodecane-1,12-diaminium 2,2,2-trifluoroacetate (**16f**)

Following general procedure A, reaction of 7-fluoro-1*H*-indole-3-carboxylic acid (0.040 g, 0.223 mmol) with EDC·HCl (0.051 g, 0.264 mmol), HOBt (0.034 g, 0.264 mmol), DIPEA (0.11 mL, 0.609 mmol) and di-*tert*-butyl dodecane-1,12-diylbis((3-aminopropyl)carbamate) **11f** (0.052 g, 0.101 mmol) afforded di-*tert*-butyl dodecane-1,12-diylbis((3-(7-fluoro-1*H*-indole-3-carboxamido)propyl)carbamate) (0.018 g, 21%) as a clear colorless oil. Following general procedure B, a subsample of this product (0.009 g, 0.011 mmol) was reacted with TFA in CH_2Cl_2 to afford, after chromatography, the di-TFA salt **16f** (0.003 g, 32%) as a yellow oil. R_f (RP-18, 10% *aq* HCl:MeOH 1:3) 0.45; IR (ATR) ν_{max} 3354, 3010, 1666, 1609, 1557, 1521, 1498, 1470, 1433, 1311, 1237, 1192, 1116, 1042, 955, 837, 794, 719 cm^{-1}; ^1H NMR (CD_3OD, 400 MHz) δ 7.94 (2H, s, H-2), 7.91 (2H, d, *J* = 8.2 Hz, H-4), 7.13–7.07 (2H, m, H-5), 6.97–6.91 (4H, m, H-6), 3.53 (4H, t, *J* = 6.3 Hz, H_2-10), 3.06 (4H, t, *J* = 6.9 Hz, H_2-12), 3.01 (4H, t, *J* = 7.7 Hz, H_2-14), 2.02–1.95 (4H, m, H_2-11), 1.77–1.70 (4H, m, H_2-15), 1.47–1.29 (16H, m, H_2-16, H_2-17, H_2-18, H_2-19); ^{13}C NMR (CD_3OD, 100 MHz) δ 169.1 (C-8), 151.1 (d, $^1J_{CF}$ = 244.0 Hz, C-7), 130.9 (d, $^3J_{CF}$ = 5.1 Hz, C-3a), 130.0 (C-2), 126.2 (d, $^2J_{CF}$ = 13.9 Hz, C-7a), 122.5 (d, $^3J_{CF}$ = 6.5 Hz, C-5), 117.9 (d, $^4J_{CF}$ = 3.7 Hz, C-4), 108.2 (d, $^2J_{CF}$ = 15.9 Hz, C-6), 48.8 (C-14, obscured by solvent), 46.2 (C-12), 36.6 (C-10), 30.5 (C-19), 30.4 (C-18), 30.2 (C-17), 28.2 (C-11), 27.6 (C-16), 27.4 (C-15); (+)-HRESIMS $[M+H]^+$ *m/z* 637.4036 (calcd for $C_{36}H_{51}F_2N_6O_2$, 637.4036).

N^1,*N*^{4}-Bis(3-(7-methoxy-1*H*-indole-3-carboxamido)propyl)butane-1,4-diaminium 2,2,2-trifluoroacetate (**17a**)

Following general procedure A, reaction of 7-methoxy-1*H*-indole-3-carboxylic acid (0.040 g, 0.209 mmol) with EDC·HCl (0.047 g, 0.247 mmol), HOBt (0.032 g, 0.247 mmol), DIPEA (0.10 mL, 0.571 mmol) and di-*tert*-butyl butane-1,4-diylbis((3-aminopropyl)carbamate) **11a** (0.038 g, 0.095 mmol) afforded di-*tert*-butyl butane-1,4-diylbis((3-(7-methoxy-1*H*-indole-3-carboxamido)propyl)carbamate) (0.017 g, 24%) as a clear colorless oil. Following general procedure B, this product (0.017 g, 0.023 mmol) was reacted with TFA in CH_2Cl_2 to afford, after chromatography, the di-TFA salt **17a** (0.007 g, 40%) as a yellow oil. R_f (RP-18, 10% *aq* HCl:MeOH 1:3) 0.63; IR (ATR) ν_{max} 3337, 2849, 1671, 1627, 1546, 1501, 1455, 1433, 1291, 1257, 1200, 1184, 1131, 1068, 1033, 836, 791, 721 cm^{-1}; ^1H NMR (CD_3OD, 400 MHz) δ 7.84 (2H, s, H-2), 7.66 (2H, dd, *J* = 8.0, 1.1 Hz, H-4), 7.07 (2H, dd, *J* = 8.0, 7.8 Hz, H-5), 6.69 (2H, d, *J* = 7.8 Hz, H-6), 3.94 (6H, s, OMe), 3.51 (4H, t, *J* = 6.3 Hz, H_2-10), 3.08–3.05 (8H, m, H_2-12, H_2-14), 1.98 (4H, tt, *J* = 6.6, 6.5 Hz, H_2-11), 1.88 (4H, tt, *J* = 3.6, 3.6 Hz, H_2-15); ^{13}C NMR (CD_3OD, 100 MHz) δ 169.8 (C-8), 148.1 (C-7), 128.8 (C-2), 128.5 (C-3a/C-7a), 128.4 (C-3a/C-7a), 122.9 (C-5), 114.2 (C-4), 111.4 (C-3), 103.2 (C-6), 55.9 (OMe), 48.1 (C-14), 46.2 (C-12), 36.6 (C-10), 28.1 (C-11), 24.4 (C-15); (+)-HRESIMS $[M+H]^+$ *m/z* 549.3181 (calcd for $C_{30}H_{41}N_6O_4$, 549.3184).

N^1,*N*^{6}-Bis(3-(7-methoxy-1*H*-indole-3-carboxamido)propyl) hexane-1,6-diaminium 2,2,2-trifluoroacetate (**17b**)

Following general procedure A, reaction of 7-methoxy-1*H*-indole-3-carboxylic acid (0.040 g, 0.209 mmol) with EDC·HCl (0.047 g, 0.247 mmol), HOBt (0.032 g, 0.247 mmol), DIPEA (0.10 mL, 0.571 mmol) and di-*tert*-butyl hexane-1,6-diylbis((3-aminopropyl)carbamate) **11b** (0.041 g, 0.095 mmol) afforded di-*tert*-butyl hexane-1,6-diylbis((3-(7-methoxy-1*H*-indole-3-carboxamido)propyl)carbamate) (0.014, 19%) as a clear colorless oil. Following general procedure B, a subsample of this product (0.015 g, 0.019 mmol) was reacted with TFA in CH_2Cl_2 to afford, after chromatography, the di-TFA salt **17b** (0.007 g, 45%) as a yellow oil. R_f (RP-18, 10% *aq* HCl:MeOH 1:3) 0.62; IR (ATR) ν_{max} 3355, 2848, 1671, 1527, 1546, 1501, 1455, 1433, 1313, 1281, 1267, 1200, 1184, 1130, 1068, 1033, 836, 791, 721 cm^{-1}; ^1H NMR (CD_3OD, 400 MHz) δ 7.84 (2H, s, H-2), 7.67 (2H, d, *J* = 8.3 Hz, H-4), 7.08 (2H, dd, *J* = 8.3, 7.8 Hz, H-5), 6.70 (2H, d, *J* = 7.8 Hz,

H-6), 3.95 (6H, s, OMe), 3.51 (4H, t, J = 6.3 Hz, H_2-10), 3.04 (4H, t, J = 6.4 Hz, H_2-12), 3.00 (4H, t, J = 7.5 Hz, H_2-14), 1.97 (4H, tt, J = 6.6, 6.5 Hz, H_2-11), 1.76 (4H, tt, J = 7.1, 7.1 Hz, H_2-15), 1.51 (4H, tt, J = 5.5, 3.8 Hz, H_2-16); ^{13}C NMR (CD_3OD, 100 MHz) δ 169.7 (C-8), 148.0 (C-7), 128.8 (C-2), 128.5 (C-3a/C-7a), 128.4 (C-3a/C-7a), 122.9 (C-5), 114.2 (C-4), 111.4 (C-3), 103.6 (C-6), 55.9 (OMe), 48.7 (C-14), 46.1 (C-12), 36.5 (C-10), 28.1 (C-11), 27.1 (C-16), 27.0 (C-15); (+)-HRESIMS $[M+H]^+$ *m/z* 577.3495 (calcd for $C_{32}H_{45}N_6O_4$, 577.3497).

N[1],*N*[7]-Bis(3-(7-methoxy-1*H*-indole-3-carboxamido)propyl) heptane-1,7-diaminium 2,2,2-trifluoroacetate (**17c**)

Following general procedure A, reaction of 7-methoxy-1*H*-indole-3-carboxylic acid (0.040 g, 0.209 mmol) with EDC·HCl (0.047 g, 0.247 mmol), HOBt (0.032 g, 0.247 mmol), DIPEA (0.10 mL, 0.571 mmol) and di-*tert*-butyl heptane-1,7-diylbis((3-aminopropyl)carbamate) **11c** (0.042 g, 0.095 mmol) afforded di-*tert*-butyl heptane-1,7-diylbis((3-(7-methoxy-1*H*-indole-3-carboxamido)propyl)carbamate) (0.015 g, 20%) as a clear colorless oil. Following general procedure B, a subsample of this product (0.010 g, 0.013 mmol) was reacted with TFA in CH_2Cl_2 to afford, after chromatography, the di-TFA salt **17c** (0.005 g, 48%) as a yellow oil. R_f (RP-18, 10% *aq* HCl:MeOH 1:3) 0.60; IR (ATR) ν_{max} 3318, 2846, 1671, 1627, 1546, 1501, 1455, 1433, 1313, 1281, 1267, 1200, 1184, 1130, 1068, 1033, 836, 791, 721 cm^{-1}; ^{1}H NMR (CD_3OD, 400 MHz) δ 7.85 (2H, s, H-2), 7.67 (2H, dd, J = 8.2, 0.9 Hz, H-4), 7.08 (2H, dd, J = 8.2, 7.5 Hz, H-5), 6.71 (2H, d, J = 7.5 Hz, H-6), 3.95 (6H, s, OMe), 3.51 (4H, t, J = 6.3 Hz, H_2-10), 3.04 (4H, t, J = 6.9 Hz, H_2-12), 2.99 (4H, t, J = 7.7 Hz, H_2-14), 1.97 (4H, tt, J = 6.6, 6.5 Hz, H_2-11), 1.74 (4H, tt, J = 7.4, 7.2 Hz, H_2-15), 1.49–1.45 (6H, m, H_2-16, H_2-17); ^{13}C NMR (CD_3OD, 100 MHz) δ 169.7 (C-8), 148.1 (C-7), 128.8 (C-2), 128.6 (C-3a/C-7a), 128.4 (C-3a/C-7a), 122.9 (C-5), 114.3 (C-4), 111.5 (C-3), 103.6 (C-6), 55.9 (OMe), 48.9 (C-14), 46.1 (C-12), 36.5 (C-10), 29.7 (C-17), 28.1 (C-11), 27.3 (C-16), 27.2 (C-15); (+)-HRESIMS $[M+H]^+$ *m/z* 591.3655 (calcd for $C_{33}H_{47}N_6O_4$, 591.3655).

N[1],*N*[8]-Bis(3-(7-methoxy-1*H*-indole-3-carboxamido)propyl) octane-1,8-diaminium 2,2,2-trifluoroacetate (**17d**)

Following general procedure A, reaction of 7-methoxy-1*H*-indole-3-carboxylic acid (0.040 g, 0.209 mmol) with EDC·HCl (0.047 g, 0.247 mmol), HOBt (0.032 g, 0.247 mmol), DIPEA (0.10 mL, 0.571 mmol) and di-*tert*-butyl octane-1,8-diylbis((3-aminopropyl)carbamate) **11d** (0.044 g, 0.095 mmol) afforded di-*tert*-butyl octane-1,8-diylbis((3-(7-methoxy-1*H*-indole-3-carboxamido)propyl)carbamate) (0.028 g, 37%) as a clear colorless oil. Following general procedure B, a subsample of this product (0.043 g, 0.053 mmol) was reacted with TFA in CH_2Cl_2 to afford, after chromatography, the di-TFA salt **17d** (0.012 g, 27%) as a yellow oil. R_f (RP-18, 10% *aq* HCl:MeOH 1:3) 0.53; IR (ATR) ν_{max} 3322, 2846, 1671, 1627, 1546, 1501, 1455, 1433, 1313, 1281, 1257, 1200, 1184, 1130, 1068, 1033, 836, 791, 721 cm^{-1}; ^{1}H NMR (CD_3OD, 400 MHz) δ 7.85 (2H, s, H-2), 7.67 (2H, d, J = 8.5 Hz, H-4), 7.07 (2H, dd, J = 8.5, 7.6 Hz, H-5), 6.71 (2H, d, J = 7.6 Hz, H-6), 3.94 (6H, s, OMe), 3.51 (4H, t, J = 6.3 Hz, H_2-10), 3.04 (4H, t, J = 6.9 Hz, H_2-12), 2.98 (4H, t, J = 7.6 Hz, H_2-14), 1.98 (4H, tt, J = 6.5, 6.5 Hz, H_2-11), 1.72 (4H, tt, J = 7.4, 7.3 Hz, H_2-15), 1.44–1.41 (8H, m, H_2-16, H_2-17); ^{13}C NMR (CD_3OD, 100 MHz) δ 169.7 (C-8), 148.0 (C-7), 128.8 (C-2), 128.6 (C-3a/C-7a), 128.4 (C-3a/C-7a), 122.9 (C-5), 114.3 (C-4), 111.4 (C-3), 103.6 (C-6), 55.9 (OMe), 48.9 (C-14), 46.1 (C-12), 36.5 (C-10), 29.9 (C-17), 28.1 (C-11), 27.4 (C-16), 27.3 (C-15); (+)-HRESIMS $[M+H]^+$ *m/z* 605.3810 (calcd for $C_{34}H_{49}N_6O_4$, 605.3810).

N[1],*N*[10]-Bis(3-(7-methoxy-1*H*-indole-3-carboxamido)propyl) decane-1,10-diaminium 2,2,2-trifluoroacetate (**17e**)

Following general procedure A, reaction of 7-methoxy-1*H*-indole-3-carboxylic acid (0.040 g, 0.209 mmol) with EDC·HCl (0.047 g, 0.247 mmol), HOBt (0.032 g, 0.247 mmol), DIPEA (0.10 mL, 0.571 mmol) and di-*tert*-butyl decane-1,10-diylbis((3-aminopropyl)carbamate) **11e** (0.046 g, 0.095 mmol) afforded di-*tert*-butyl decane-1,10-diylbis((3-(7-methoxy-1*H*-indole-3-carboxamido)propyl)carbamate) (0.007, 9%) as a clear colorless oil. Following general procedure B, a subsample of this product (0.005 g, 0.006 mmol) was reacted with TFA in CH_2Cl_2 to afford, after chromatography, the di-TFA salt **17e** (0.002 g, 39%) as a pale yellow oil. R_f (RP-18, 10% *aq* HCl:MeOH 1:3) 0.47; IR (ATR) ν_{max} 3312, 2847, 1671, 1627, 1546, 1501,

1455, 1433, 1312, 1281, 1257, 1200, 1184, 1129, 1068, 1033, 836, 791, 721 cm^{-1}; ^{1}H NMR (CD_3OD, 400 MHz) δ 7.84 (2H, s, H-2), 7.68 (2H, dd, *J* = 8.1, 1.1 Hz, H-4), 7.08 (2H, dd, *J* = 8.1, 7.8 Hz, H-5), 6.72 (2H, d, *J* = 7.8 Hz, H-6), 3.96 (6H, s, OMe), 3.52 (4H, t, *J* = 6.3 Hz, H_2-10), 3.05 (4H, t, *J* = 6.8 Hz, H_2-12), 3.00 (4H, t, *J* = 7.7 Hz, H_2-14), 1.98 (4H, tt, *J* = 6.6, 6.5 Hz, H_2-11), 1.74 (4H, tt, *J* = 7.6, 7.5 Hz, H_2-15), 1.46–1.38 (12H, m, H_2-16, H_2-17, H_2-18); ^{13}C NMR (CD_3OD, 100 MHz) δ 169.8 (C-8), 148.1 (C-7), 128.8 (C-2), 128.6 (C-3a/C-7a), 128.5 (C-3a/C-7a), 122.9 (C-5), 114.3 (C-4), 111.4 (C-3), 103.6 (C-6), 55.9 (OMe), 48.5 (C-14, obscured by solvent), 46.1 (C-12), 36.5 (C-10), 30.4 (C-18), 30.2 (C-17), 28.2 (C-11), 27.6 (C-16), 27.4 (C-15); (+)-HRESIMS [M+H]$^+$ *m/z* 633.4125 (calcd for $C_{36}H_{53}N_6O_4$, 633.4123).

N^1,N^{12}-Bis(3-(7-methoxy-1*H*-indole-3-carboxamido)propyl) dodecane-1,12-diaminium 2,2,2-trifluoroacetate (**17f**)

Following general procedure A, reaction of 7-methoxy-1*H*-indole-3-carboxylic acid (0.040 g, 0.209 mmol) with EDC·HCl (0.047 g, 0.247 mmol), HOBt (0.032 g, 0.247 mmol), DIPEA (0.10 mL, 0.571 mmol) and di-*tert*-butyl dodecane-1,12-diylbis((3-aminopropyl)carbamate) **11f** (0.049 g, 0.095 mmol) afforded di-*tert*-butyl dodecane-1,12-diylbis((3-(7-methoxy-1*H*-indole-3-carboxamido)propyl)carbamate) (0.024 g, 29%) as a clear colorless oil. Following general procedure B, a subsample of this product (0.012 g, 0.014 mmol) was reacted with TFA in CH_2Cl_2 to afford, after chromatography, the di-TFA salt **17f** (0.011 g, 89%) as a yellow oil. R_f (RP-18, 10% *aq* HCl:MeOH 1:3) 0.47; IR (ATR) ν_{max} 3318, 2845, 1672, 1627, 1546, 1501, 1455, 1433, 1313, 1280, 1257, 1200, 1184, 1130, 1068, 1034, 836, 791, 721 cm^{-1}; ^{1}H NMR (CD_3OD, 400 MHz) δ 7.85 (2H, s, H-2), 7.68 (2H, d, *J* = 7.8 Hz, H-4), 7.08 (2H, dd, *J* = 7.8, 7.6 Hz, H-5), 6.72 (2H, d, *J* = 7.6 Hz, H-6), 3.96 (6H, s, OMe), 3.52 (4H, t, *J* = 6.3 Hz, H_2-10), 3.05 (4H, t, *J* = 6.8 Hz, H_2-12), 3.00 (4H, t, *J* = 7.7 Hz, H_2-14), 1.98 (4H, tt, *J* = 6.6, 6.5 Hz, H_2-11), 1.73 (4H, tt, *J* = 7.5, 7.5 Hz, H_2-15), 1.46–1.33 (16H, m, H_2-16, H_2-17, H_2-18, H_2-19); ^{13}C NMR (CD_3OD, 100 MHz) δ 169.7 (C-8), 148.0 (C-7), 128.8 (C-2), 128.6 (C-3a/C-7a), 128.4 (C-3a/C-7a), 122.9 (C-5), 114.3 (C-4), 111.5 (C-3), 103.6 (C-6), 55.9 (OMe), 48.7 (C-14, obscured by solvent), 46.2 (C-12), 36.5 (C-10), 30.6 (C-19), 30.5 (C-18), 30.2 (C-17), 28.2 (C-11), 27.5 (C-16), 27.4 (C-15); (+)-HRESIMS [M+H]$^+$ *m/z* 661.4439 (calcd for $C_{38}H_{57}N_6O_4$, 661.4436).

N^1,N^4-Bis(3-(7-methyl-1*H*-indole-3-carboxamido)propyl)butane-1,4-diaminium 2,2,2-trifluoroacetate (**18a)**

Following general procedure A, reaction of 7-methyl-1*H*-indole-3-carboxylic acid (0.050 g, 0.285 mmol) with EDC·HCl (0.065 g, 0.337 mmol), HOBt (0.044 g, 0.337 mmol), DIPEA (0.14 mL, 0.778 mmol) and di-*tert*-butyl butane-1,4-diylbis((3-aminopropyl)carbamate) **11a** (0.052 g, 0.130 mmol) afforded di-*tert*-butyl butane-1,4-diylbis((3-(7-methyl-1*H*-indole-3-carboxamido)propyl)carbamate) (0.008 g, 9%) as a clear colorless oil. Following general procedure B, a subsample of this product (0.004 g, 0.006 mmol) was reacted with TFA in CH_2Cl_2 to afford, after chromatography, the di-TFA salt **18a** (0.004 g, 96%) as a yellow oil. R_f (RP-18, 10% *aq* HCl:MeOH 1:3) 0.58; IR (ATR) ν_{max} 3272, 2940, 2858, 1672, 1616, 1544, 1499, 1441, 1383, 1312, 1262, 1199, 1180, 1131, 835, 791, 746, 721 cm^{-1}; ^{1}H NMR (CD_3OD, 400 MHz) δ 7.92 (2H, d, *J* = 8.0 Hz, H-4), 7.90 (2H, d, *J* = 1.4 Hz, H-2), 7.06 (2H, dd, *J* = 8.0, 7.0 Hz, H-5), 6.98 (2H, d, *J* = 7.0 Hz, H-6), 3.52 (4H, t, *J* = 6.2 Hz, H_2-10), 3.09–3.06 (8H, m, H_2-12, H_2-14), 2.50 (6H, s, Me), 1.99 (4H, tt, *J* = 6.6, 6.5 Hz, H_2-11), 1.91 (4H, tt, *J* = 3.5, 3.5 Hz, H_2-15); ^{13}C NMR (CD_3OD, 100 MHz) δ 169.9 (C-8), 137.6 (C-7a), 129.3 (C-2), 126.8 (C-3a), 124.2 (C-6), 122.7 (C-7), 122.5 (C-5), 119.3 (C-4), 111.3 (C-3), 48.1 (C-14), 46.2 (C-12), 36.5 (C-10), 28.2 (C-11), 24.5 (C-15), 16.8 (Me); (+)-HRESIMS [M+H]$^+$ *m/z* 517.3282 (calcd for, $C_{30}H_{41}N_6O_2$, 517.3286).

N^1,N^6-Bis(3-(7-methyl-1*H*-indole-3-carboxamido)propyl) hexane-1,6-diaminium 2,2,2-trifluoroacetate (**18b**)

Following general procedure A, reaction of 7-methyl-1*H*-indole-3-carboxylic acid (0.050 g, 0.285 mmol) with EDC·HCl (0.065 g, 0.337 mmol), HOBt (0.044 g, 0.337 mmol), DIPEA (0.14 mL, 0.778 mmol) and di-*tert*-butyl hexane-1,6-diylbis((3-aminopropyl)carbamate) **11b** (0.056 g, 0.130 mmol) afforded di-*tert*-butyl hexane-1,6-diylbis((3-(7-methyl-1*H*-indole-3-carboxamido)propyl)carbamate) (0.024 g, 25%) as a clear colorless oil. Following general procedure B, a subsample of this product (0.012 g, 0.016 mmol) was reacted with TFA in CH_2Cl_2 to afford, after chromatography, the di-TFA salt **18b** (0.002 g, 16%) as

a yellow oil. R_f (RP-18, 10% *aq* HCl:MeOH 1:3) 0.57; IR (ATR) ν_{max} 3261, 2939, 2854, 1671, 1617, 1543, 1499, 1436, 1383, 1312, 1262, 1199, 1180, 1131, 835, 790, 746, 721 cm^{-1}; ^1H NMR (CD_3OD, 400 MHz) δ 7.93 (2H, d, *J* = 8.1 Hz, H-4), 7.91 (2H, d, *J* = 1.8 Hz, H-2), 7.06 (2H, dd, *J* = 8.1, 7.2 Hz, H-5), 6.98 (2H, d, *J* = 7.2 Hz, H-6), 3.52 (4H, t, *J* = 6.3 Hz, H_2-10), 3.04 (4H, t, *J* = 6.8 Hz, H_2-12), 3.01 (4H, t, *J* = 7.5 Hz, H_2-14), 2.50 (6H, s, Me), 1.98 (4H, tt, *J* = 6.6, 6.5 Hz, H_2-11), 1.77 (4H, tt, *J* = 7.2, 7.1 Hz, H_2-15), 1.52 (4H, tt, *J* = 3.8, 3.7 Hz, H_2-16); ^{13}C NMR (CD_3OD, 100 MHz) δ 169.8 (C-8), 137.5 (C-7a), 129.2 (C-2), 126.8 (C-3a), 124.1 (C-6), 122.6 (C-7), 122.4 (C-5), 119.3 (C-4), 111.3 (C-3), 48.7 (C-14, obscured by solvent), 46.1 (C-12), 36.5 (C-10), 28.2 (C-11), 27.12 (C-16), 27.06 (C-15), 16.8 (Me); (+)-HRESIMS $[M+H]^+$ *m/z* 545.3597 (calcd for $C_{32}H_{45}N_6O_2$, 545.3599).

N^1,N^7-Bis(3-(7-methyl-1*H*-indole-3-carboxamido)propyl) heptane-1,7-diaminium 2,2,2-trifluoroacetate (**18c**)

Following general procedure A, reaction of 7-methyl-1*H*-indole-3-carboxylic acid (0.050 g, 0.285 mmol) with EDC·HCl (0.065 g, 0.337 mmol), HOBt (0.044 g, 0.337 mmol), DIPEA (0.14 mL, 0.778 mmol) and di-*tert*-butyl heptane-1,7-diylbis((3-aminopropyl)carbamate) **11c** (0.058 g, 0.130 mmol) afforded di-*tert*-butyl heptane-1,7-diylbis((3-(7-methyl-1*H*-indole-3-carboxamido)propyl)carbamate) (0.064 g, 65%) as a clear colorless oil. Following general procedure B, a subsample of this product (0.032 g, 0.042 mmol) was reacted with TFA in CH_2Cl_2 to afford, after chromatography, the di-TFA salt **18c** (0.030 g, 90%) as a yellow oil. R_f (RP-18, 10% *aq* HCl:MeOH 1:3) 0.55; IR (ATR) ν_{max} 3265, 2940, 2856, 1672, 1616, 1543, 1499, 1441, 1383, 1312, 1262, 1199, 1180, 1131, 835, 791, 746, 721 cm^{-1}; ^1H NMR (CD_3OD, 400 MHz) δ 7.93 (2H, obscured by H-2, H-4), 7.92 (2H, br s, H-2), 7.05 (2H, dd, *J* = 7.6, 7.1 Hz, H-5), 6.98 (2H, d, *J* = 7.1 Hz, H-6), 3.51 (4H, t, *J* = 6.2 Hz, H_2-10), 3.03 (4H, t, *J* = 6.9 Hz, H_2-12), 2.97 (4H, t, *J* = 7.6 Hz, H_2-14), 2.49 (6H, s, Me), 1.98 (4H, tt, *J* = 6.5, 6.4 Hz, H_2-11), 1.72 (4H, tt, *J* = 6.6, 6.5 Hz, H_2-15), 1.47–1.39 (6H, m, H_2-16, H_2-17); ^{13}C NMR (CD_3OD, 100 MHz) δ 169.7 (C-8), 137.5 (C-7a), 129.2 (C-2), 126.8 (C-3a), 124.1 (C-6), 122.6 (C-7), 122.4 (C-5), 119.3 (C-4), 111.3 (C-3), 49.8 (C-14), 46.1 (C-12), 36.5 (C-10), 29.6 (C-17), 28.1 (C-11), 27.2 (C-16), 27.1 (C-15), 16.8 (Me); (+)-HRESIMS $[M+H]^+$ *m/z* 559.3762 (calcd for $C_{33}H_{47}N_6O_2$, 559.3755).

N^1,N^8-Bis(3-(7-methyl-1*H*-indole-3-carboxamido)propyl) octane-1,8-diaminium 2,2,2-trifluoroacetate (**18d**)

Following general procedure A, reaction of 7-methyl-1*H*-indole-3-carboxylic acid (0.050 g, 0.285 mmol) with EDC·HCl (0.065 g, 0.337 mmol), HOBt (0.044 g, 0.337 mmol), DIPEA (0.14 mL, 0.778 mmol) and di-*tert*-butyl octane-1,8-diylbis((3-aminopropyl)carbamate) **11d** (0.060 g, 0.130 mmol) afforded di-*tert*-butyl octane-1,8-diylbis((3-(7-methyl-1*H*-indole-3-carboxamido)propyl)carbamate) (0.024 g, 24%) as a clear colorless oil. Following general procedure B, a subsample of this product (0.012 g, 0.016 mmol) was reacted with TFA in CH_2Cl_2 to afford, after chromatography, the di-TFA salt **18d** (0.012 g, 97%) as a yellow oil. R_f (RP-18, 10% *aq* HCl:MeOH 1:3) 0.53; IR (ATR) ν_{max} 3261, 2940, 2860, 1671, 1616, 1542, 1499, 1441, 1383, 1312, 1262, 1199, 1180, 1131, 835, 791, 746, 721 cm^{-1}; ^1H NMR (CD_3OD, 400 MHz) δ 7.93 (2H, d, *J* = 8.1 Hz, H-4), 7.92 (2H, d, *J* = 1.7 Hz, H-2), 7.06 (2H, dd, *J* = 8.1, 7.2 Hz, H-5), 6.98 (2H, d, *J* = 7.2 Hz, H-6), 3.51 (4H, t, *J* = 6.2 Hz, H_2-10), 3.04 (4H, t, *J* = 6.9 Hz, H_2-12), 2.98 (4H, t, *J* = 7.7 Hz, H_2-14), 2.50 (6H, s, Me), 1.98 (4H, tt, *J* = 6.6, 6.6 Hz, H_2-11), 1.73 (4H, tt, *J* = 7.4, 7.4 Hz, H_2-15), 1.48–1.43 (4H, m, H_2-16), 1.43–1.38 (4H, m, H_2-17); ^{13}C NMR (CD_3OD, 100 MHz) δ 169.8 (C-8), 137.6 (C-7a), 129.2 (C-2), 126.9 (C-3a), 124.2 (C-6), 122.6 (C-7), 122.4 (C-5), 119.4 (C-4), 111.4 (C-3), 49.0 (C-14), 46.2 (C-12), 36.6 (C-10), 29.9 (C-17), 28.2 (C-11), 27.4 (C-16), 27.3 (C-15), 16.8 (Me); (+)-HRESIMS $[M+H]^+$ *m/z* 573.3911 (calcd for $C_{34}H_{49}N_6O_2$, 573.3912).

N^1,N^{10}-Bis(3-(7-methyl-1*H*-indole-3-carboxamido)propyl) decane-1,10-diaminium 2,2,2-trifluoroacetate (**18e**)

Following general procedure A, reaction of 7-methyl-1*H*-indole-3-carboxylic acid (0.050 g, 0.285 mmol) with EDC·HCl (0.065 g, 0.337 mmol), HOBt (0.044 g, 0.337 mmol), DIPEA (0.14 mL, 0.778 mmol) and di-*tert*-butyl decane-1,10-diylbis((3-aminopropyl)carbamate) **11e** (0.063 g, 0.130 mmol) afforded di-*tert*-butyl decane-1,10-diylbis((3-(7-methyl-1*H*-indole-3-carboxamido)propyl)carbamate) (0.044 g, 42%) as a

clear colorless oil. Following general procedure B, a subsample of this product (0.022 g, 0.027 mmol) was reacted with TFA in CH_2Cl_2 to afford, after chromatography, the di-TFA salt **18e** (0.012 g, 53%) as a yellow oil. R_f (RP-18, 10% *aq* HCl:MeOH 1:3) 0.50; IR (ATR) ν_{max} 3266, 2941, 2858, 1672, 1616, 1543, 1499, 1441, 1383, 1312, 1262, 1199, 1180, 1131, 835, 791, 746, 721 cm^{-1}; ^1H NMR (CD_3OD, 400 MHz) δ 7.93 (2H, d, *J* = 8.0 Hz, H-4), 7.92 (2H, s, H-2), 7.05 (2H, dd, *J* = 8.0, 7.0 Hz, H-5), 6.98 (2H, d, *J* = 7.0 Hz, H-6), 3.51 (4H, t, *J* = 6.3 Hz, H_2-10), 3.04 (4H, t, *J* = 6.9 Hz, H_2-12), 2.97 (4H, t, *J* = 7.7 Hz, H_2-14), 2.50 (6H, s, Me), 1.98 (4H, tt, *J* = 6.6, 6.5 Hz, H_2-11), 1.71 (4H, tt, *J* = 7.5, 7.5 Hz, H_2-15), 1.42–1.34 (12H, m, H_2-16, H_2-17, H_2-18); ^{13}C NMR (CD_3OD, 100 MHz) δ 169.7 (C-8), 137.5 (C-7a), 129.2 (C-2), 126.8 (C-3a), 124.1 (C-6), 122.6 (C-7), 122.4 (C-5), 119.4 (C-4), 111.3 (C-3), 49.8 (C-14), 46.2 (C-12), 36.6 (C-10), 30.3 (C-18), 30.1 (C-17), 28.1 (C-11), 27.5 (C-16), 27.4 (C-15), 16.8 (Me); (+)-HRESIMS $[M+H]^+$ *m/z* 601.4226 (calcd for $C_{36}H_{53}N_6O_2$, 601.4225).

N^1,N^{12}-Bis(3-(7-methyl-1*H*-indole-3-carboxamido)propyl) dodecane-1,12-diaminium 2,2,2-trifluoroacetate (**18f**)

Following general procedure A, reaction of 7-methyl-1*H*-indole-3-carboxylic acid (0.050 g, 0.285 mmol) with EDC·HCl (0.065 g, 0.337 mmol), HOBt (0.044 g, 0.337 mmol), DIPEA (0.14 mL, 0.778 mmol) and di-*tert*-butyl dodecane-1,12-diylbis((3-aminopropyl)carbamate) **11f** (0.067 g, 0.130 mmol) afforded di-*tert*-butyl dodecane-1,12-diylbis((3-(7-methyl-1*H*-indole-3-carboxamido)propyl)carbamate) (0.056 g, 52%) as a clear colorless oil. Following general procedure B, a subsample of this product (0.028 g, 0.034 mmol) was reacted with TFA in CH_2Cl_2 to afford, after chromatography, the di-TFA salt **18f** (0.007 g, 24%) as a yellow oil. R_f (RP-18, 10% *aq* HCl:MeOH 1:3) 0.48; IR (ATR) ν_{max} 3259, 2941, 2860, 1671, 1617, 1543, 1499, 1436, 1383, 1312, 1262, 1199, 1180, 1131, 836, 791, 746, 721 cm^{-1}; ^1H NMR (CD_3OD, 400 MHz) δ 7.94 (2H, d, *J* = 8.0 Hz, H-4), 7.92 (2H, d, *J* = 1.5 Hz, H-2), 7.06 (2H, dd, *J* = 8.0, 7.6 Hz, H-5), 6.99 (2H, d, *J* = 7.6 Hz, H-6), 3.52 (4H, t, *J* = 6.3 Hz, H_2-10), 3.05 (4H, t, *J* = 6.9 Hz, H_2-12), 2.98 (4H, t, *J* = 7.6 Hz, H_2-14), 2.50 (6H, s, Me), 1.98 (4H, tt, *J* = 6.6, 6.5 Hz, H_2-11), 1.72 (4H, tt, *J* = 7.5, 7.5 Hz, H_2-15), 1.43–1.31 (16H, m, H_2-16, H_2-17, H_2-18); ^{13}C NMR (CD_3OD, 100 MHz) δ 169.7 (C-8), 137.5 (C-7a), 129.2 (C-2), 126.8 (C-3a), 124.1 (C-6), 122.6 (C-7), 122.4 (C-5), 119.4 (C-4), 111.3 (C-3), 49.8 (C-14), 46.2 (C-12), 36.5 (C-10), 30.6 (C-19), 30.4 (C-18), 30.2 (C-17), 28.1 (C-11), 27.5 (C-16), 27.4 (C-15), 16.8 (Me); (+)-HRESIMS $[M+H]^+$ *m/z* 629.4536 (calcd for $C_{38}H_{57}N_6O_2$, 629.4538).
