# Supplementary material for: Antimicrobial Indole-3-Carboxamido-Polyamine Conjugates Target Bacterial Membranes and Are Antibiotic Potentiators
Source: Biomolecules. 2024 Feb 22;14(3):261. doi: 10.3390/biom14030261 (PMC10967822; doi:10.3390/biom14030261)
Supplement: Supplementary file 1 [file biomolecules-14-00261-s001.zip › supplemetary-Figures.pdf]

Supplementary File

Indole-3-carboxamido-Polyamine Conjugates Target Bacterial Membranes and are Antimicrobial and Antibiotic Potentiators

Kenneth Sue [1], Melissa M. Cadelis [1,2], Florent Rouvier [3], Marie-Lise Bourguet-Kondracki [4], Jean Michel Brunel [3] and Brent R. Copp [1,*]

[1] School of Chemical Sciences, The University of Auckland, Private Bag 92019, Auckland 1142, New Zealand
[2] School of Medical Sciences, The University of Auckland, Private Bag 92019, Auckland 1142, New Zealand
[3] Membranes et Cibles Thérapeutiques, INSERM, Aix-Marseille Universite, 27 bd Jean Moulin, 13385 Marseille, France
[4] Laboratoire Molécules de Communication et Adaptation des Micro-organismes, UMR 7245 CNRS, Muséum National d'Histoire Naturelle, 57 rue Cuvier (C.P. 54), 75005 Paris, France
* Correspondence: b.copp@auckland.ac.nz

Contents

Figure S1 ^{1}H (CD_3OD, 400 MHz) and ^{13}C (CD_3OD, 100 MHz) NMR spectra for **12a** **S3**
Figure S2 ^{1}H (CD_3OD, 400 MHz) and ^{13}C (CD_3OD, 100 MHz) NMR spectra for **12b** **S4**
Figure S3 ^{1}H (CD_3OD, 400 MHz) and ^{13}C (CD_3OD, 100 MHz) NMR spectra for **12c** **S5**
Figure S4 ^{1}H (CD_3OD, 400 MHz) and ^{13}C (CD_3OD, 100 MHz) NMR spectra for **12d** **S6**
Figure S5 ^{1}H (CD_3OD, 400 MHz) and ^{13}C (CD_3OD, 100 MHz) NMR spectra for **12e** **S7**
Figure S6 ^{1}H (CD_3OD, 400 MHz) and ^{13}C (CD_3OD, 100 MHz) NMR spectra for **12f** **S8**
Figure S7 ^{1}H (CD_3OD, 400 MHz) and ^{13}C (CD_3OD, 100 MHz) NMR spectra for **13b** **S9**
Figure S8 ^{1}H (CD_3OD, 400 MHz) and ^{13}C (CD_3OD, 100 MHz) NMR spectra for **13c** **S10**
Figure S9 ^{1}H (CD_3OD, 400 MHz) and ^{13}C (CD_3OD, 100 MHz) NMR spectra for **13d** **S11**
Figure S10 ^{1}H (CD_3OD, 400 MHz) and ^{13}C (CD_3OD, 100 MHz) NMR spectra for **13e** **S12**
Figure S11 ^{1}H (CD_3OD, 400 MHz) and ^{13}C (CD_3OD, 100 MHz) NMR spectra for **13f** **S13**
Figure S12 ^{1}H (CD_3OD, 400 MHz) and ^{13}C (CD_3OD, 100 MHz) NMR spectra for **14a** **S14**
Figure S13 ^{1}H (CD_3OD, 400 MHz) and ^{13}C (CD_3OD, 100 MHz) NMR spectra for **14b** **S15**
Figure S14 ^{1}H (CD_3OD, 400 MHz) and ^{13}C (CD_3OD, 100 MHz) NMR spectra for **14c** **S16**
Figure S15 ^{1}H (CD_3OD, 400 MHz) and ^{13}C (CD_3OD, 100 MHz) NMR spectra for **14d** **S17**
Figure S16 ^{1}H (CD_3OD, 400 MHz) and ^{13}C (CD_3OD, 100 MHz) NMR spectra for **14e** **S18**
Figure S17 ^{1}H (CD_3OD, 400 MHz) and ^{13}C (CD_3OD, 100 MHz) NMR spectra for **14f** **S19**
Figure S18 ^{1}H (CD_3OD, 400 MHz) and ^{13}C (CD_3OD, 100 MHz) NMR spectra for **15a** **S20**

Figure S19 ^{1}H (CD_3OD, 400 MHz) and ^{13}C (CD_3OD, 100 MHz) NMR spectra for **15b** **S21**

Figure S20 ^{1}H (CD_3OD, 400 MHz) and ^{13}C (CD_3OD, 100 MHz) NMR spectra for **15c** **S22**

Figure S21 ^{1}H (CD_3OD, 400 MHz) and ^{13}C (CD_3OD, 100 MHz) NMR spectra for **15d** **S23**

Figure S22 ^{1}H (CD_3OD, 400 MHz) and ^{13}C (CD_3OD, 100 MHz) NMR spectra for **15e** **S24**

Figure S23 ^{1}H (CD_3OD, 400 MHz) and ^{13}C (CD_3OD, 100 MHz) NMR spectra for **15f** **S25**

Figure S24 ^{1}H (CD_3OD, 400 MHz) and ^{13}C (CD_3OD, 100 MHz) NMR spectra for **16a** **S26**

Figure S25 ^{1}H (CD_3OD, 400 MHz) and ^{13}C (CD_3OD, 100 MHz) NMR spectra for **16b** **S27**

Figure S26 ^{1}H (CD_3OD, 400 MHz) and ^{13}C (CD_3OD, 100 MHz) NMR spectra for **16c** **S28**

Figure S27 ^{1}H (CD_3OD, 400 MHz) and ^{13}C (CD_3OD, 100 MHz) NMR spectra for **16d** **S29**

Figure S28 ^{1}H (CD_3OD, 400 MHz) and ^{13}C (CD_3OD, 100 MHz) NMR spectra for **16e** **S30**

Figure S29 ^{1}H (CD_3OD, 400 MHz) and ^{13}C (CD_3OD, 100 MHz) NMR spectra for **16f** **S31**

Figure S30 ^{1}H (CD_3OD, 400 MHz) and ^{13}C (CD_3OD, 100 MHz) NMR spectra for **17a** **S32**

Figure S31 ^{1}H (CD_3OD, 400 MHz) and ^{13}C (CD_3OD, 100 MHz) NMR spectra for **17b** **S33**

Figure S32 ^{1}H (CD_3OD, 400 MHz) and ^{13}C (CD_3OD, 100 MHz) NMR spectra for **17c** **S34**

Figure S33 ^{1}H (CD_3OD, 400 MHz) and ^{13}C (CD_3OD, 100 MHz) NMR spectra for **17d** **S35**

Figure S34 ^{1}H (CD_3OD, 400 MHz) and ^{13}C (CD_3OD, 100 MHz) NMR spectra for **17e** **S36**

Figure S35 ^{1}H (CD_3OD, 400 MHz) and ^{13}C (CD_3OD, 100 MHz) NMR spectra for **17f** **S37**

Figure S36 ^{1}H (CD_3OD, 400 MHz) and ^{13}C (CD_3OD, 100 MHz) NMR spectra for **18a** **S38**

Figure S37 ^{1}H (CD_3OD, 400 MHz) and ^{13}C (CD_3OD, 100 MHz) NMR spectra for **18b** **S39**

Figure S38 ^{1}H (CD_3OD, 400 MHz) and ^{13}C (CD_3OD, 100 MHz) NMR spectra for **18c** **S40**

Figure S39 ^{1}H (CD_3OD, 400 MHz) and ^{13}C (CD_3OD, 100 MHz) NMR spectra for **18d** **S41**

Figure S40 ^{1}H (CD_3OD, 400 MHz) and ^{13}C (CD_3OD, 100 MHz) NMR spectra for **18e** **S42**

Figure S41 ^{1}H (CD_3OD, 400 MHz) and ^{13}C (CD_3OD, 100 MHz) NMR spectra for **18f** **S43**

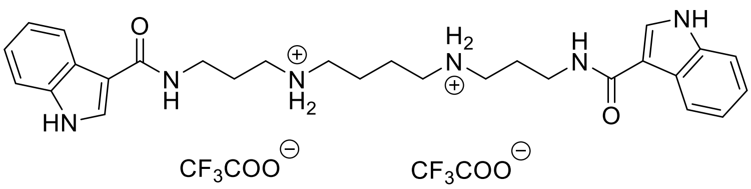

^{1}H NMR (CD_3OD, 400 MHz):

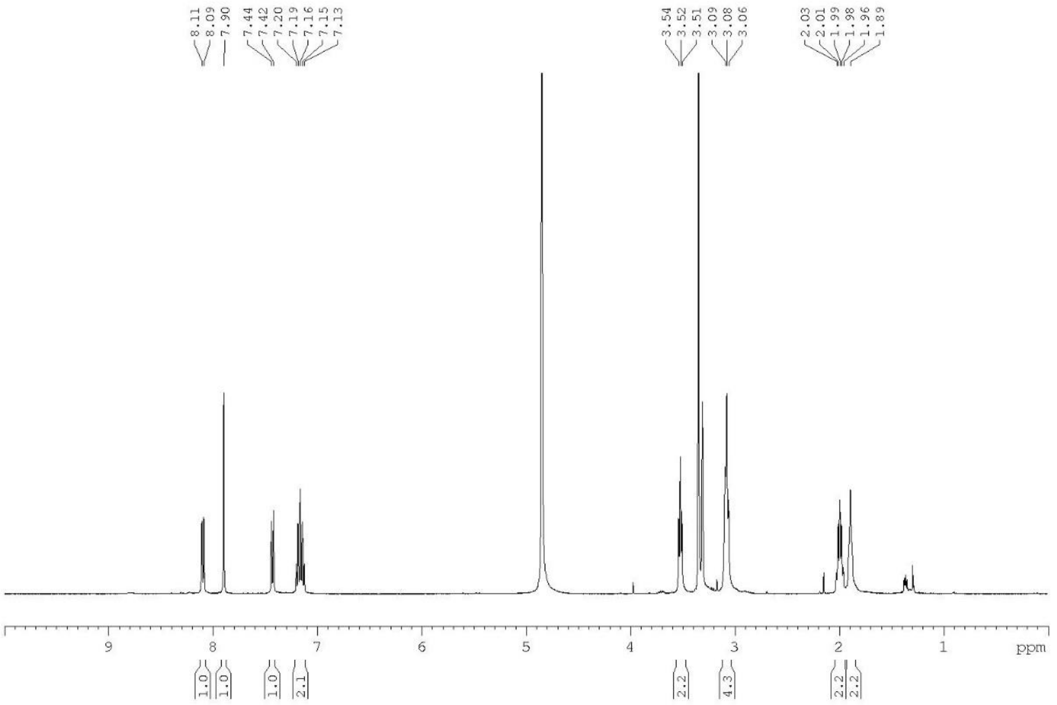

^{13}C NMR (CD_3OD, 100 MHz):

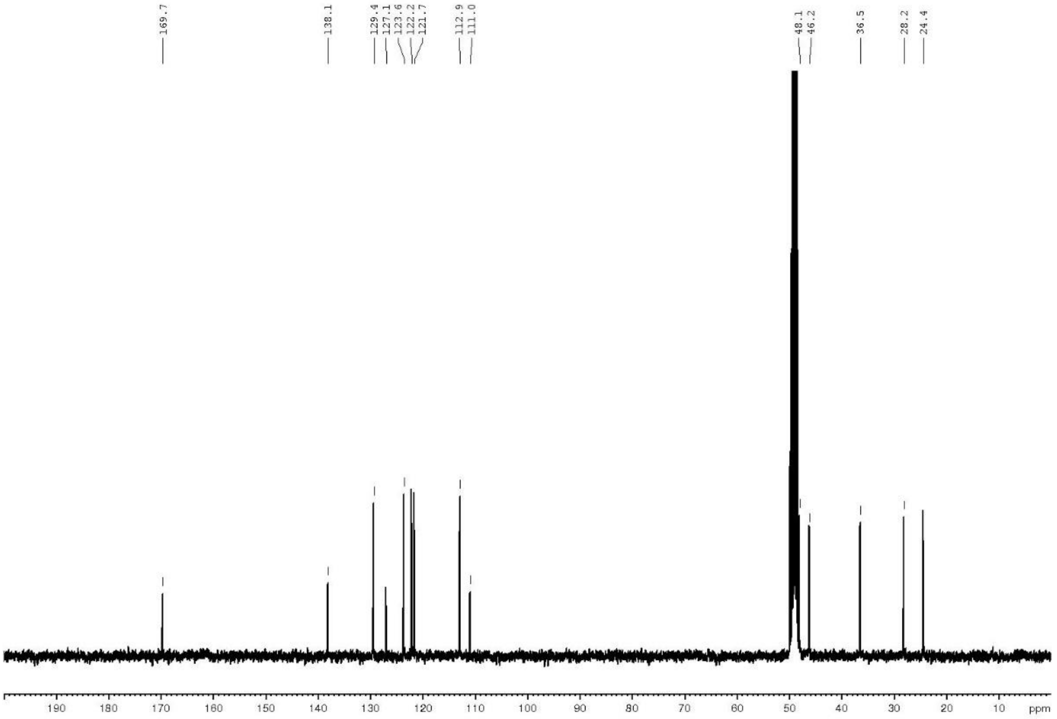

Figure S1 ^{1}H (CD_3OD, 400 MHz) and ^{13}C (CD_3OD, 100 MHz) NMR spectra for **12a**

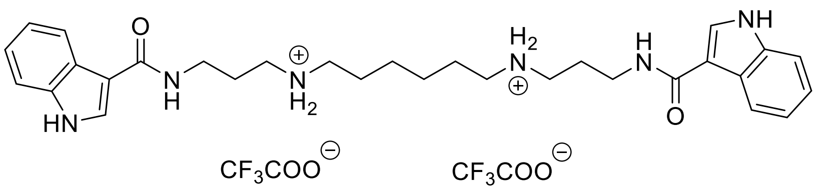

^{1}H NMR (CD_3OD, 400 MHz):

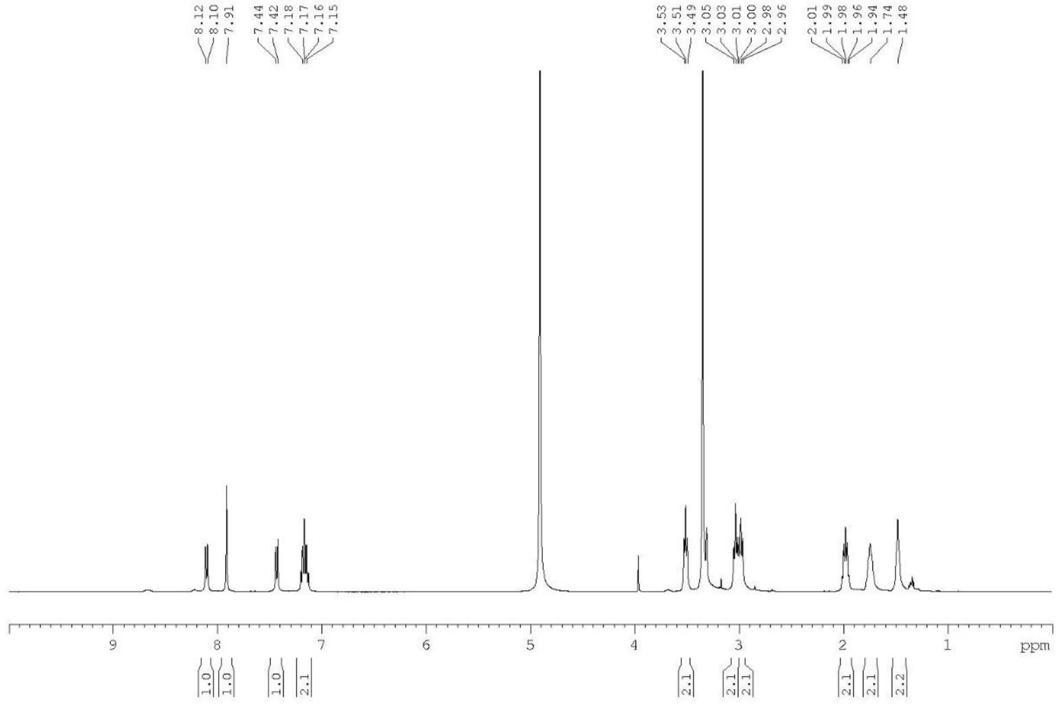

^{13}C NMR (CD_3OD, 100 MHz):

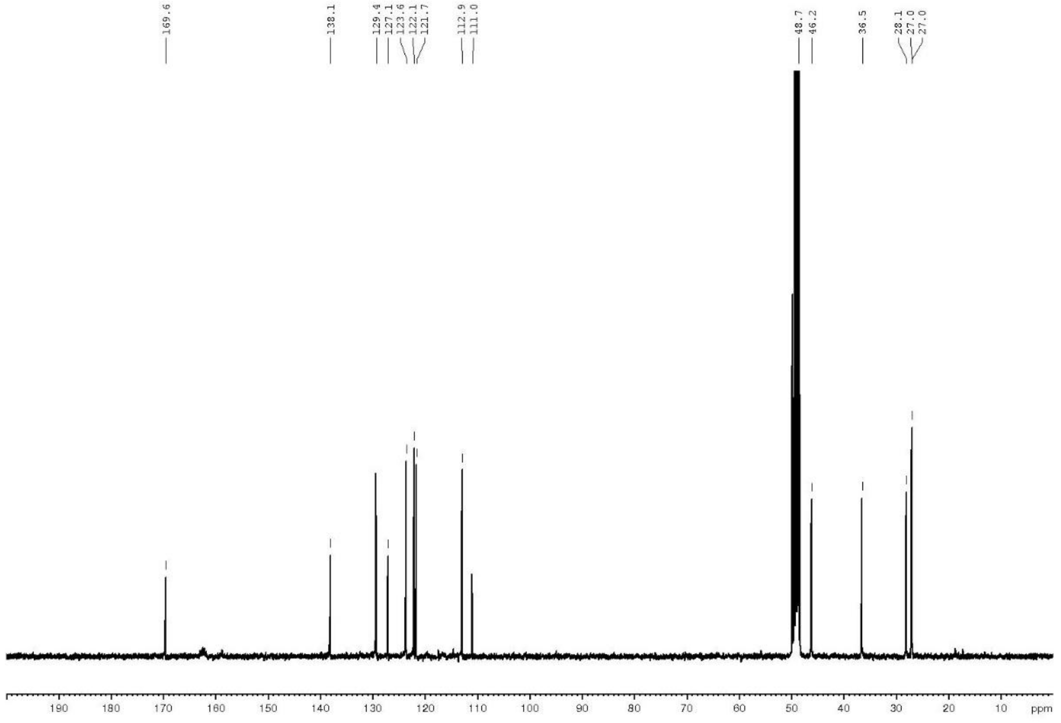

Figure S2 ^{1}H (CD_3OD, 400 MHz) and ^{13}C (CD_3OD, 100 MHz) NMR spectra for **12b**

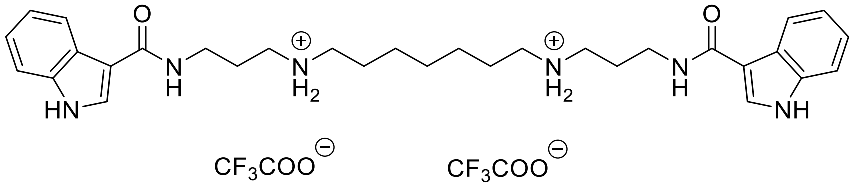

^{1}H NMR (CD_3OD, 400 MHz):

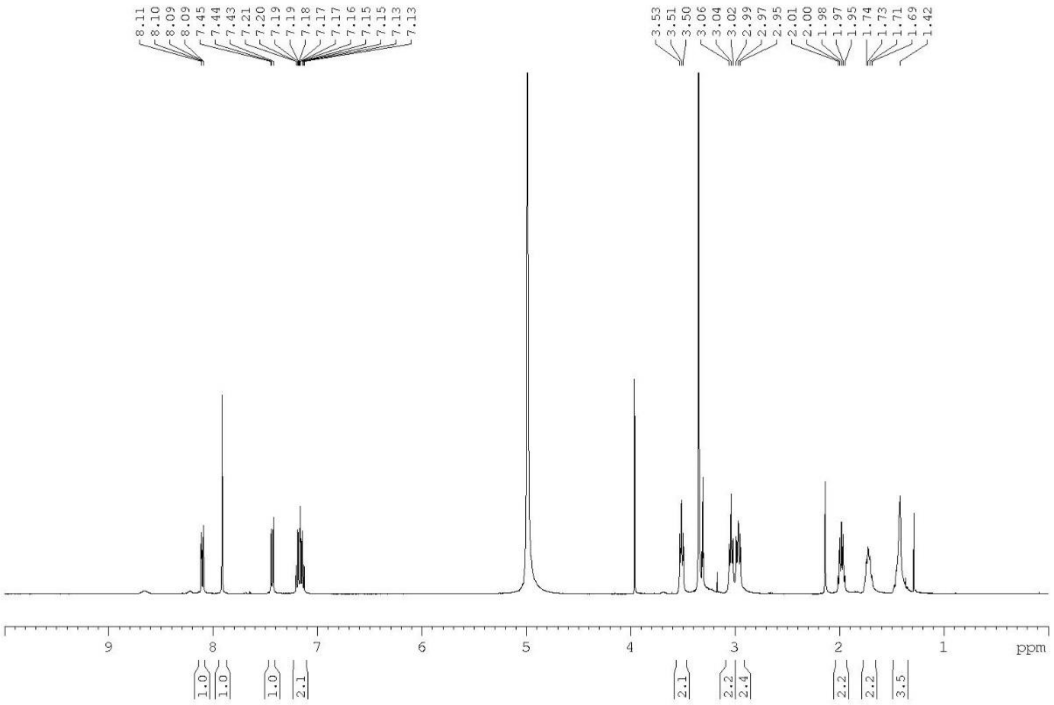

^{13}C NMR (CD_3OD, 100 MHz):

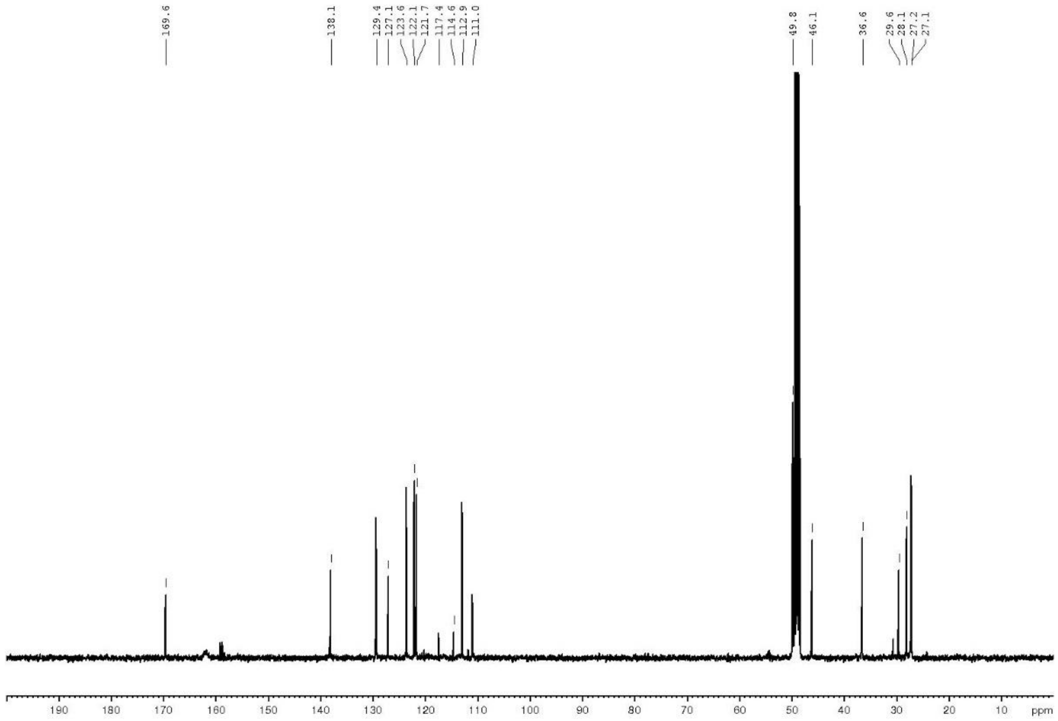

Figure S3 ^{1}H (CD_3OD, 400 MHz) and ^{13}C (CD_3OD, 100 MHz) NMR spectra for **12c**

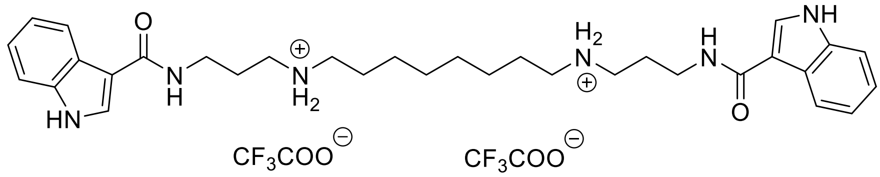

^{1}H NMR (CD$_3$OD, 400 MHz):

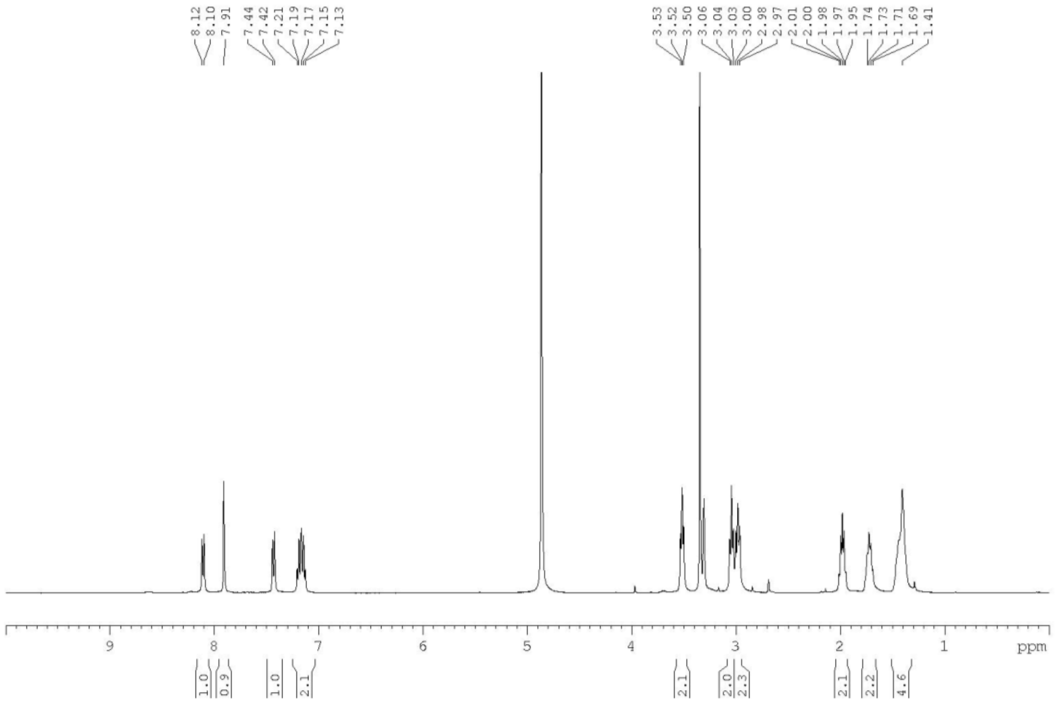

^{13}C NMR (CD$_3$OD, 100 MHz):

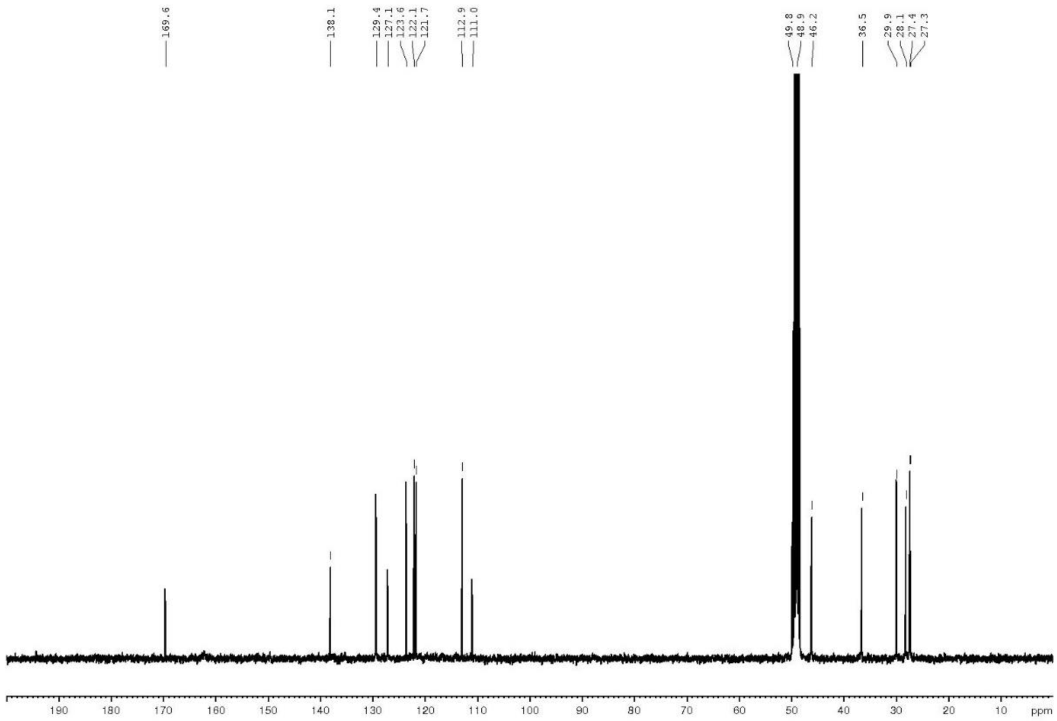

Figure S4 ^{1}H (CD$_3$OD, 400 MHz) and ^{13}C (CD$_3$OD, 100 MHz) NMR spectra for **12d**

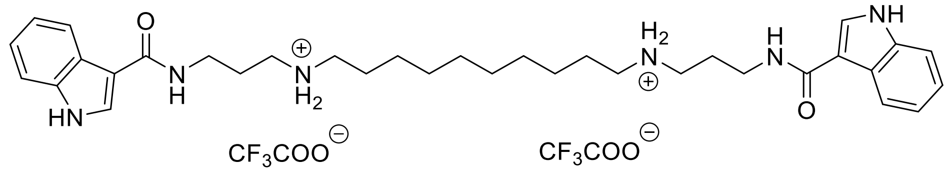

^{1}H NMR (CD_3OD, 400 MHz):

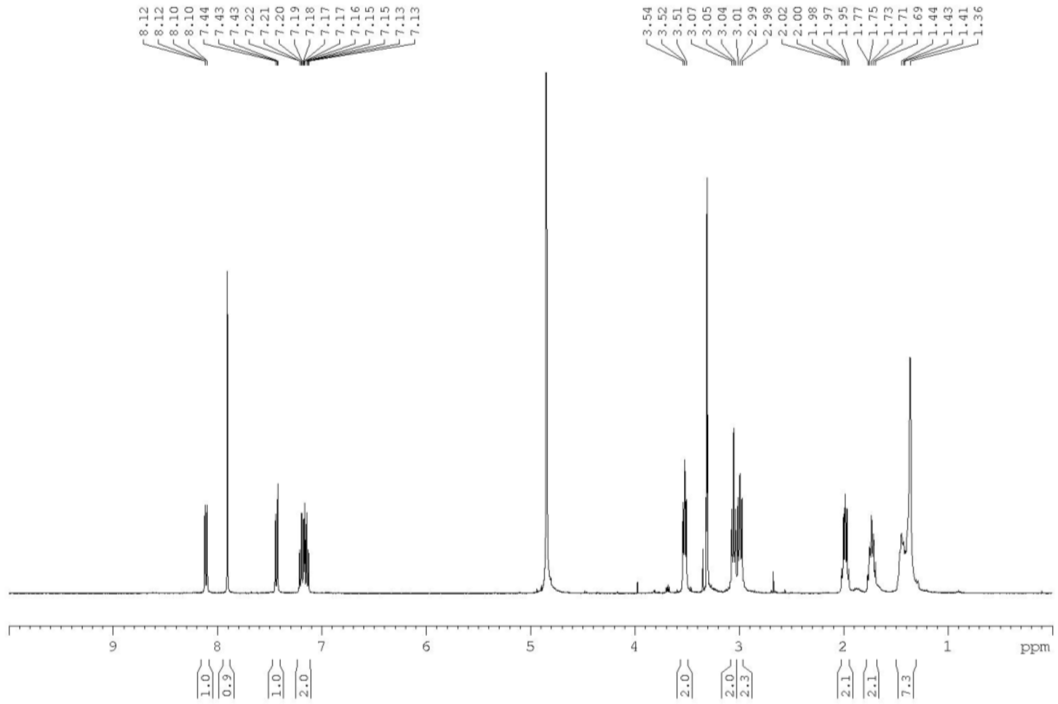

^{13}C NMR (CD_3OD, 100 MHz):

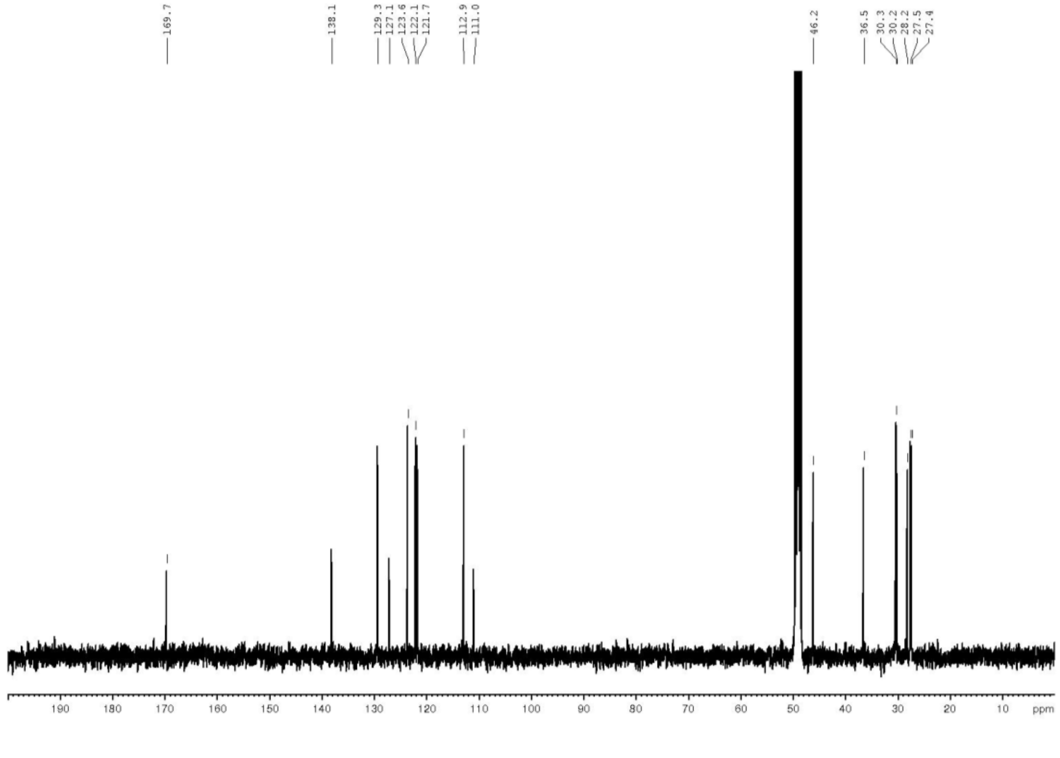

Figure S5 ^{1}H (CD_3OD, 400 MHz) and ^{13}C (CD_3OD, 100 MHz) NMR spectra for **12e**

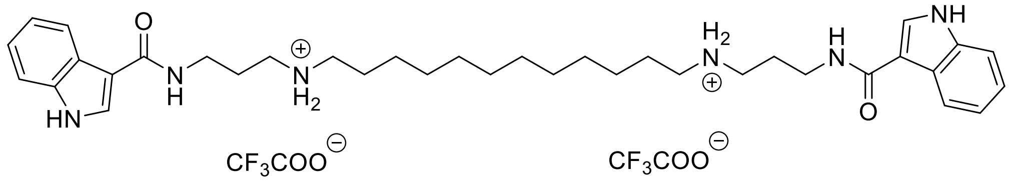

^{1}H NMR (CD$_3$OD, 400 MHz):

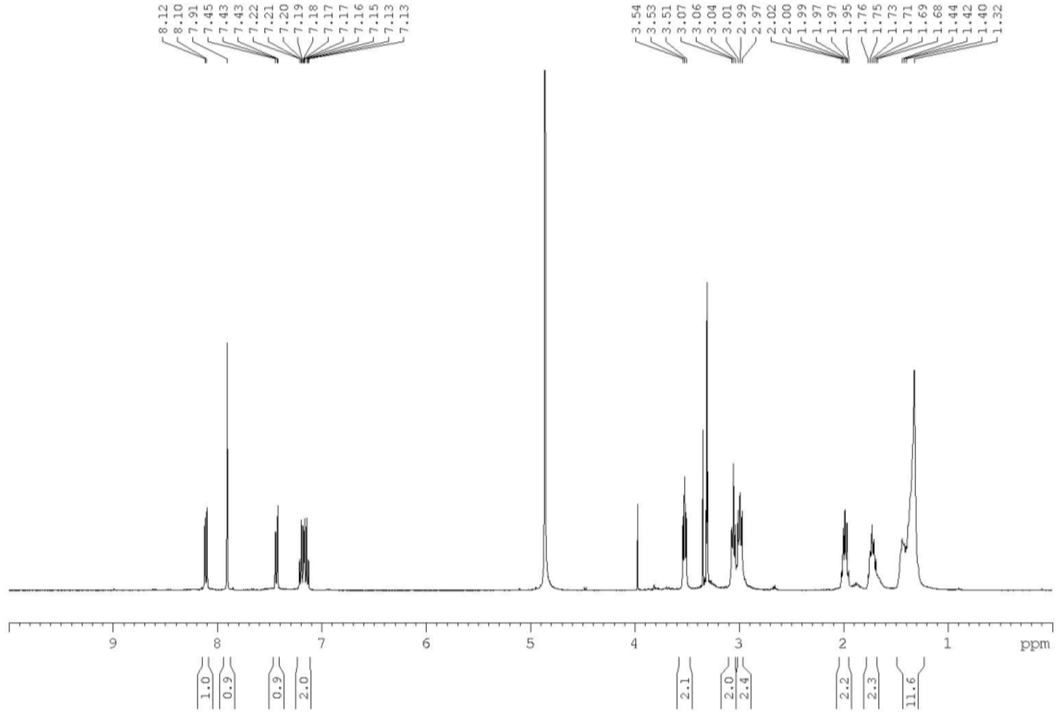

^{13}C NMR (CD$_3$OD, 100 MHz):

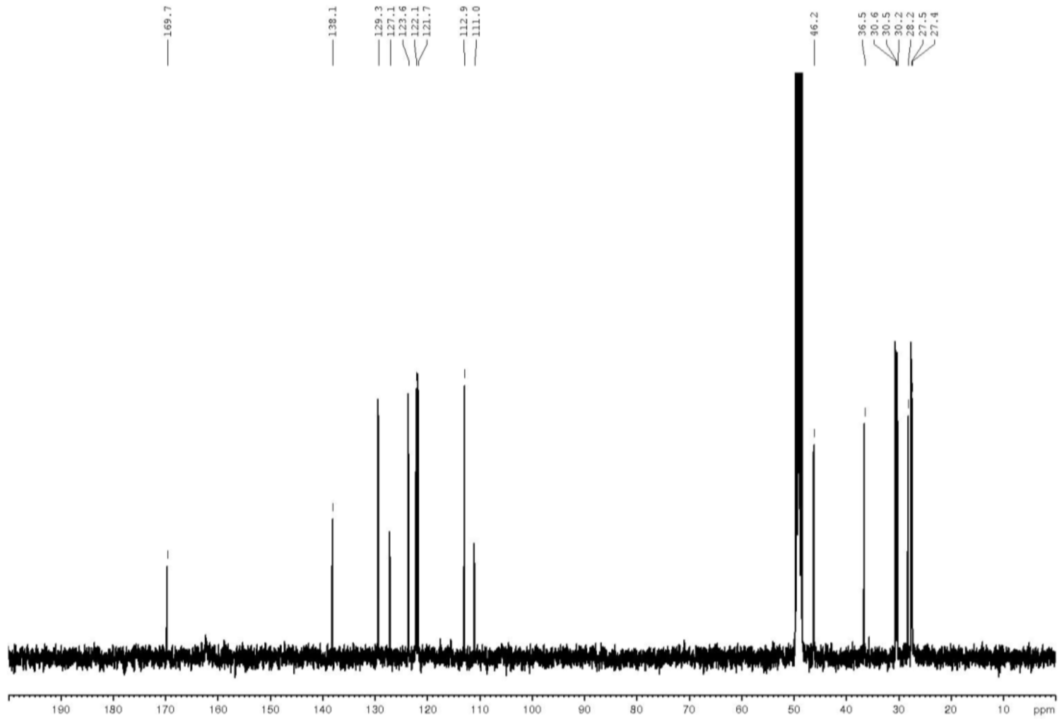

Figure S6 ^{1}H (CD$_3$OD, 400 MHz) and ^{13}C (CD$_3$OD, 100 MHz) NMR spectra for **12f**

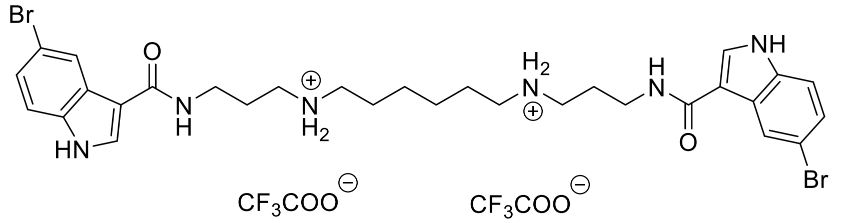

1H NMR (CD3OD, 400 MHz):

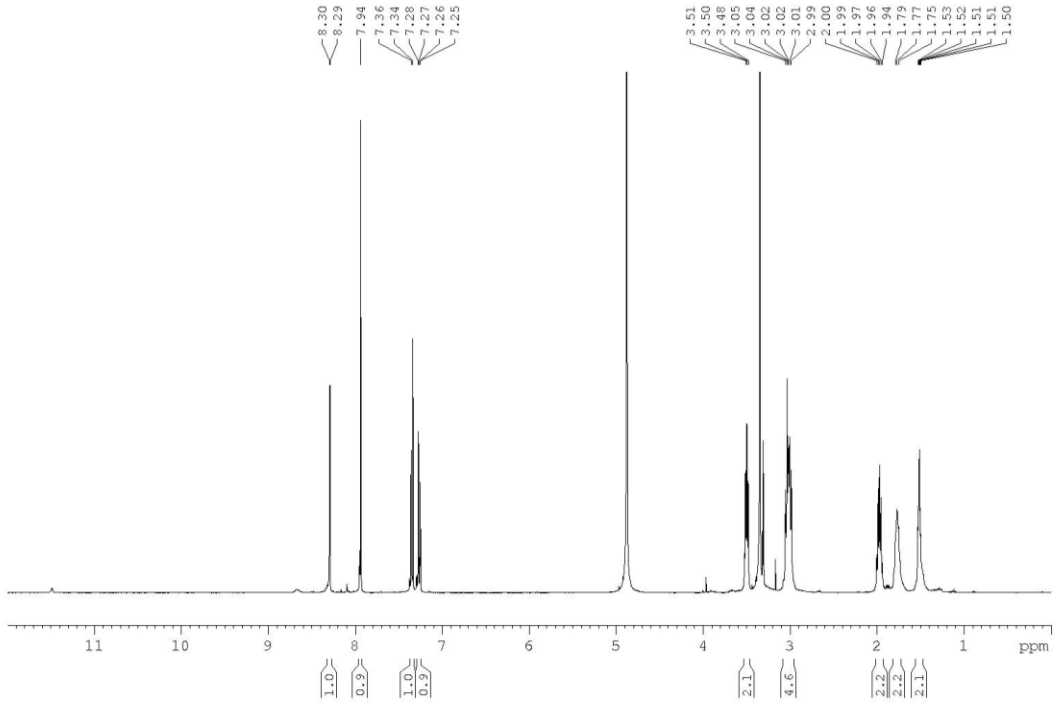

13C NMR (CD3OD, 100 MHz):

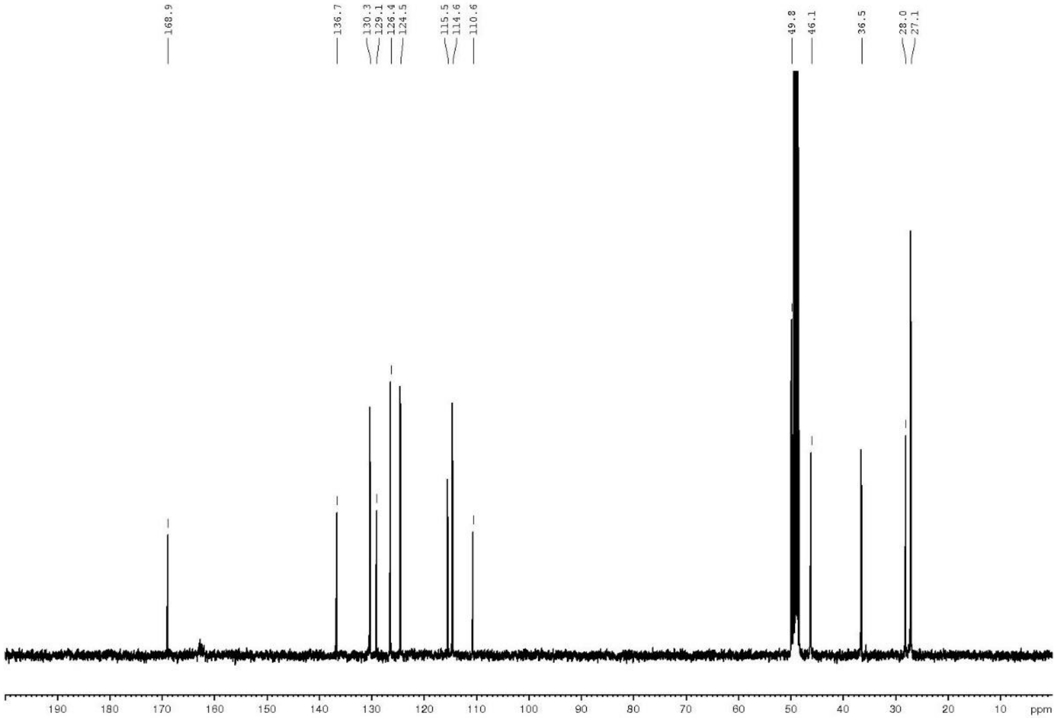

Figure S7 1H (CD3OD, 400 MHz) and 13C (CD3OD, 100 MHz) NMR spectra for **13b**

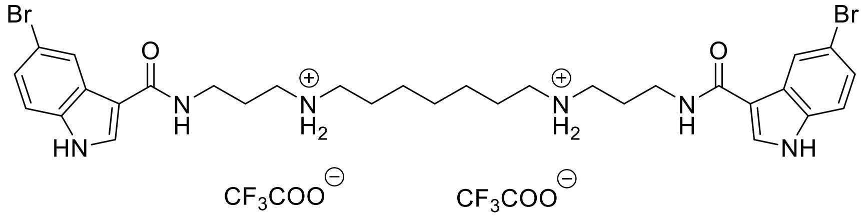

^1H NMR (CD_3OD, 400 MHz):

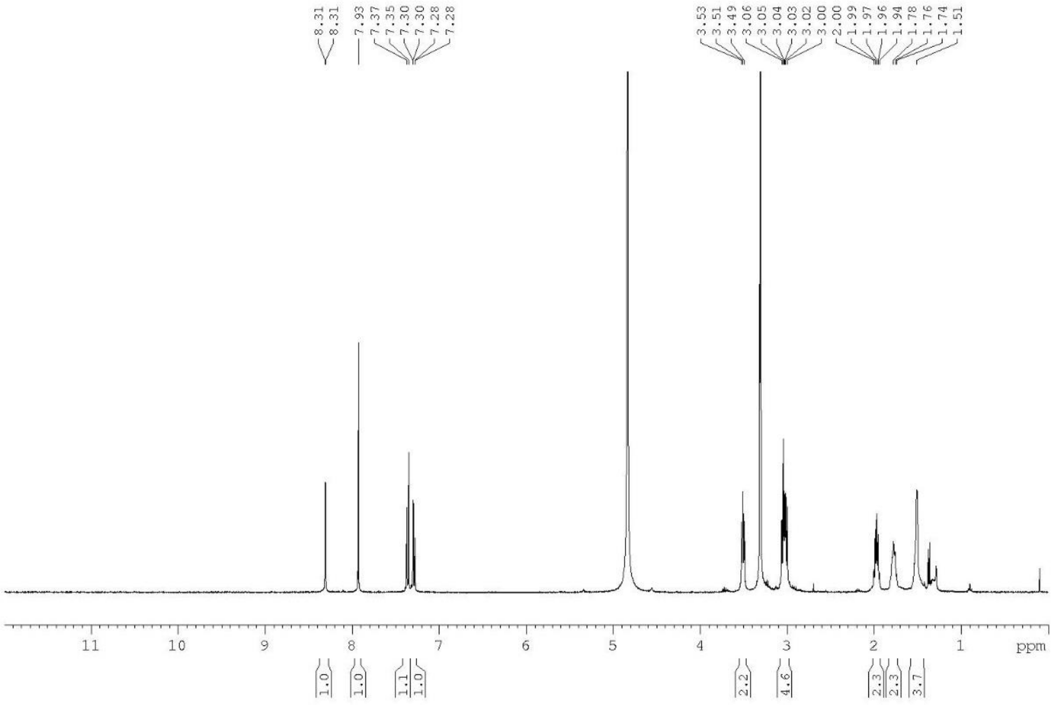

^{13}C NMR (CD_3OD, 100 MHz):

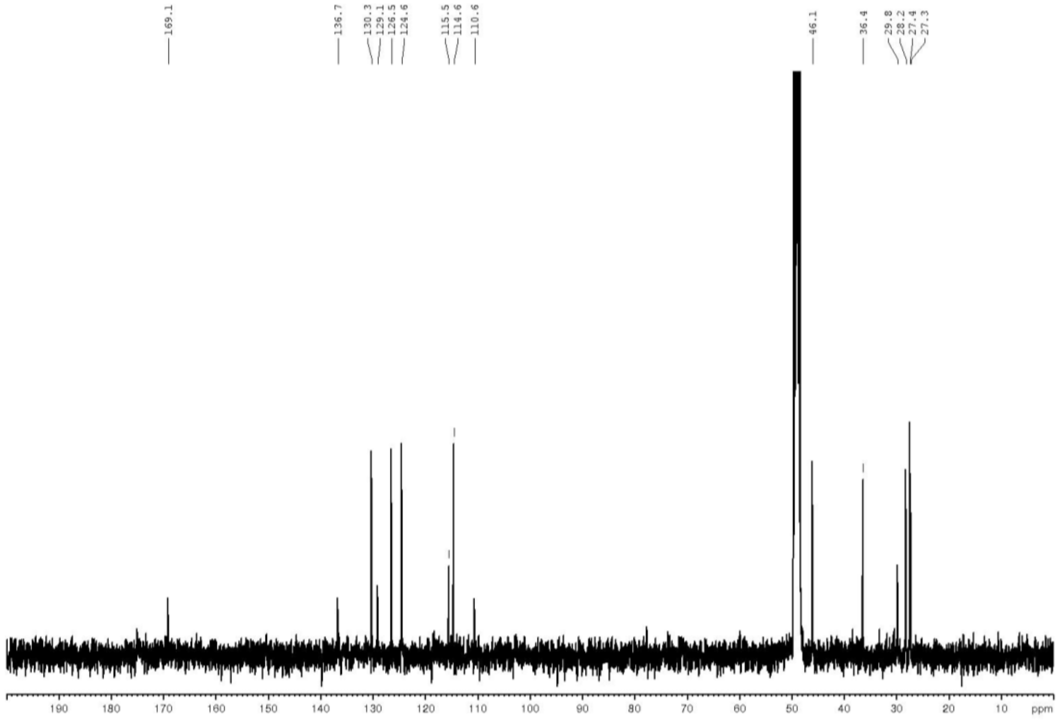

Figure S8 ^1H (CD_3OD, 400 MHz) and ^{13}C (CD_3OD, 100 MHz) NMR spectra for **13c**

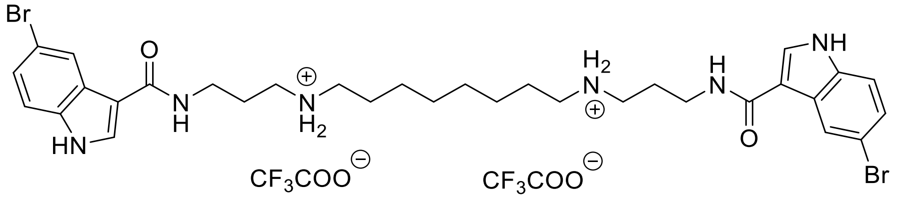

^{1}H NMR (CD_3OD, 400 MHz):

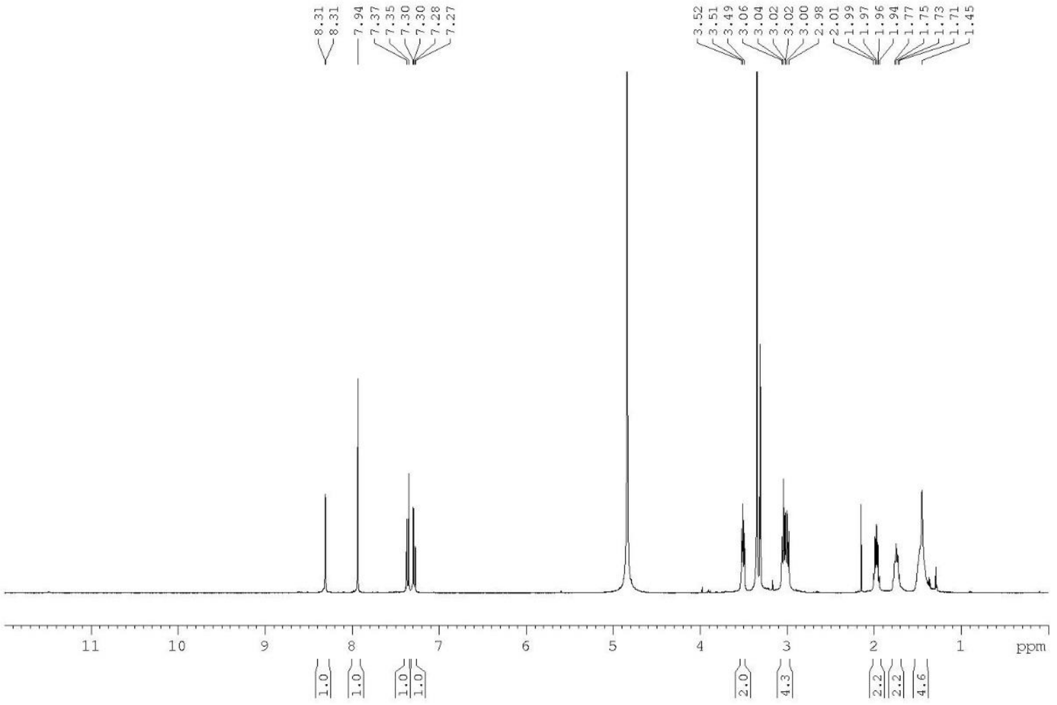

^{13}C NMR (CD_3OD, 100 MHz):

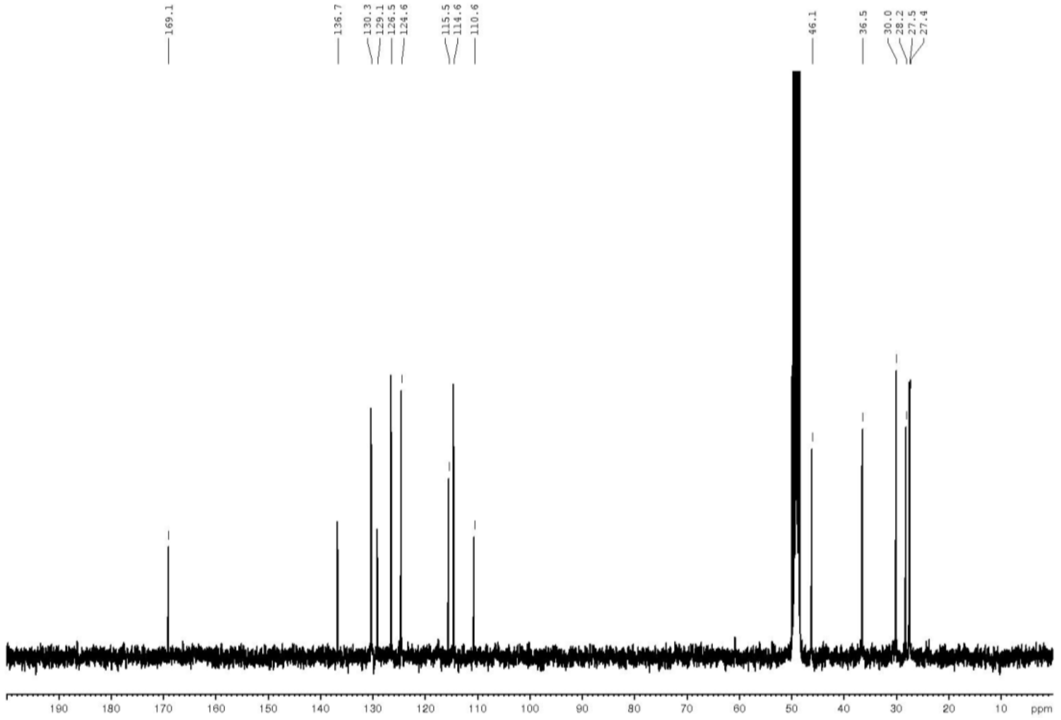

Figure S9 ^{1}H (CD_3OD, 400 MHz) and ^{13}C (CD_3OD, 100 MHz) NMR spectra for **13d**

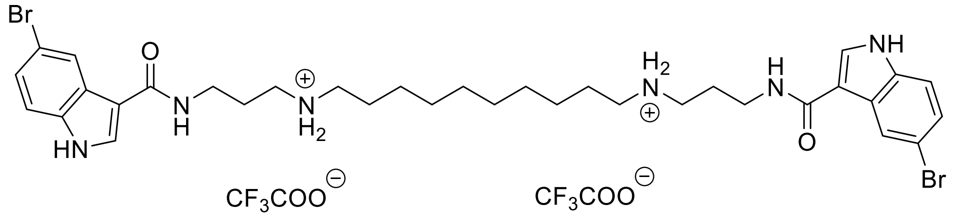

^{1}H NMR (CD_3OD, 400 MHz):

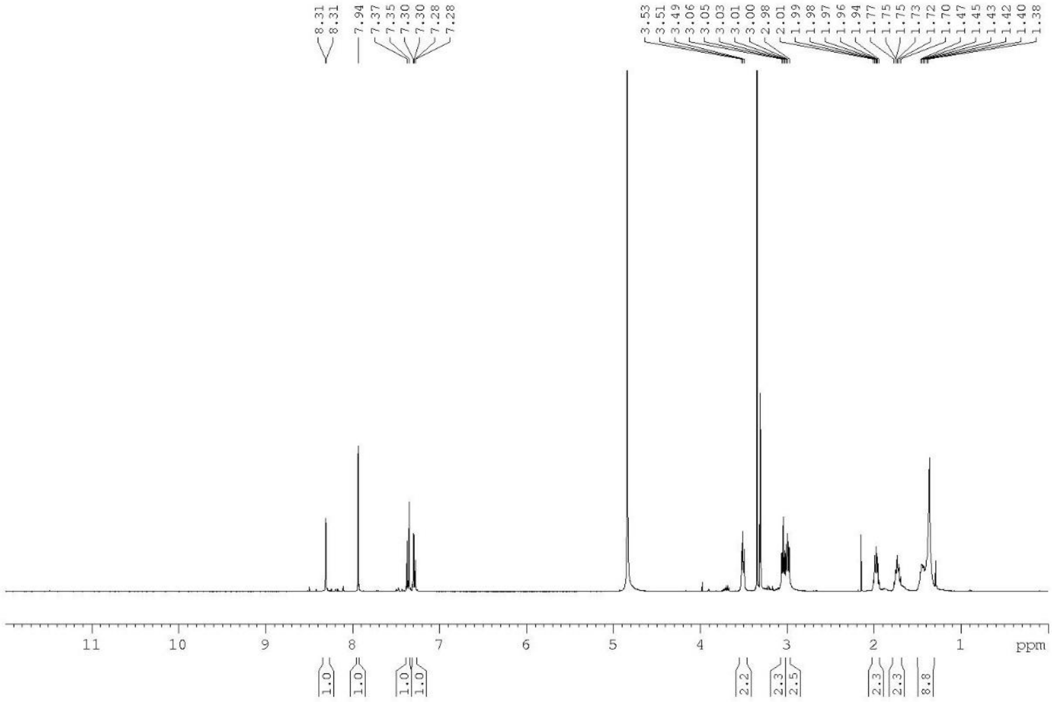

^{13}C NMR (CD_3OD, 100 MHz):

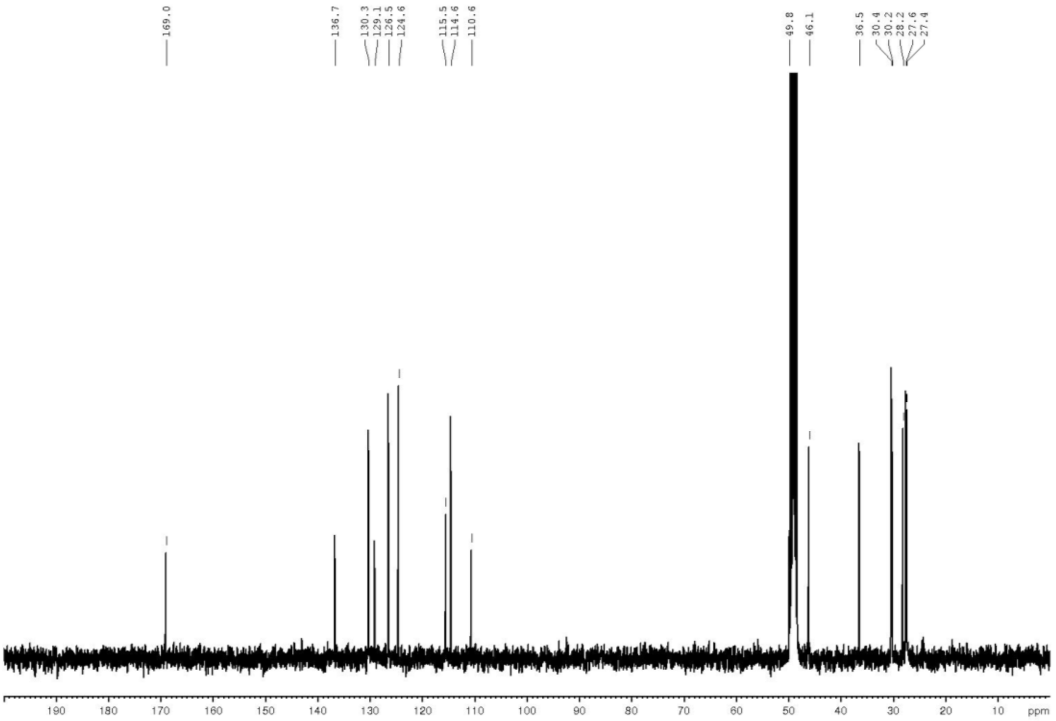

Figure S10 ^{1}H (CD_3OD, 400 MHz) and ^{13}C (CD_3OD, 100 MHz) NMR spectra for **13e**

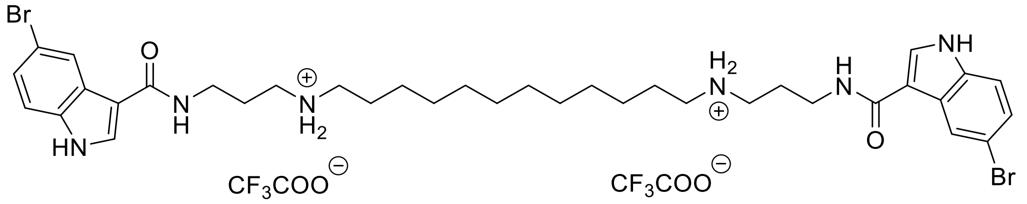

^{1}H NMR (CD_3OD, 400 MHz):

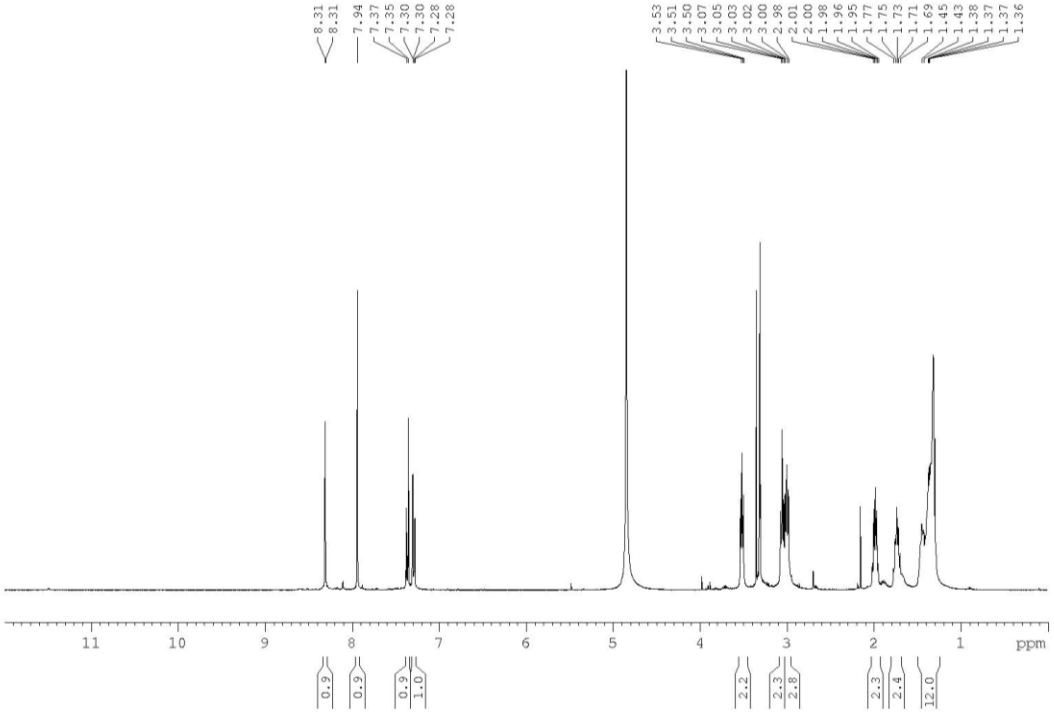

^{13}C NMR (CD_3OD, 100 MHz):

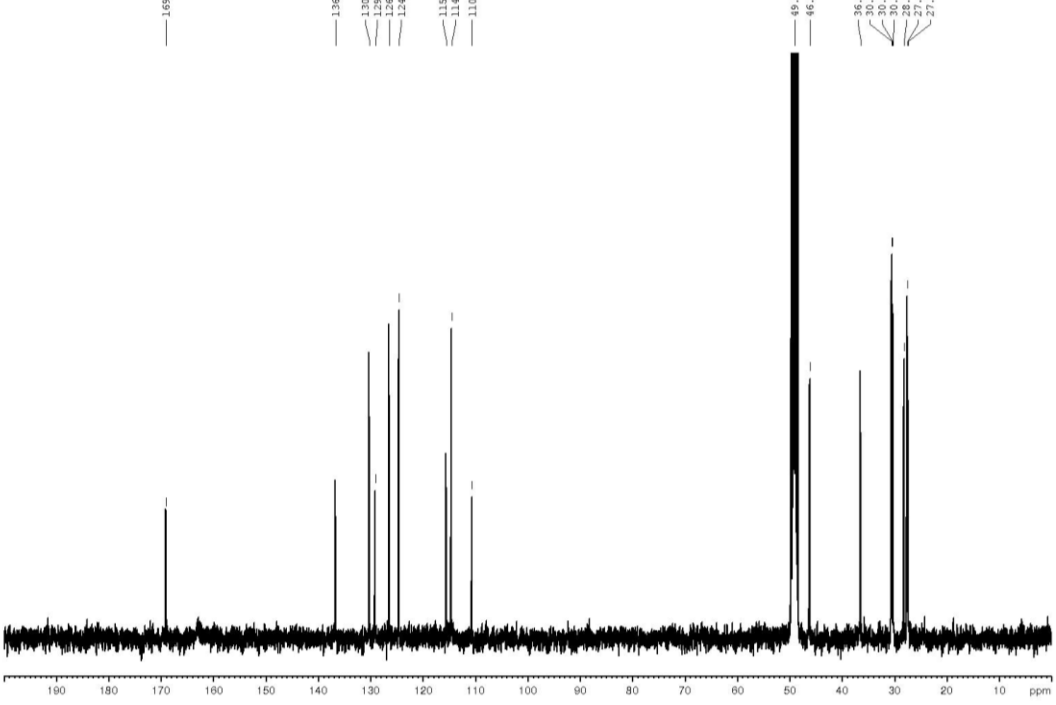

Figure S11 ^{1}H (CD_3OD, 400 MHz) and ^{13}C (CD_3OD, 100 MHz) NMR spectra for **13f**

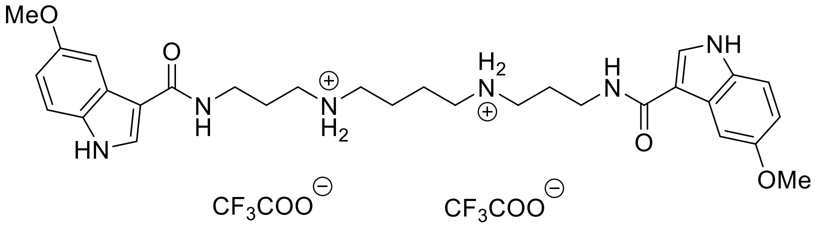

^{1}H NMR (CD_3OD, 400 MHz):

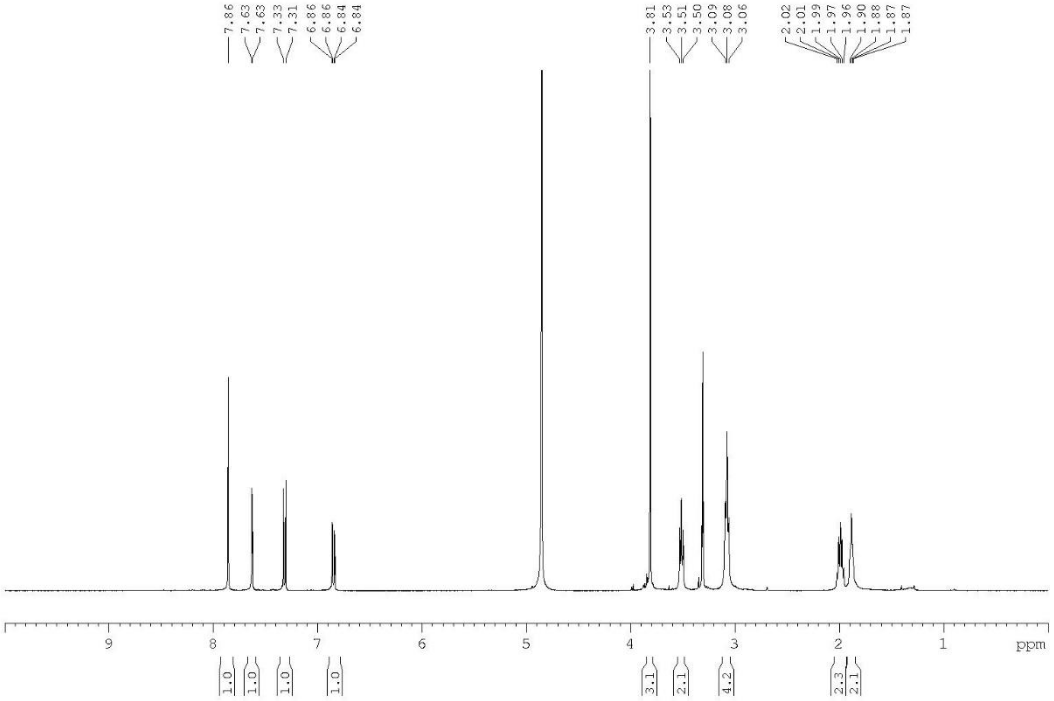

^{13}C NMR (CD_3OD, 100 MHz):

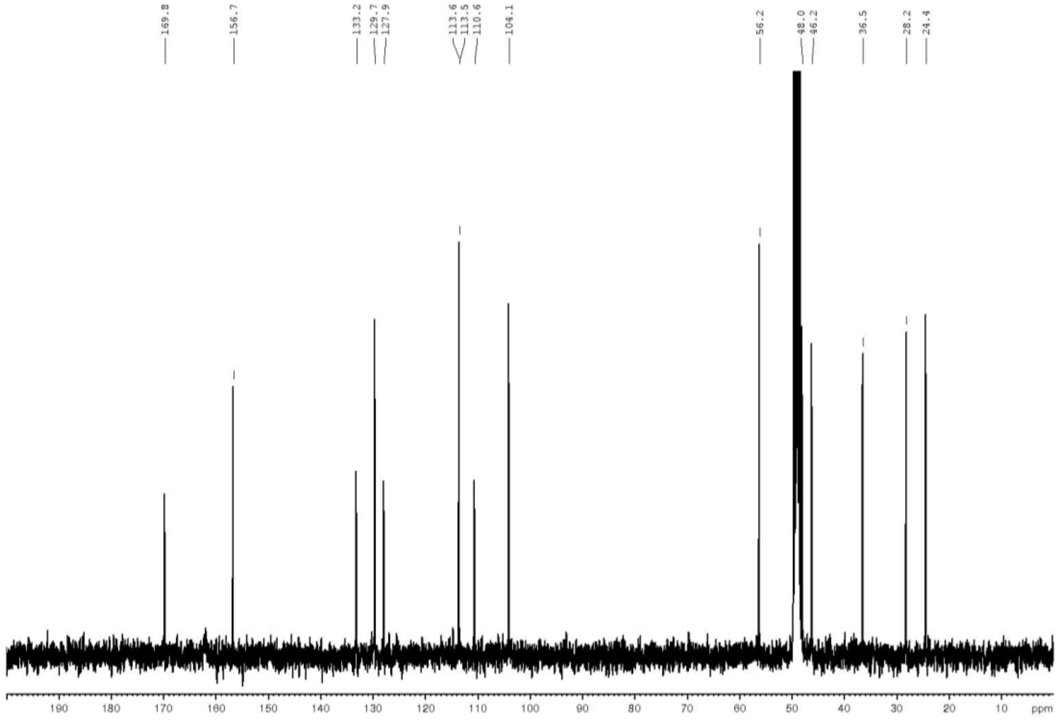

Figure S12 ^{1}H (CD_3OD, 400 MHz) and ^{13}C (CD_3OD, 100 MHz) NMR spectra for **14a**

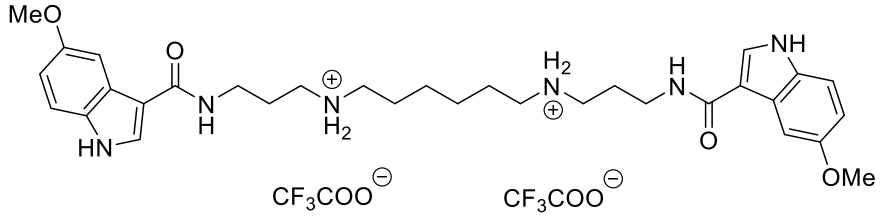

^{1}H NMR (CD_3OD, 400 MHz):

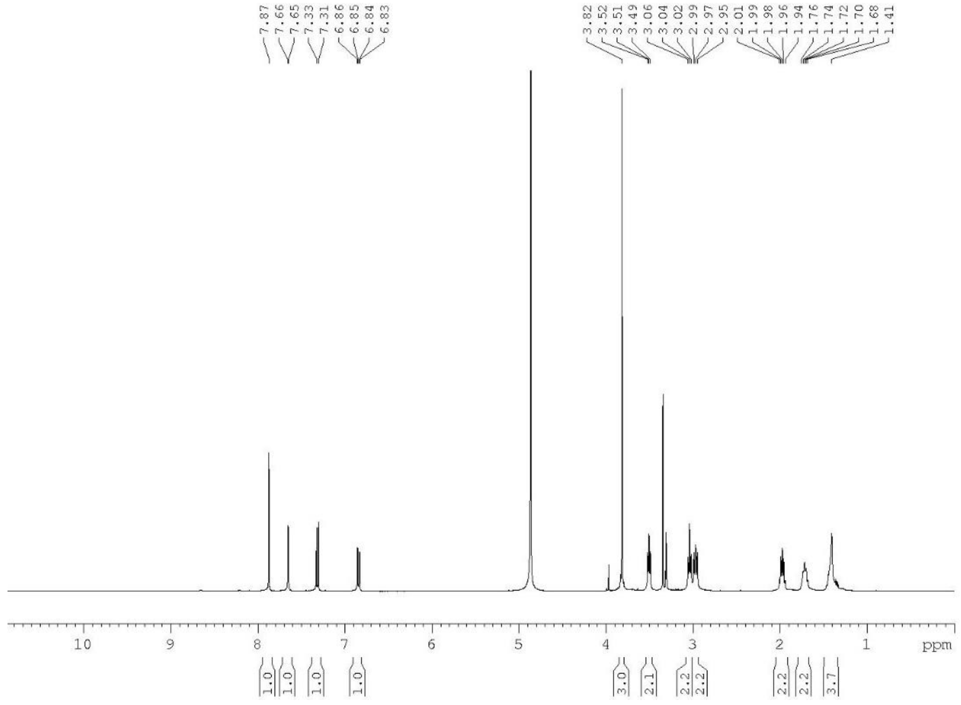

^{13}C NMR (CD_3OD, 100 MHz):

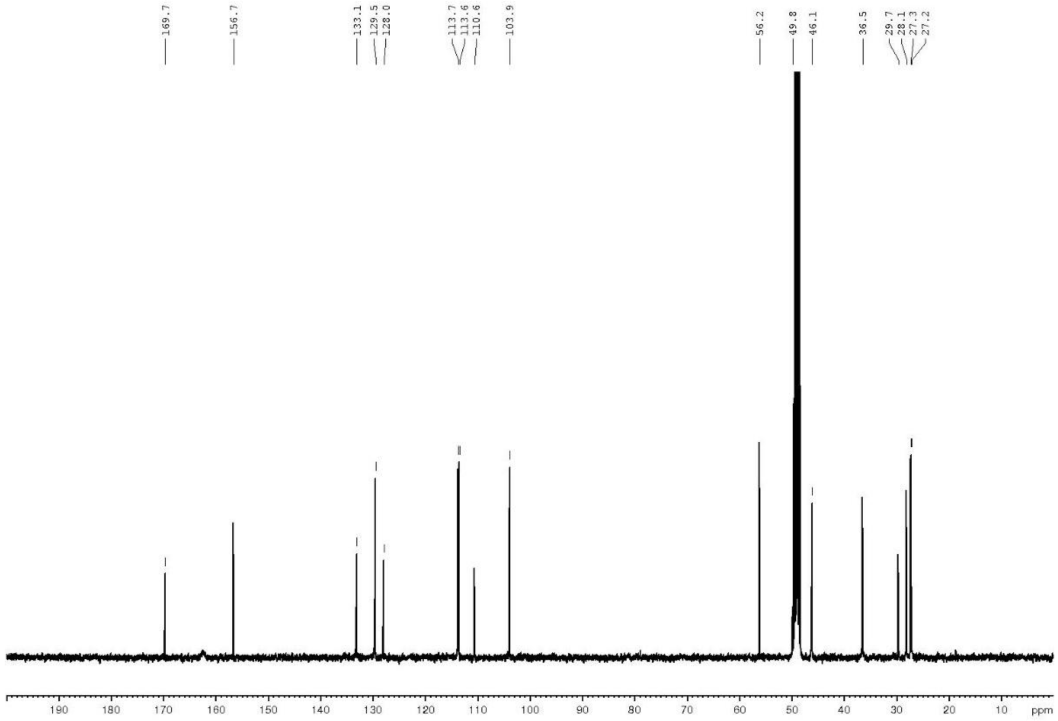

Figure S13 ^{1}H (CD_3OD, 400 MHz) and ^{13}C (CD_3OD, 100 MHz) NMR spectra for **14b**

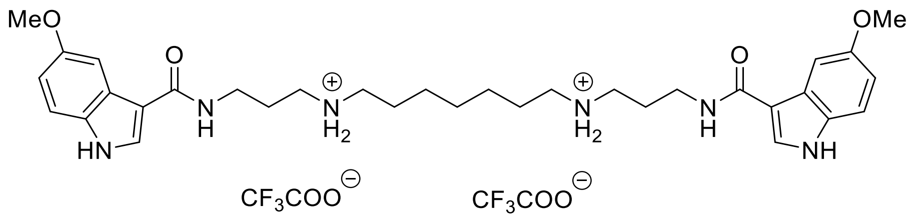

^{1}H NMR (CD_3OD, 400 MHz):

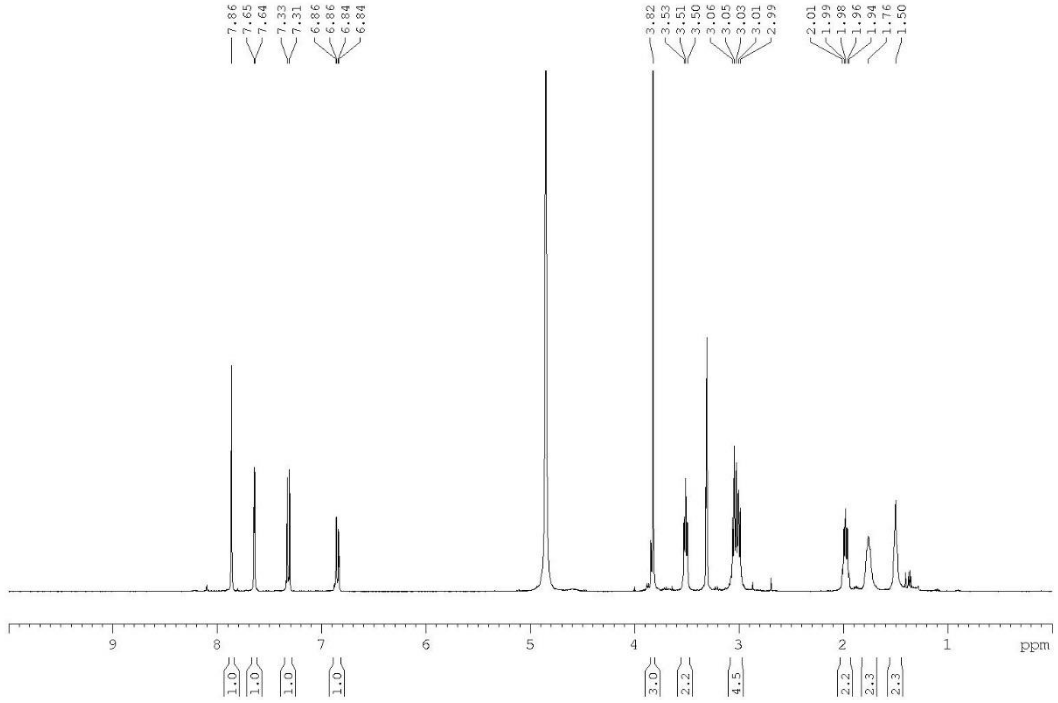

^{13}C NMR (CD_3OD, 100 MHz):

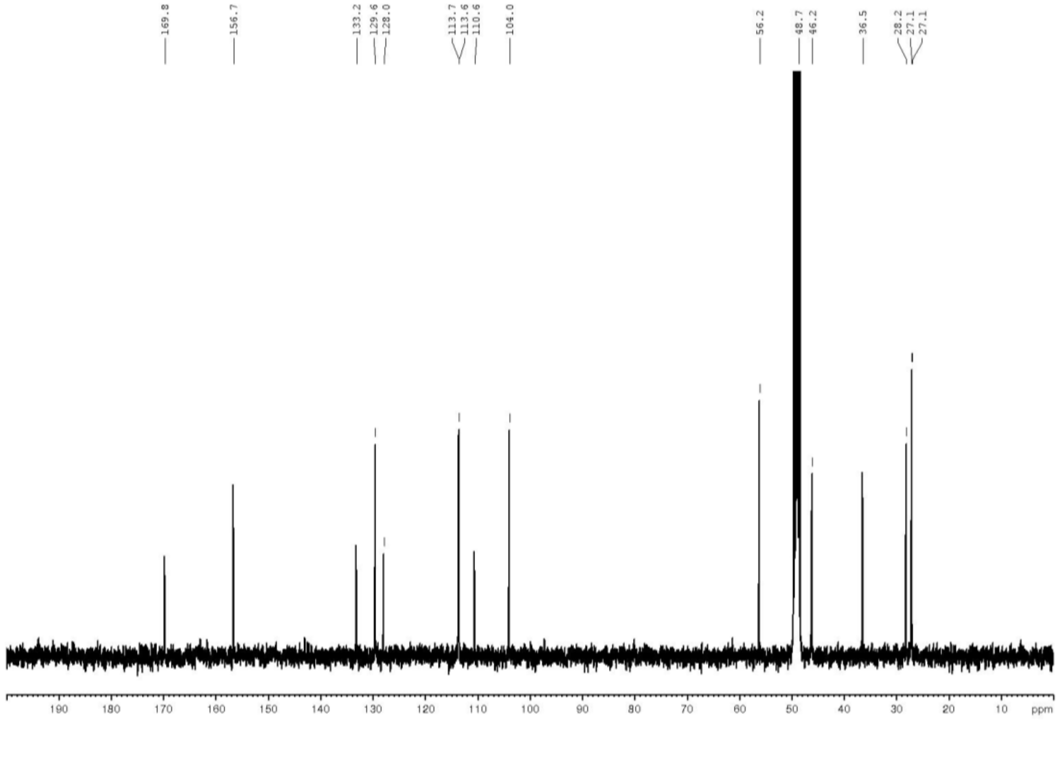

Figure S14 ^{1}H (CD_3OD, 400 MHz) and ^{13}C (CD_3OD, 100 MHz) NMR spectra for **14c**

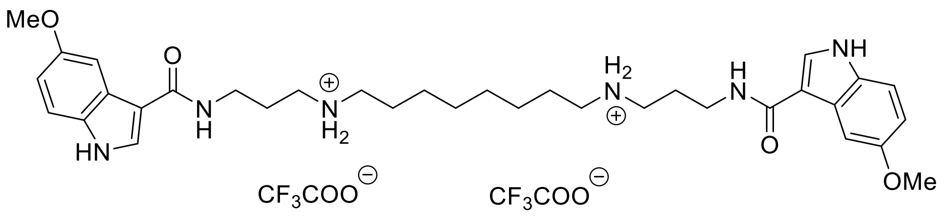

^{1}H NMR (CD_3OD, 400 MHz):

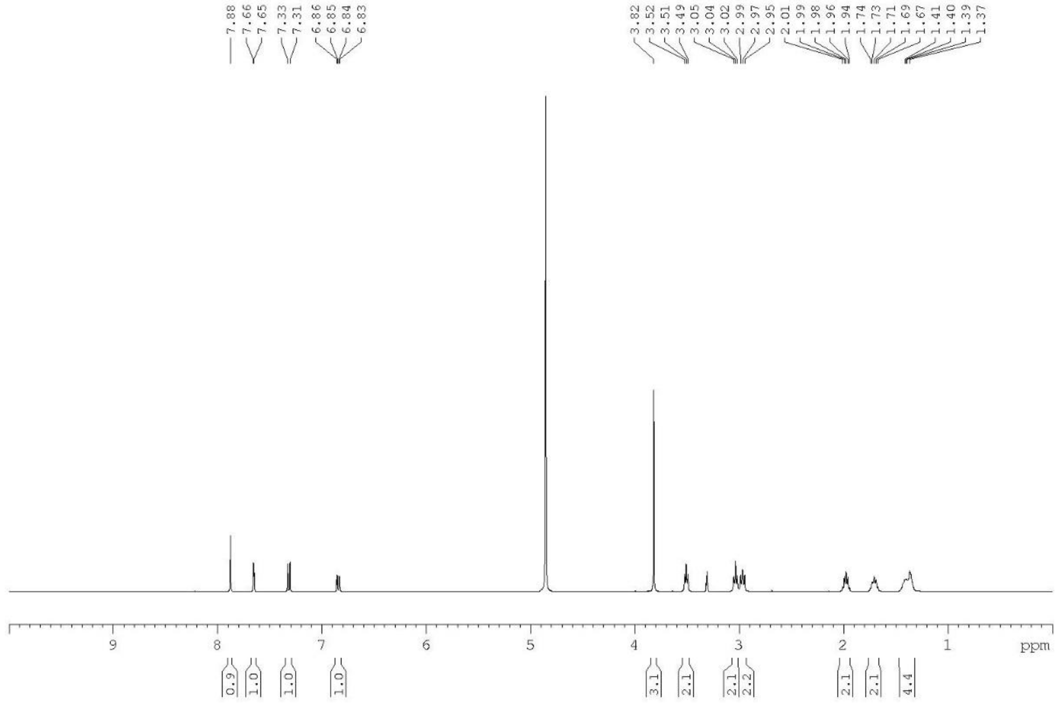

^{13}C NMR (CD_3OD, 100 MHz):

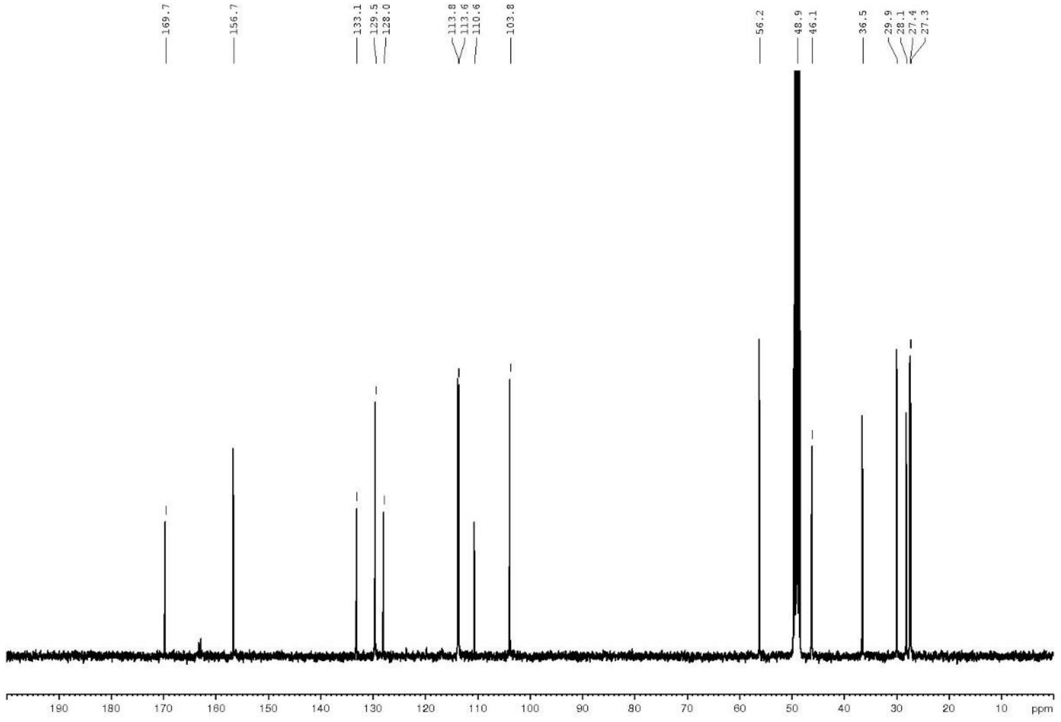

Figure S15 ^{1}H (CD_3OD, 400 MHz) and ^{13}C (CD_3OD, 100 MHz) NMR spectra for **14d**

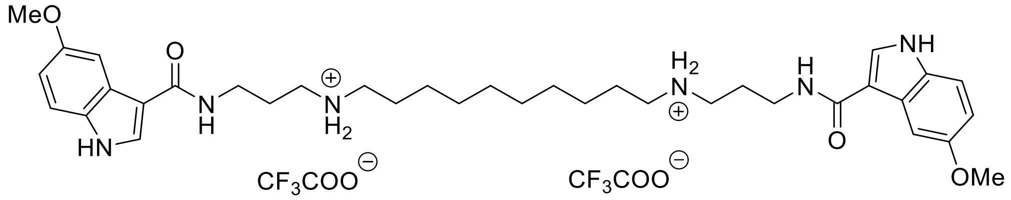

^{1}H NMR (CD_3OD, 400 MHz):

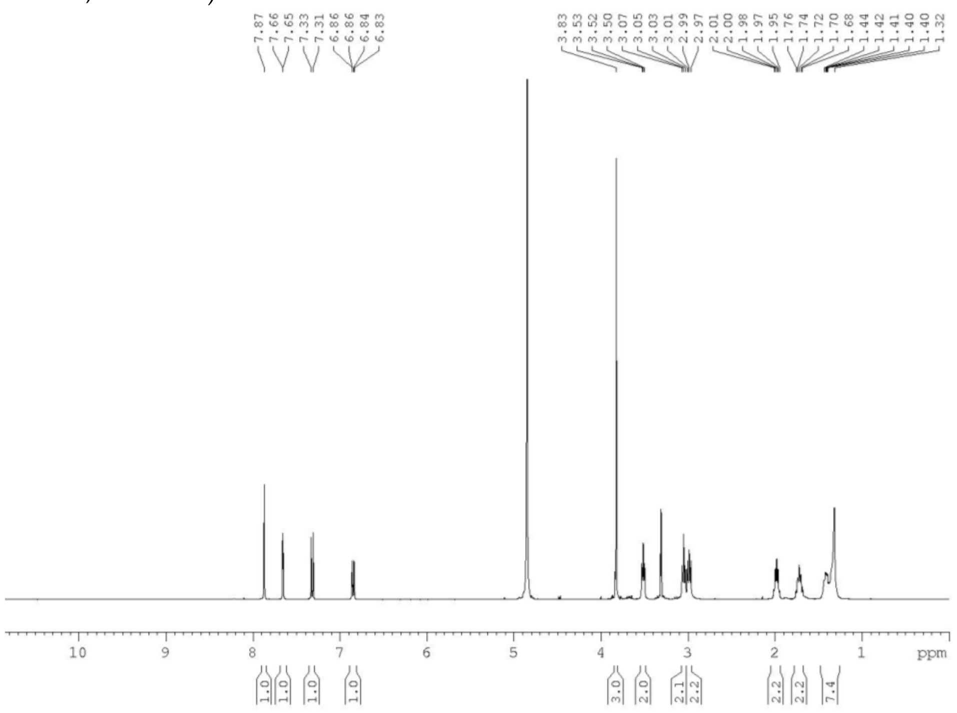

^{13}C NMR (CD_3OD, 100 MHz):

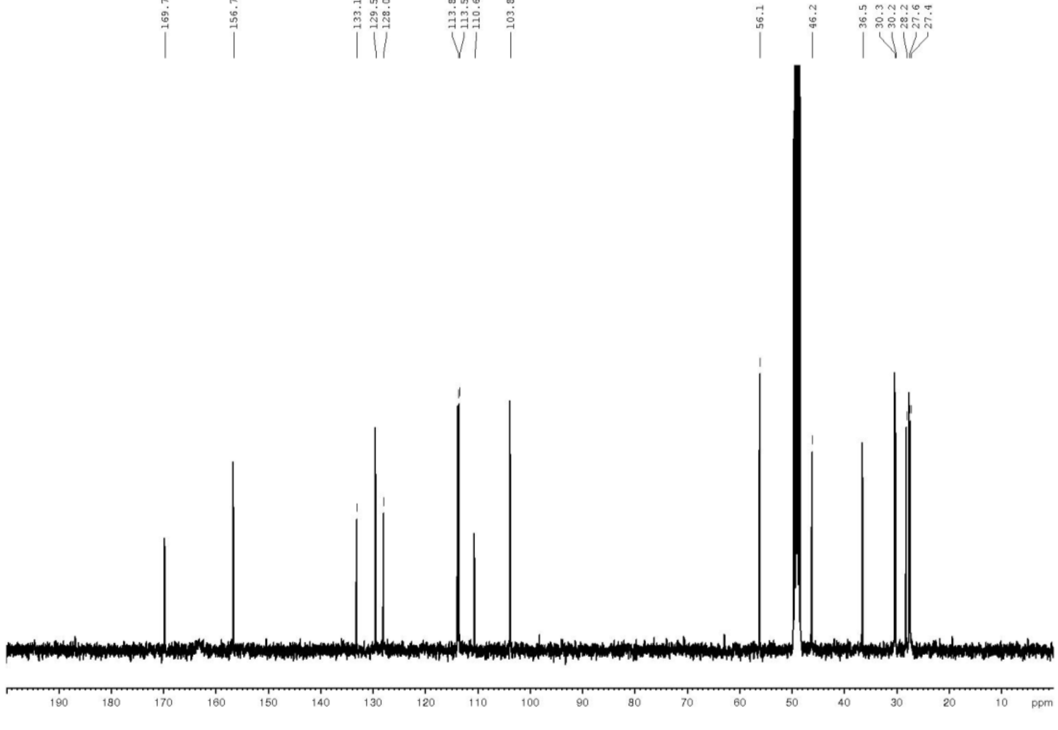

Figure S16 ^{1}H (CD_3OD, 400 MHz) and ^{13}C (CD_3OD, 100 MHz) NMR spectra for **14e**

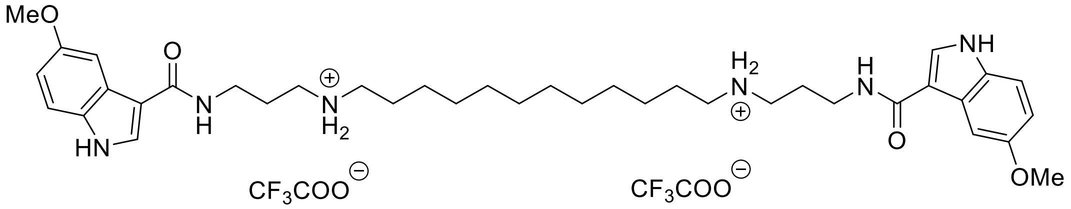

¹H NMR (CD₃OD, 400 MHz):

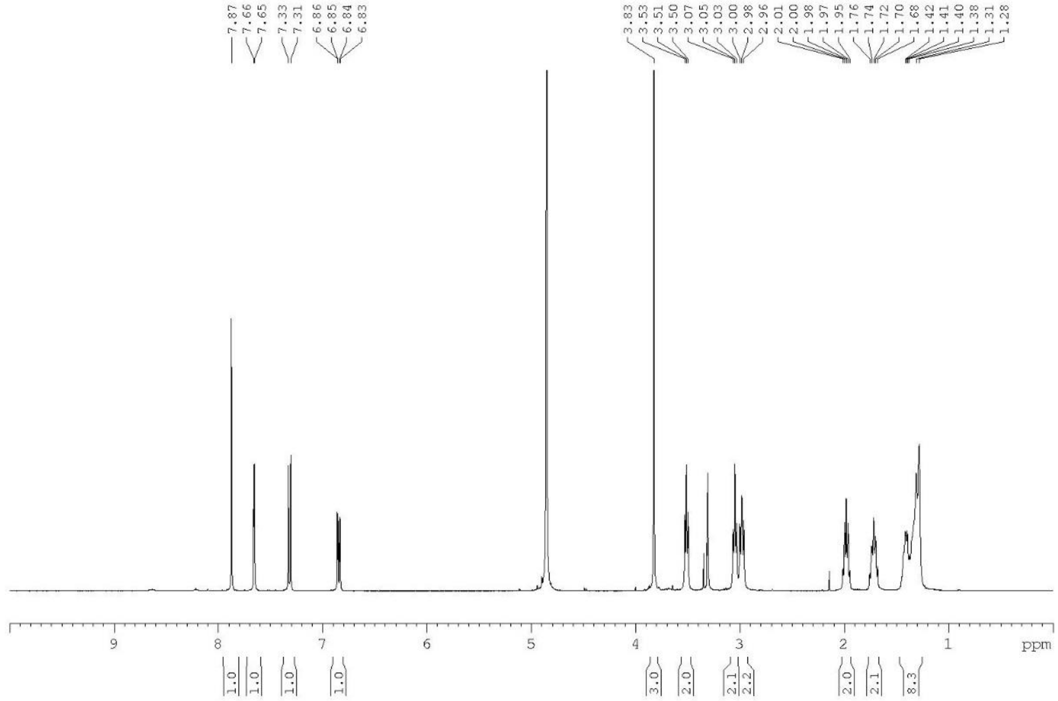

¹³C NMR (CD₃OD, 100 MHz):

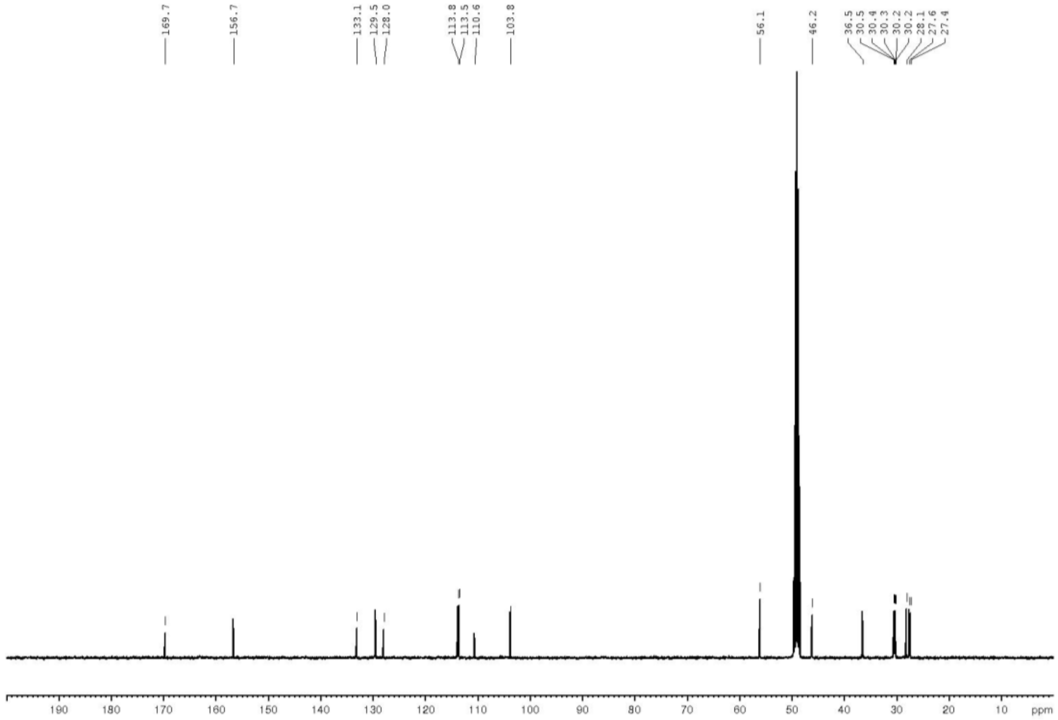

Figure S17 ¹H (CD₃OD, 400 MHz) and ¹³C (CD₃OD, 100 MHz) NMR spectra for **14f**

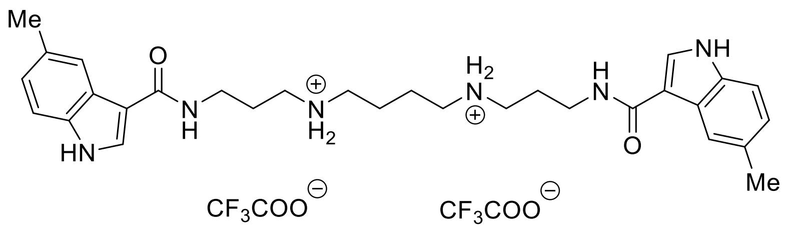

^{1}H NMR (CD3OD, 400 MHz):

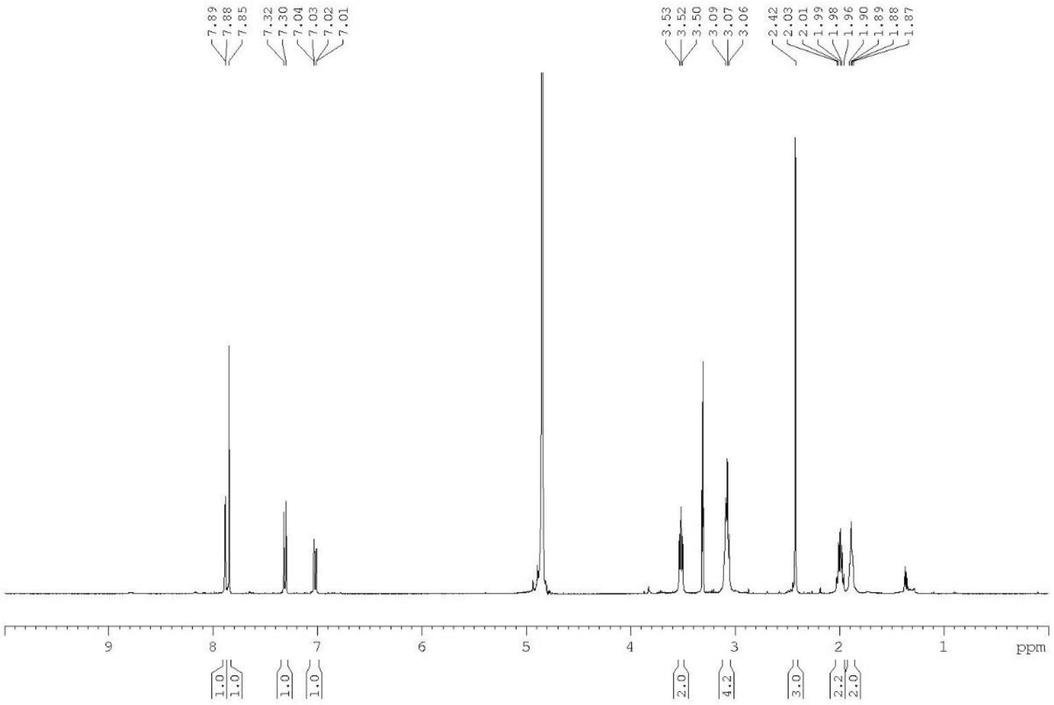

^{13}C NMR (CD3OD, 100 MHz):

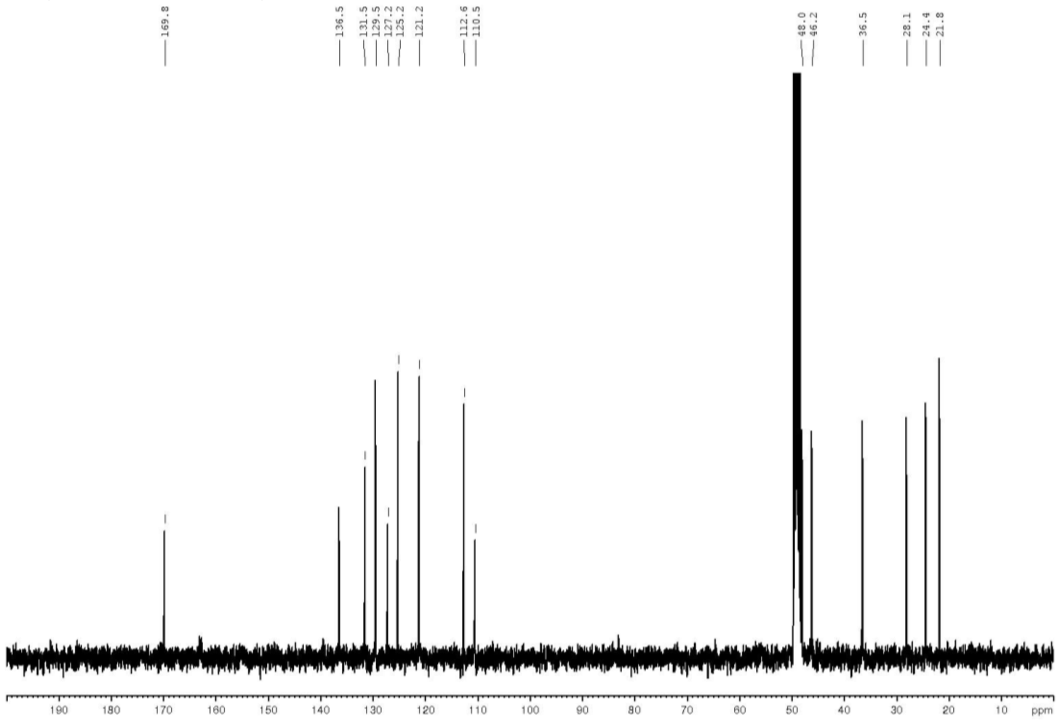

Figure S18 ^{1}H (CD3OD, 400 MHz) and ^{13}C (CD3OD, 100 MHz) NMR spectra for **15a**

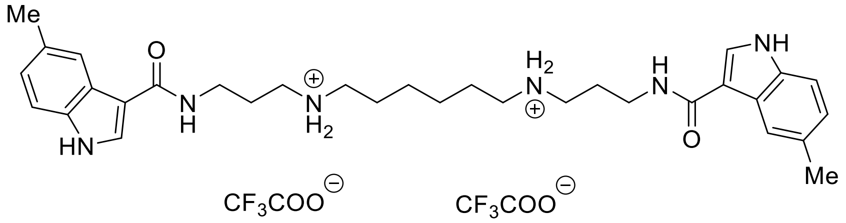

^{1}H NMR (CD_3OD, 400 MHz):

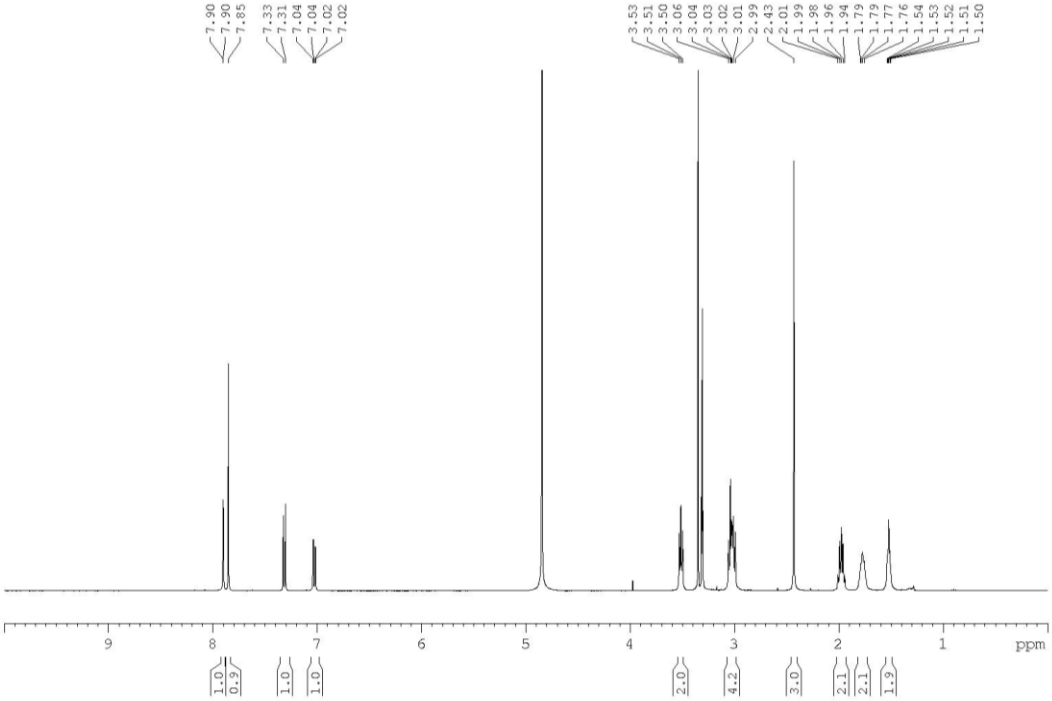

^{13}C NMR (CD_3OD, 100 MHz):

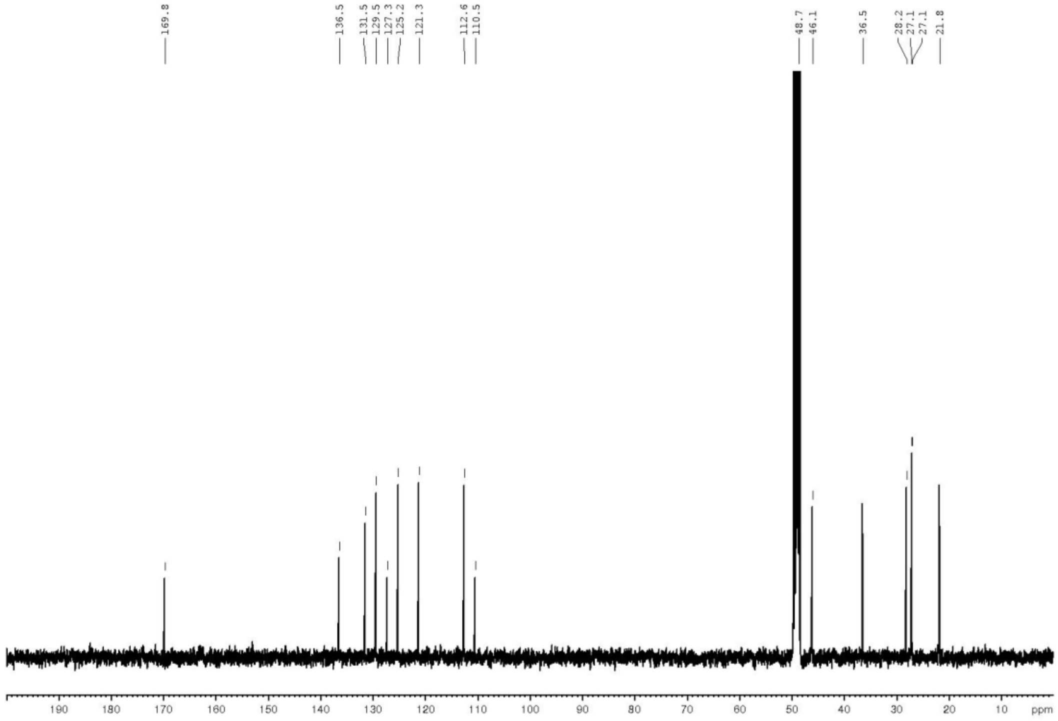

Figure S19 ^{1}H (CD_3OD, 400 MHz) and ^{13}C (CD_3OD, 100 MHz) NMR spectra for **15b**

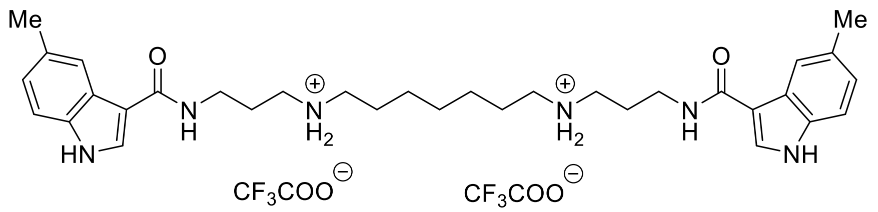

^{1}H NMR (CD_3OD, 400 MHz):

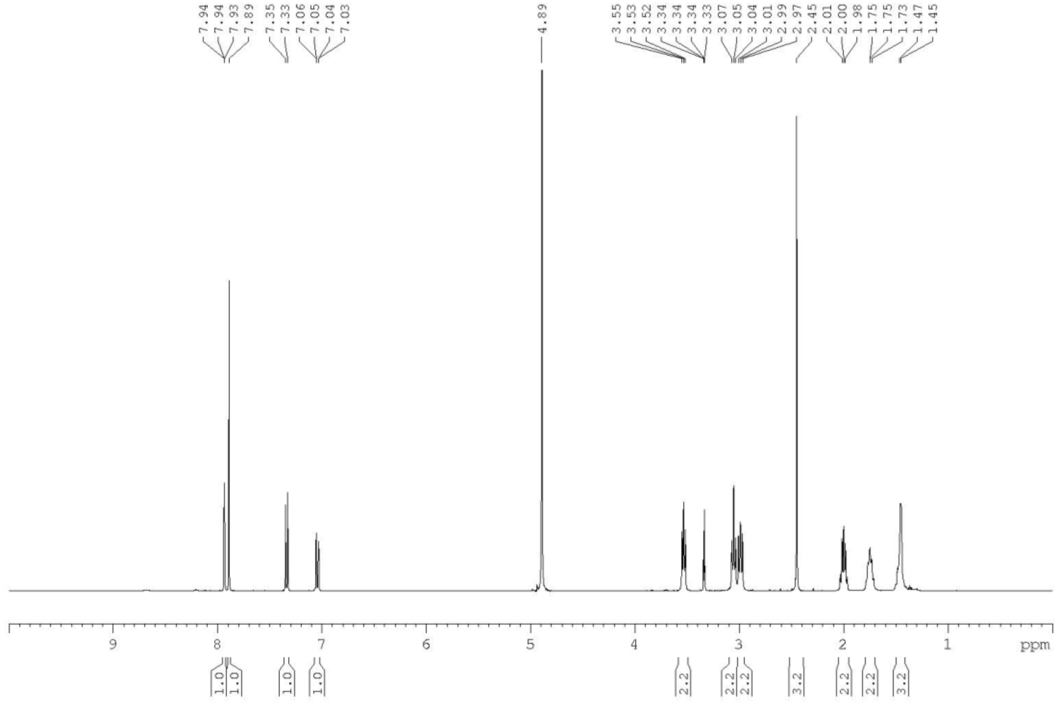

^{13}C NMR (CD_3OD, 100 MHz):

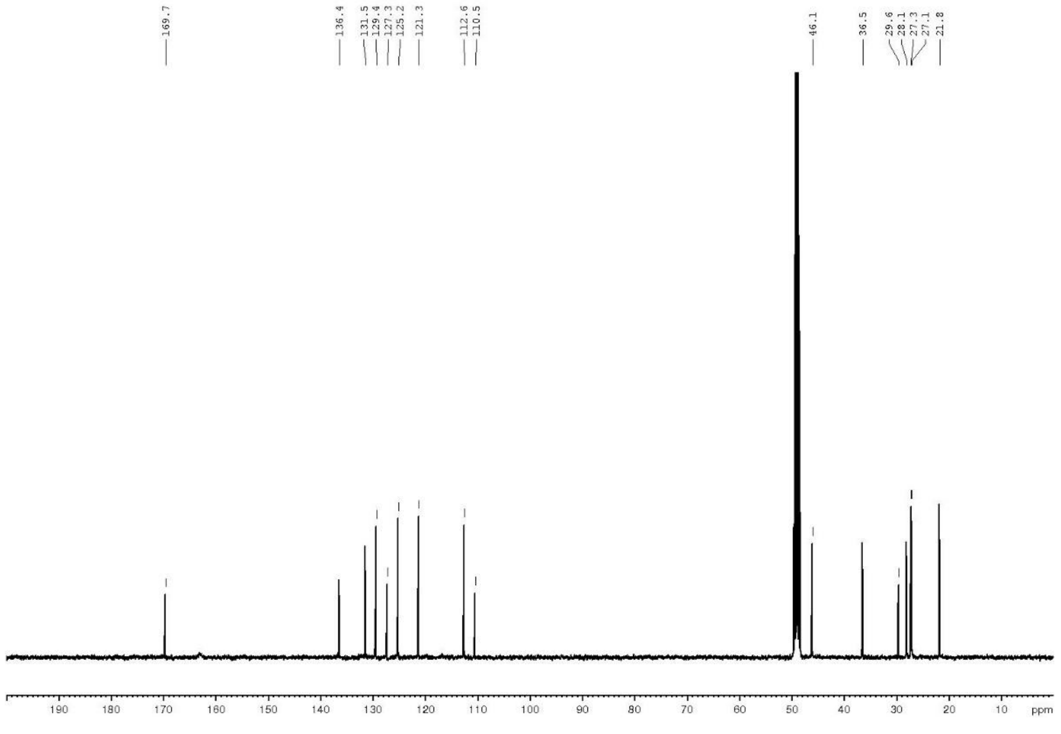

Figure S20 ^{1}H (CD_3OD, 400 MHz) and ^{13}C (CD_3OD, 100 MHz) NMR spectra for **15c**

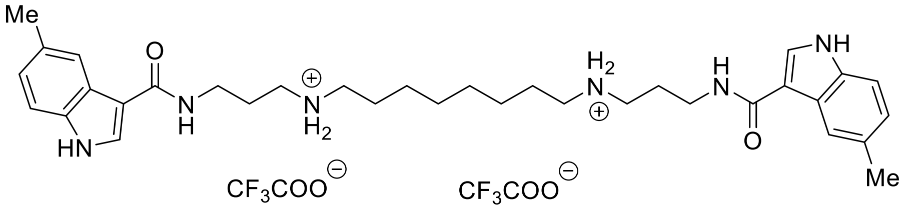

^{1}H NMR (CD_3OD, 400 MHz):

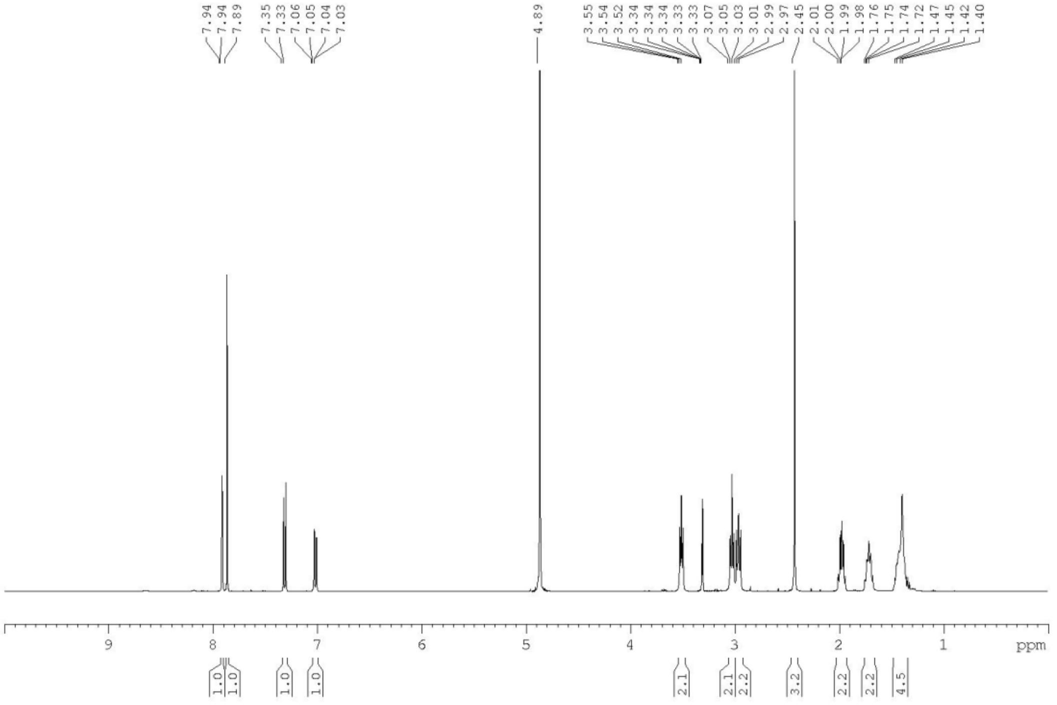

^{13}C NMR (CD_3OD, 100 MHz):

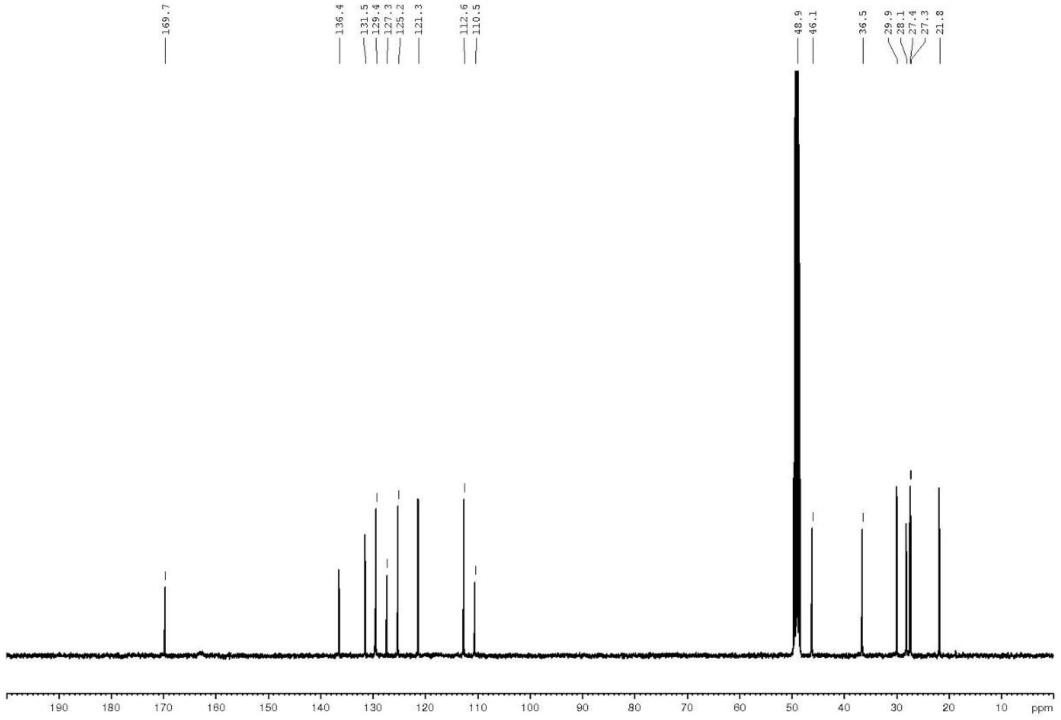

Figure S21 ^{1}H (CD_3OD, 400 MHz) and ^{13}C (CD_3OD, 100 MHz) NMR spectra for **15d**

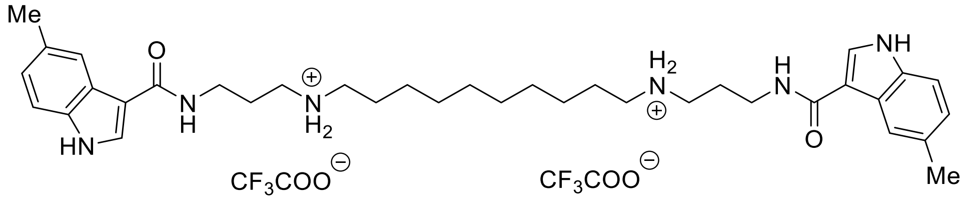

^{1}H NMR (CD_3OD, 400 MHz):

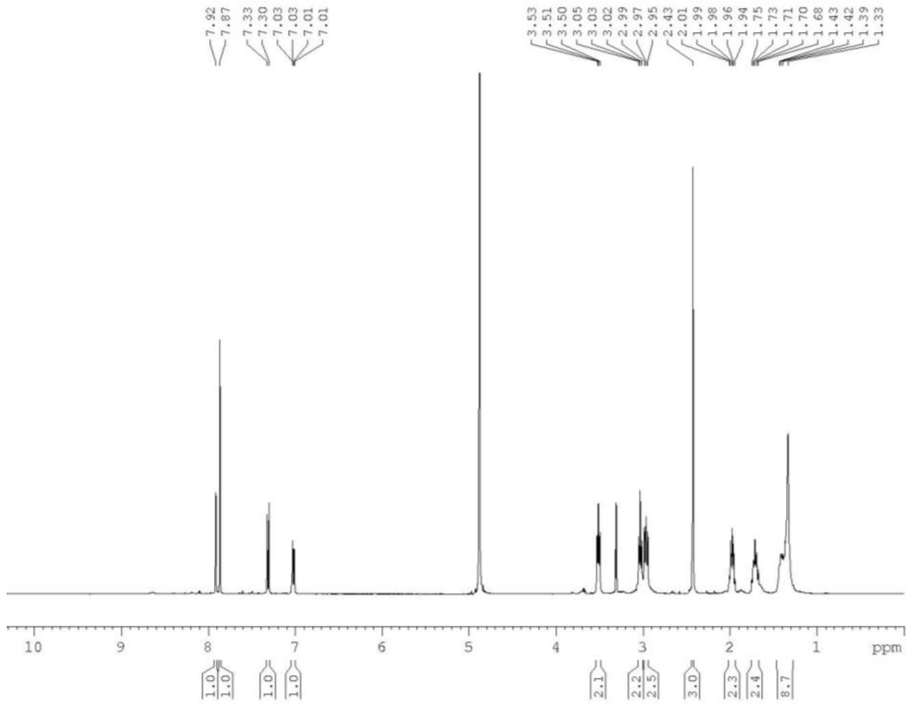

^{13}C NMR (CD_3OD, 100 MHz):

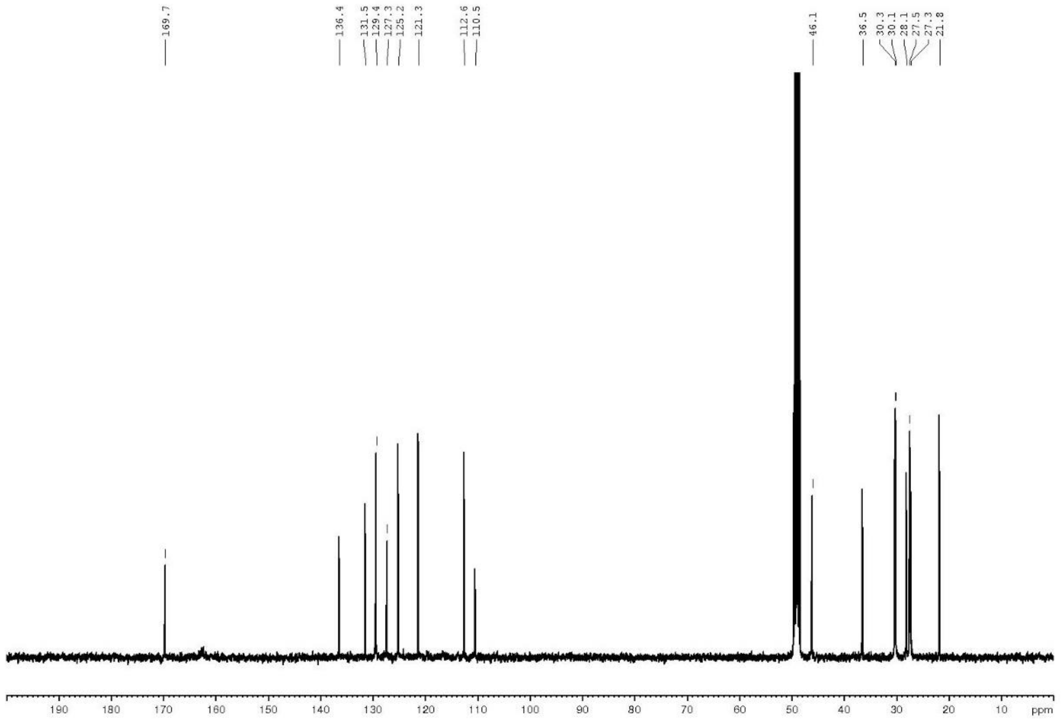

Figure S22 ^{1}H (CD_3OD, 400 MHz) and ^{13}C (CD_3OD, 100 MHz) NMR spectra for **15e**

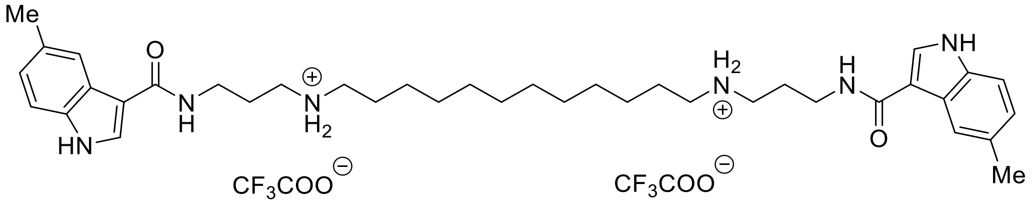

1H NMR (CD3OD, 400 MHz):

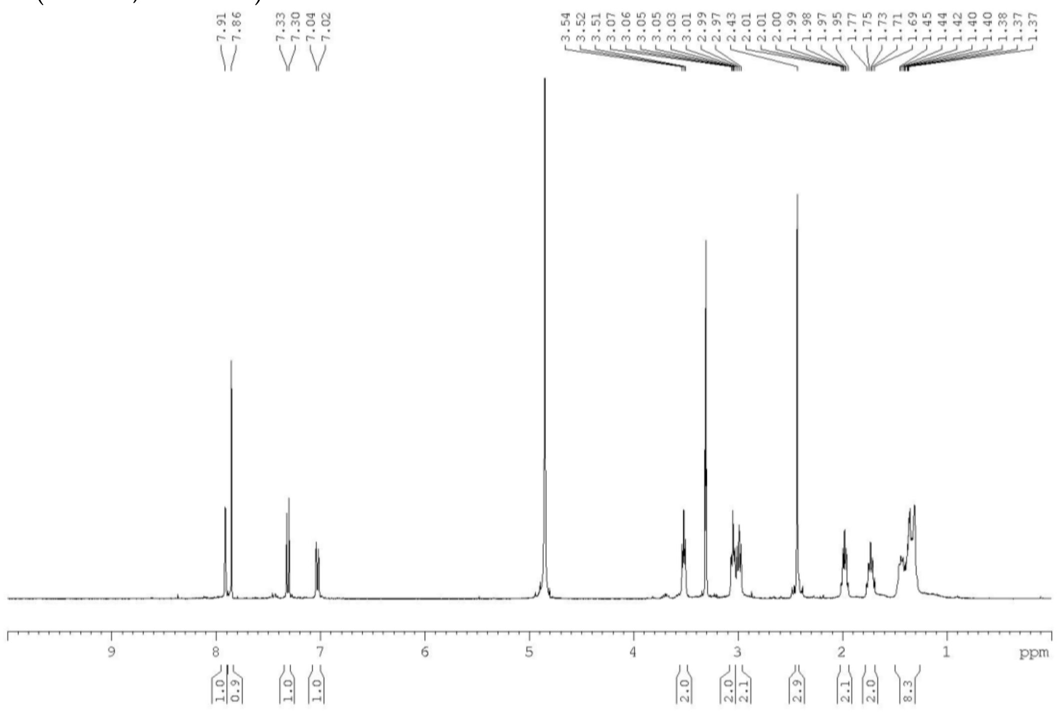

13C NMR (CD3OD, 100 MHz):

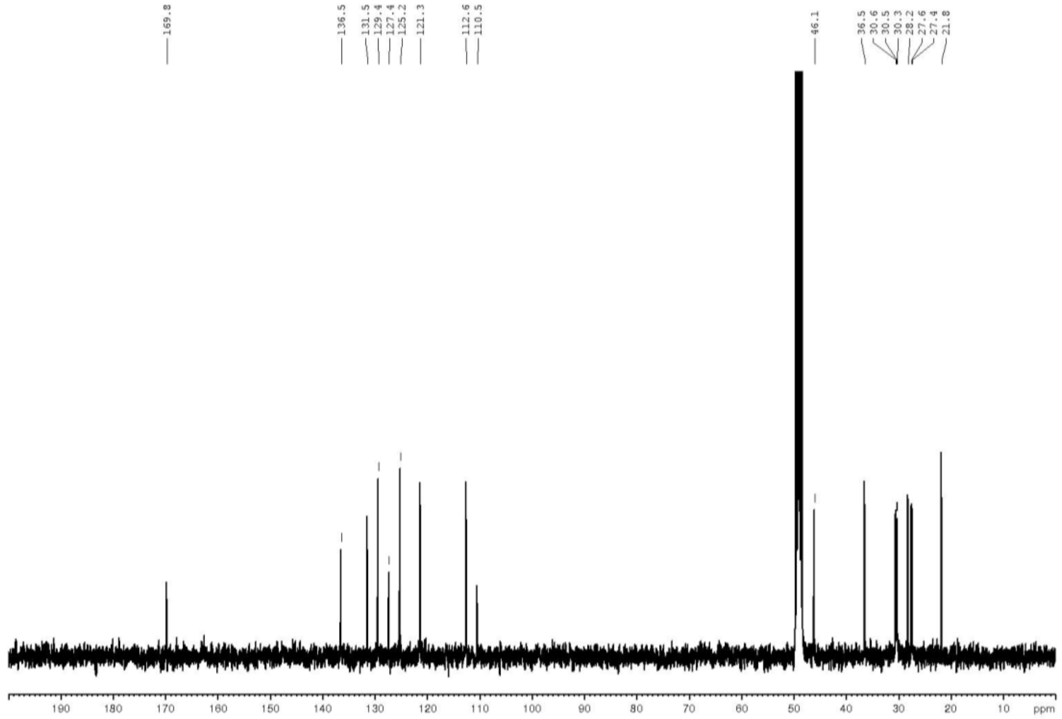

Figure S23 1H (CD3OD, 400 MHz) and 13C (CD3OD, 100 MHz) NMR spectra for **15f**

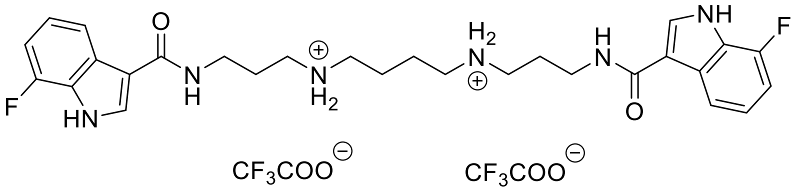

^{1}H NMR (CD3OD, 400 MHz):

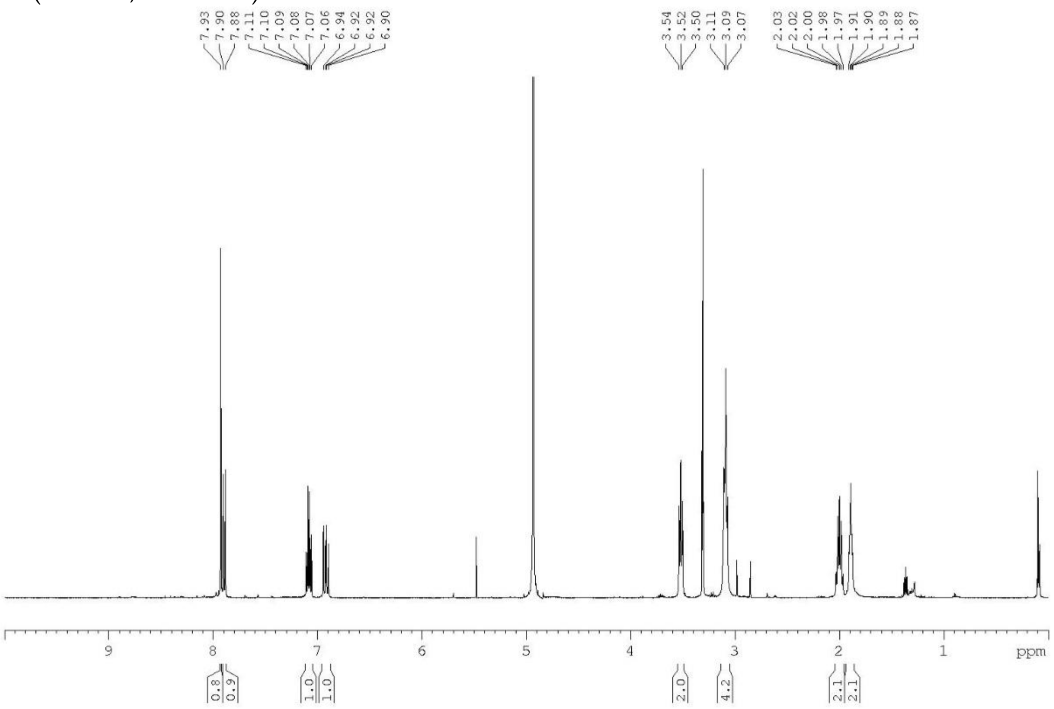

^{13}C NMR (CD3OD, 100 MHz):

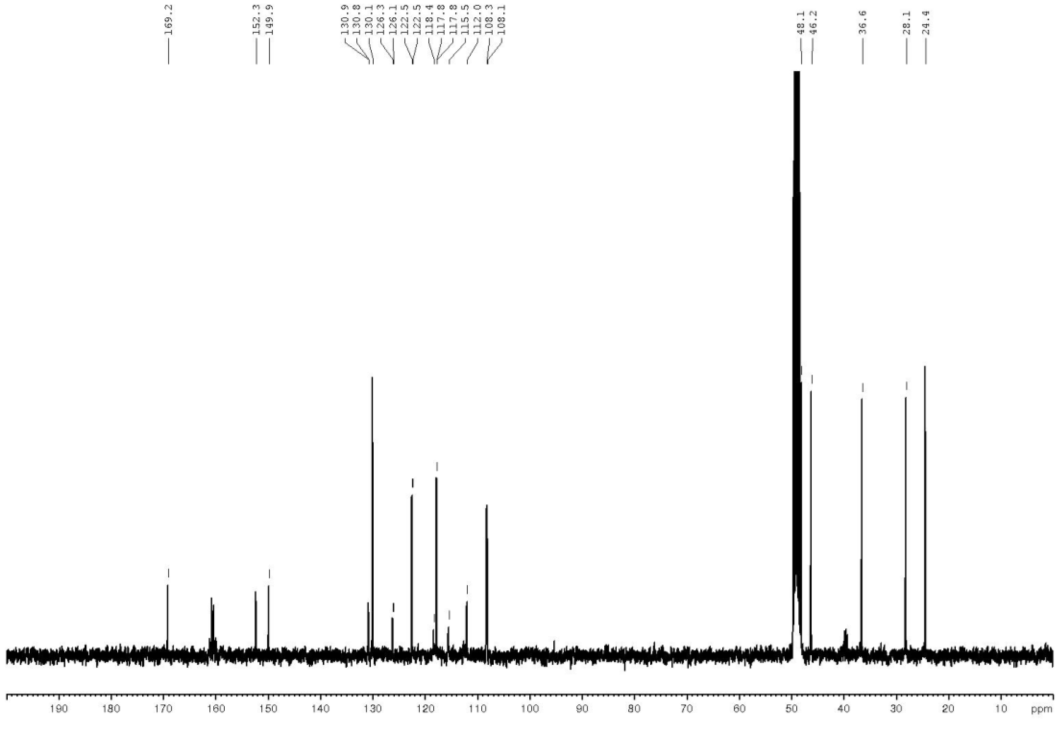

Figure S24 ^{1}H (CD3OD, 400 MHz) and ^{13}C (CD3OD, 100 MHz) NMR spectra for **16a**

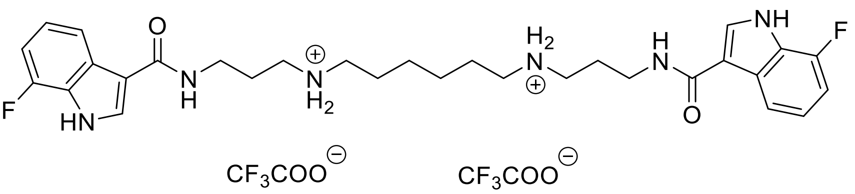

^{1}H NMR (CD3OD, 400 MHz):

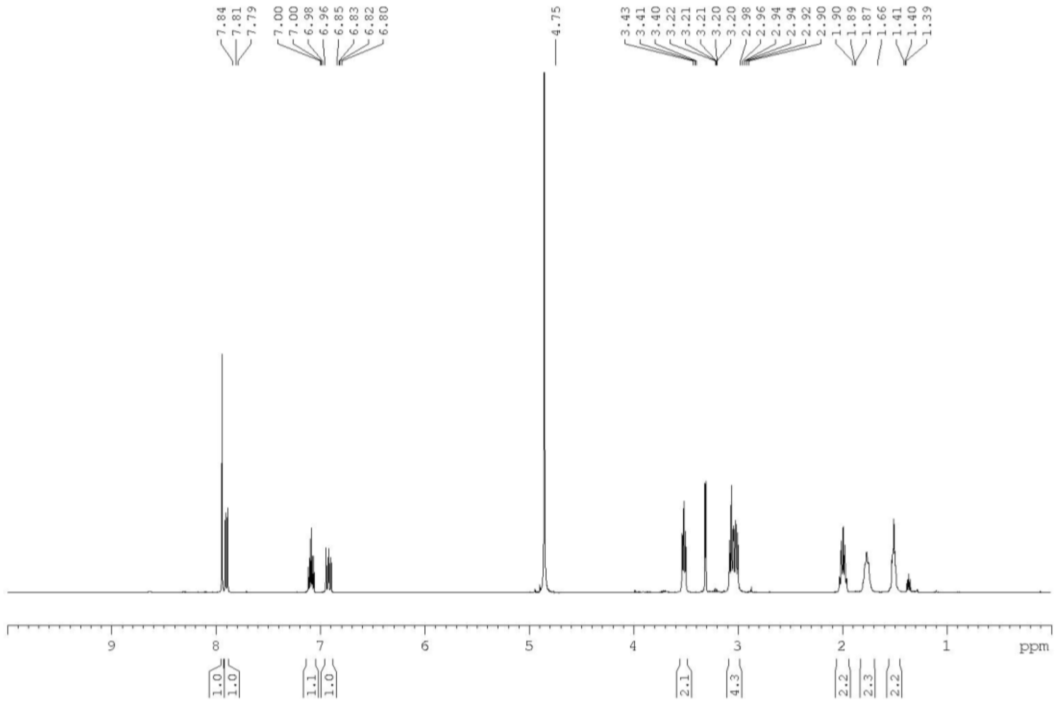

^{13}C NMR (CD3OD, 100 MHz):

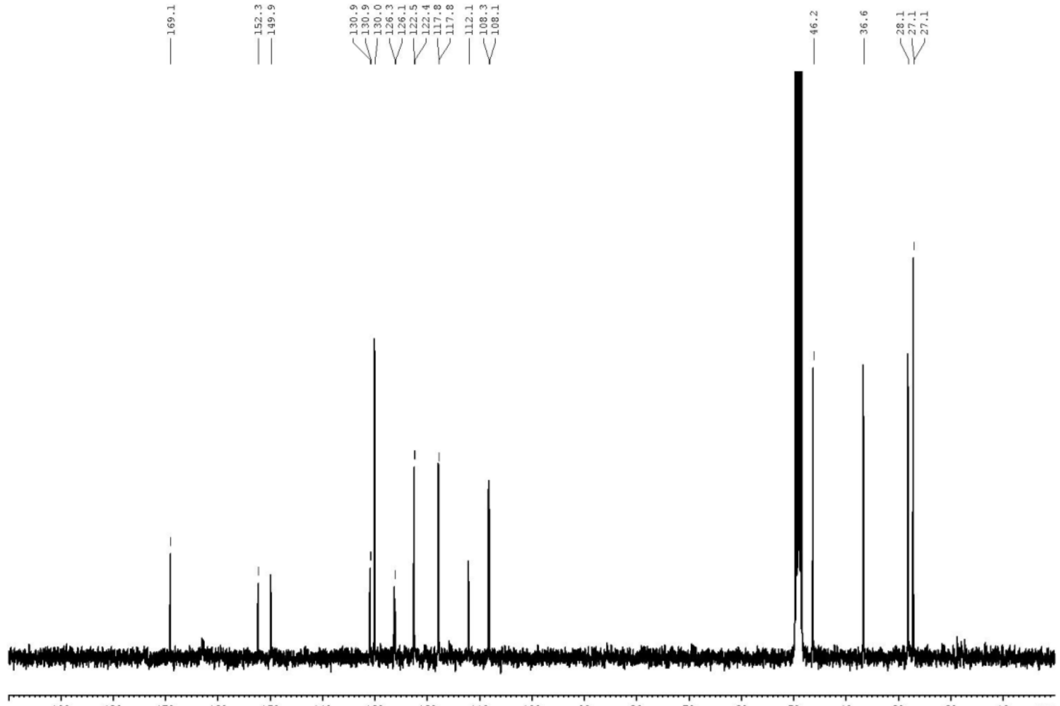

Figure S25 ^{1}H (CD3OD, 400 MHz) and ^{13}C (CD3OD, 100 MHz) NMR spectra for **16b**

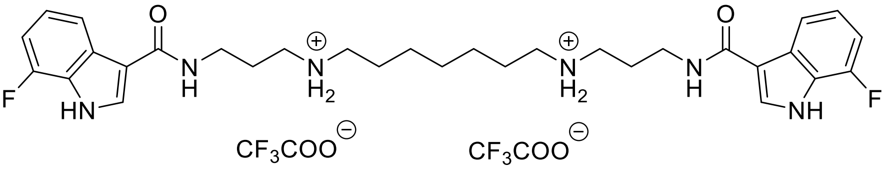

¹H NMR (CD₃OD, 400 MHz):

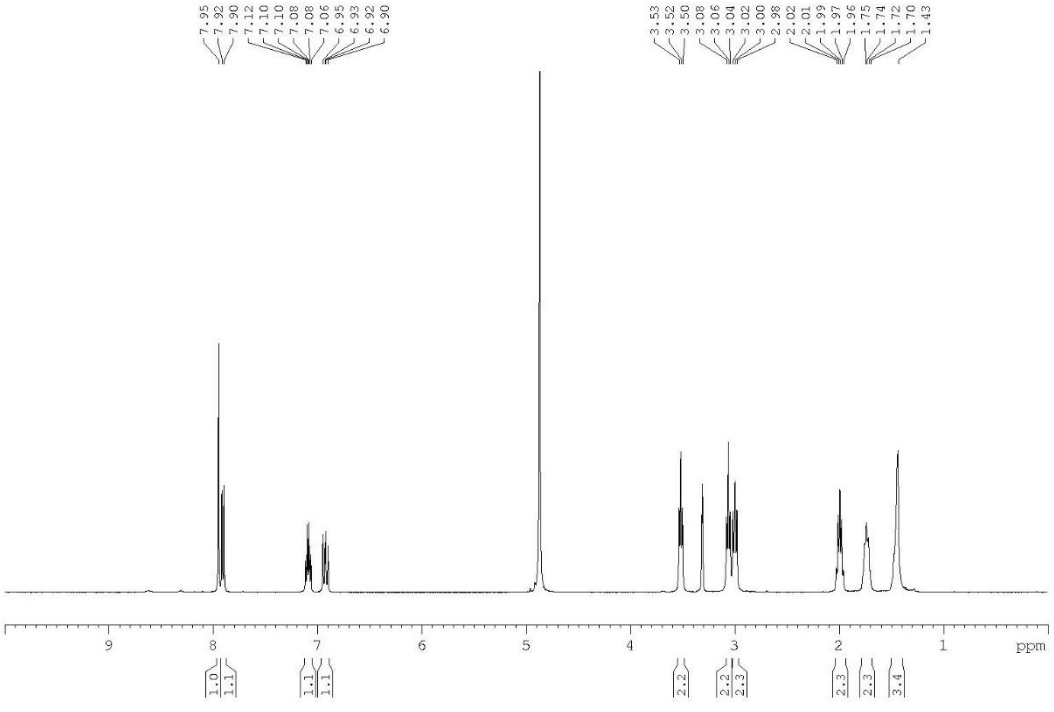

¹³C NMR (CD₃OD, 100 MHz):

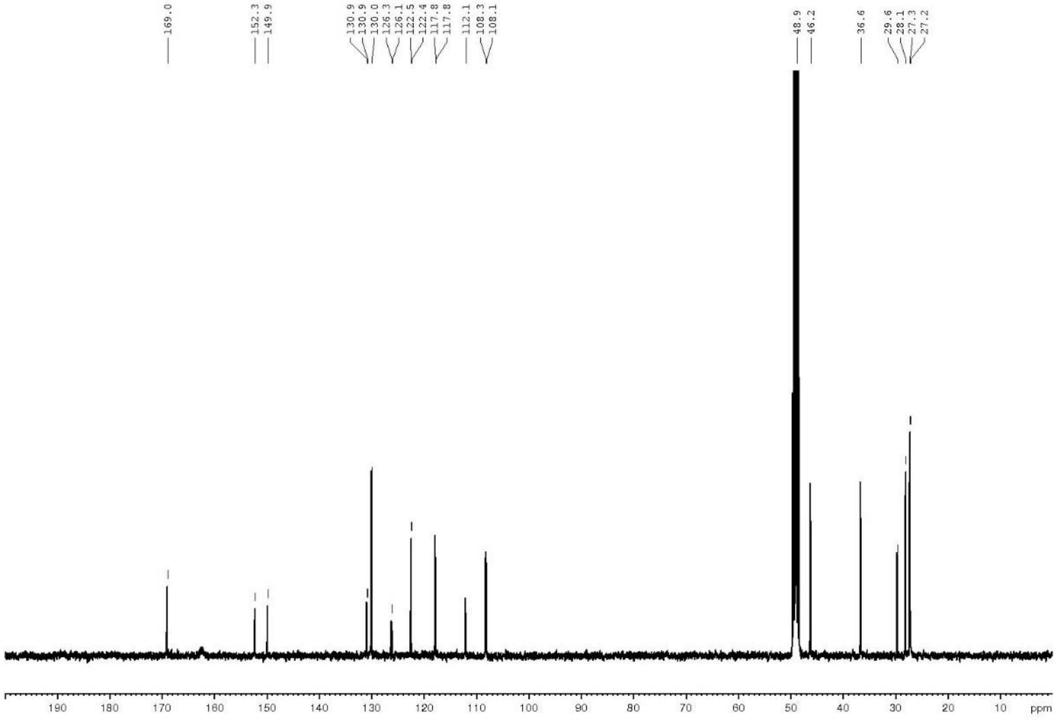

Figure S26 ¹H (CD₃OD, 400 MHz) and ¹³C (CD₃OD, 100 MHz) NMR spectra for **16c**

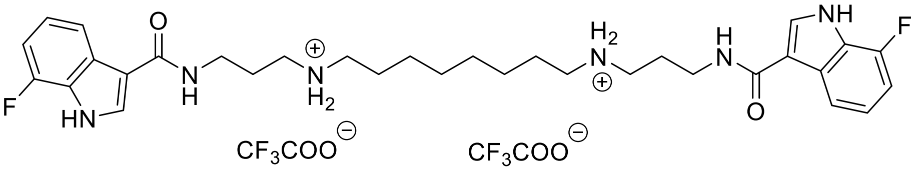

^{1}H NMR (CD_3OD, 400 MHz):

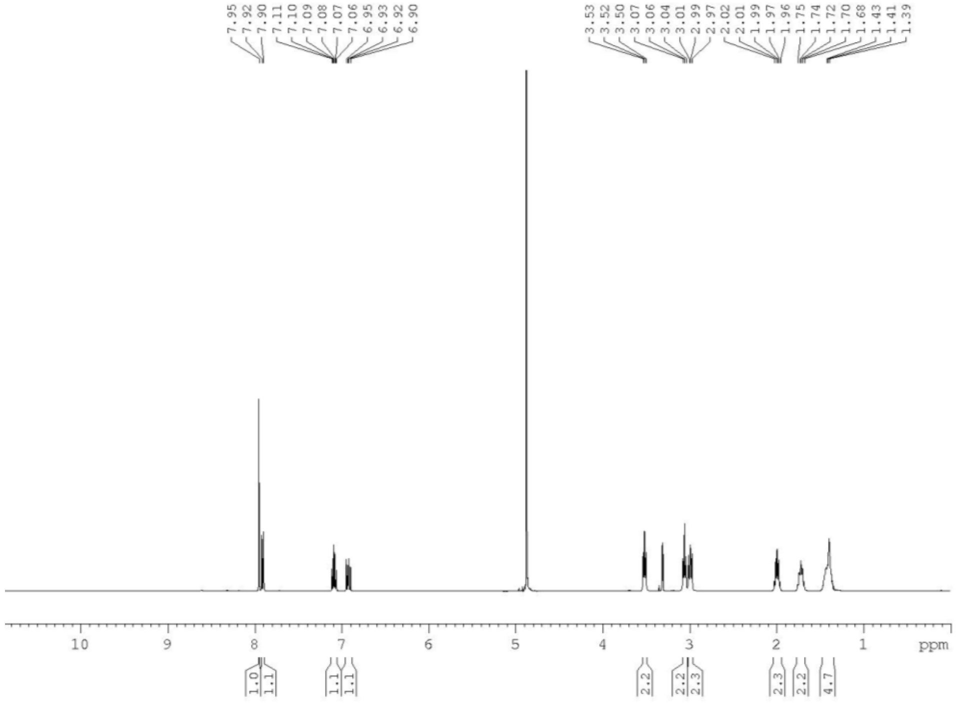

^{13}C NMR (CD_3OD, 100 MHz):

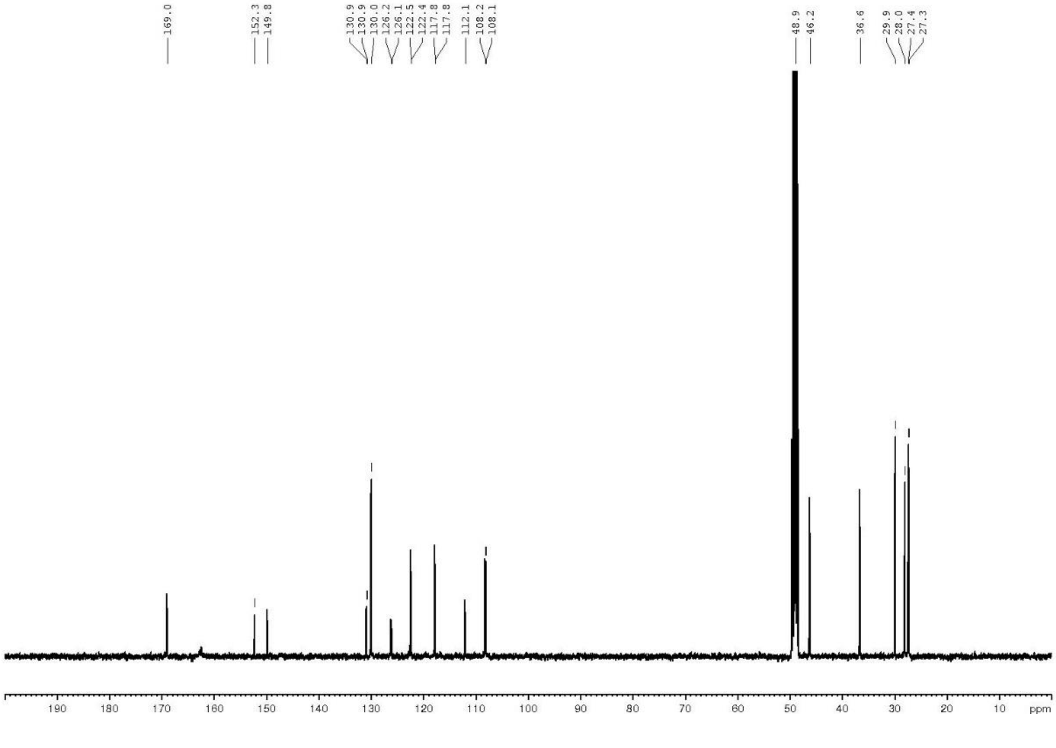

Figure S27 ^{1}H (CD_3OD, 400 MHz) and ^{13}C (CD_3OD, 100 MHz) NMR spectra for **16d**

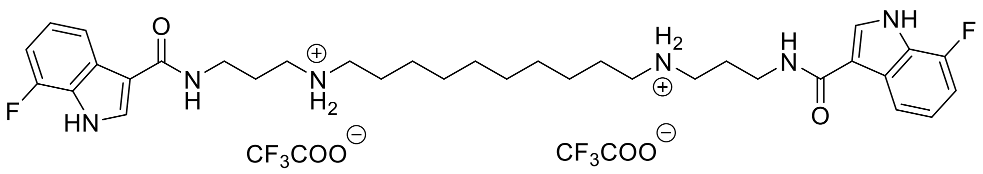

^{1}H NMR (CD_3OD, 400 MHz):

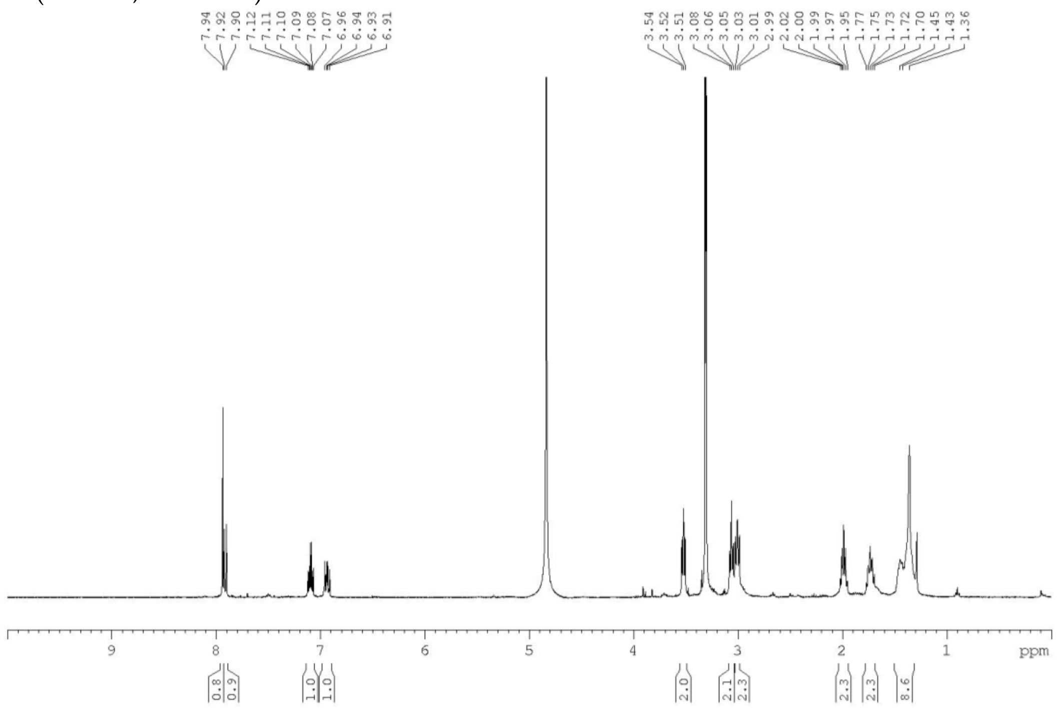

^{13}C NMR (CD_3OD, 100 MHz):

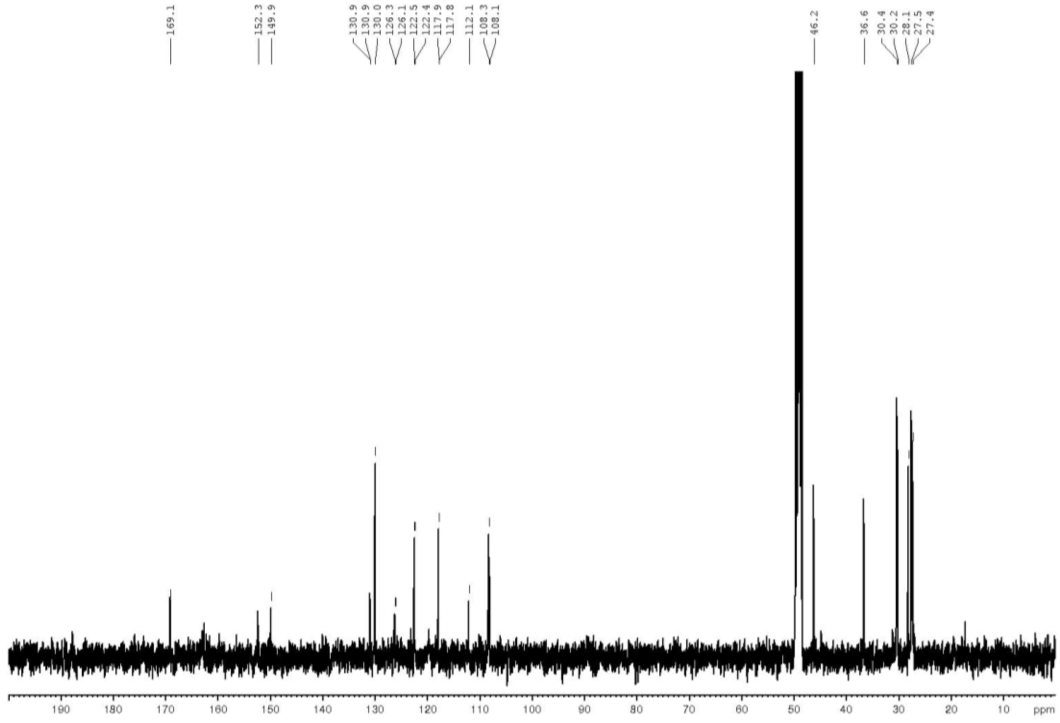

Figure S28 ^{1}H (CD_3OD, 400 MHz) and ^{13}C (CD_3OD, 100 MHz) NMR spectra for **16e**

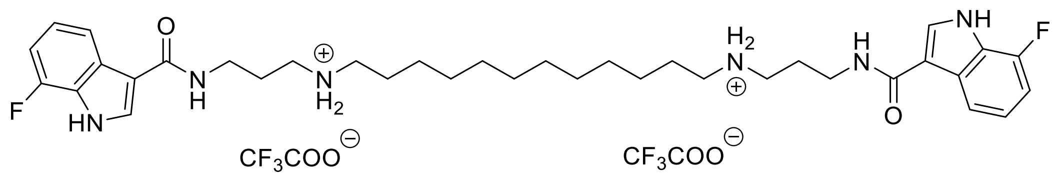

^{1}H NMR (CD_3OD, 400 MHz):

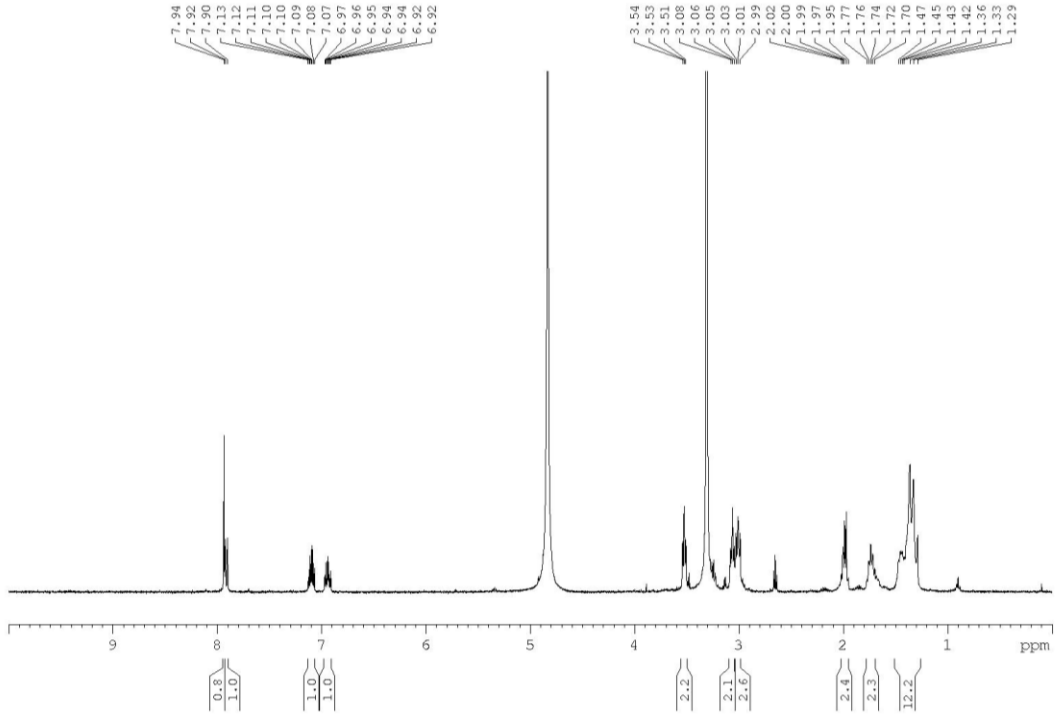

^{13}C NMR (CD_3OD, 100 MHz):

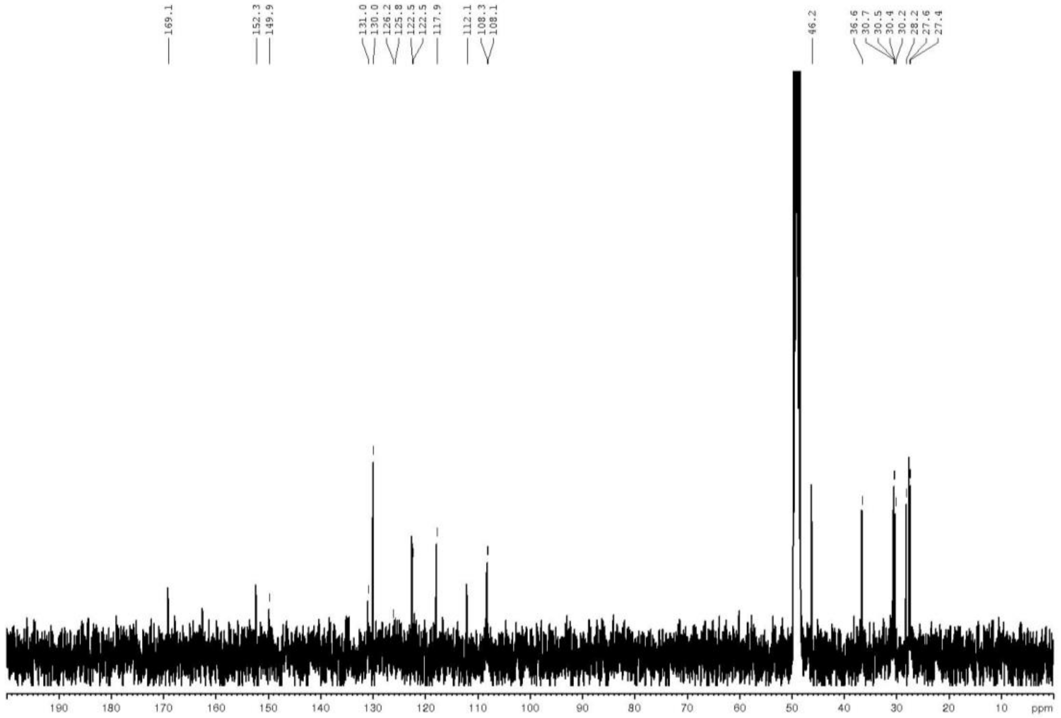

Figure S29 ^{1}H (CD_3OD, 400 MHz) and ^{13}C (CD_3OD, 100 MHz) NMR spectra for **16f**

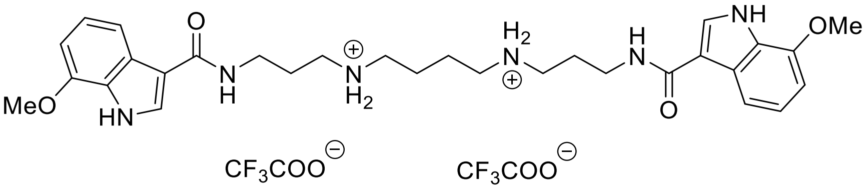

^{1}H NMR (CD_3OD, 400 MHz):

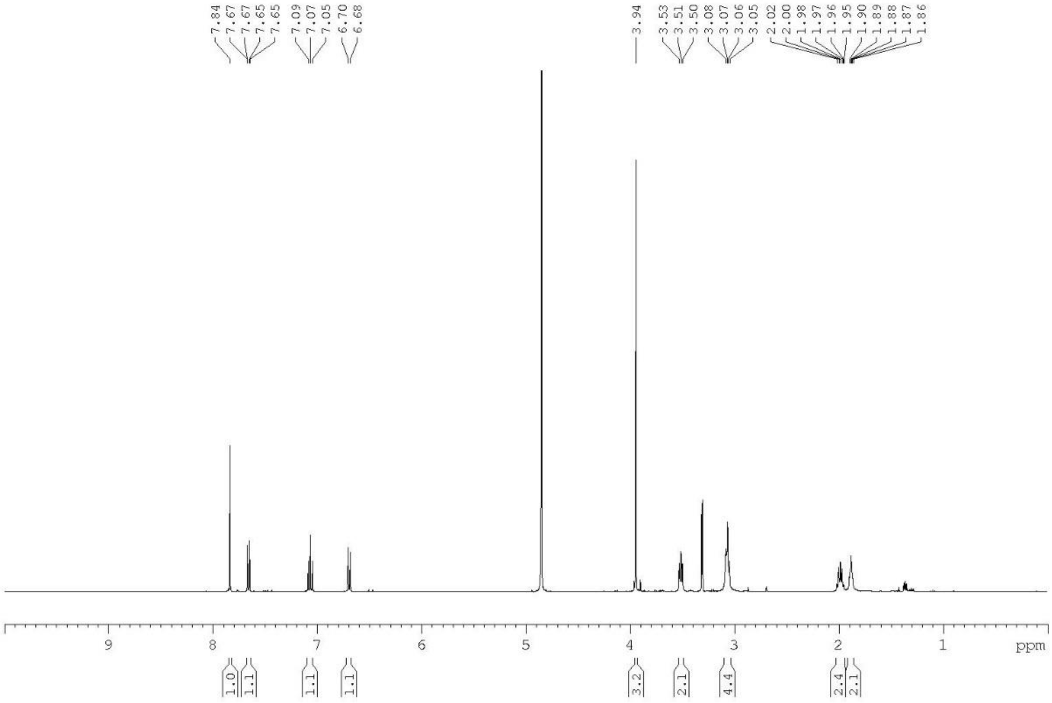

^{13}C NMR (CD_3OD, 100 MHz):

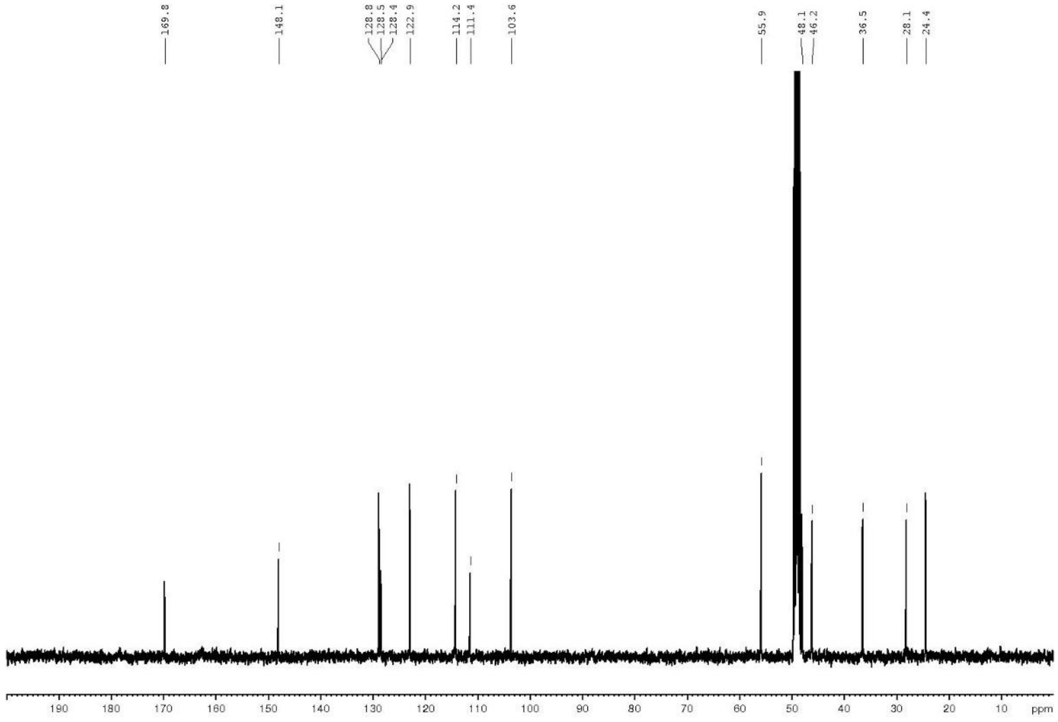

Figure S30 ^{1}H (CD_3OD, 400 MHz) and ^{13}C (CD_3OD, 100 MHz) NMR spectra for **17a**

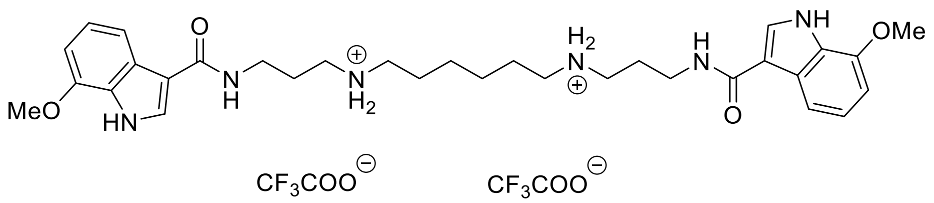

^{1}H NMR (CD_3OD, 400 MHz):

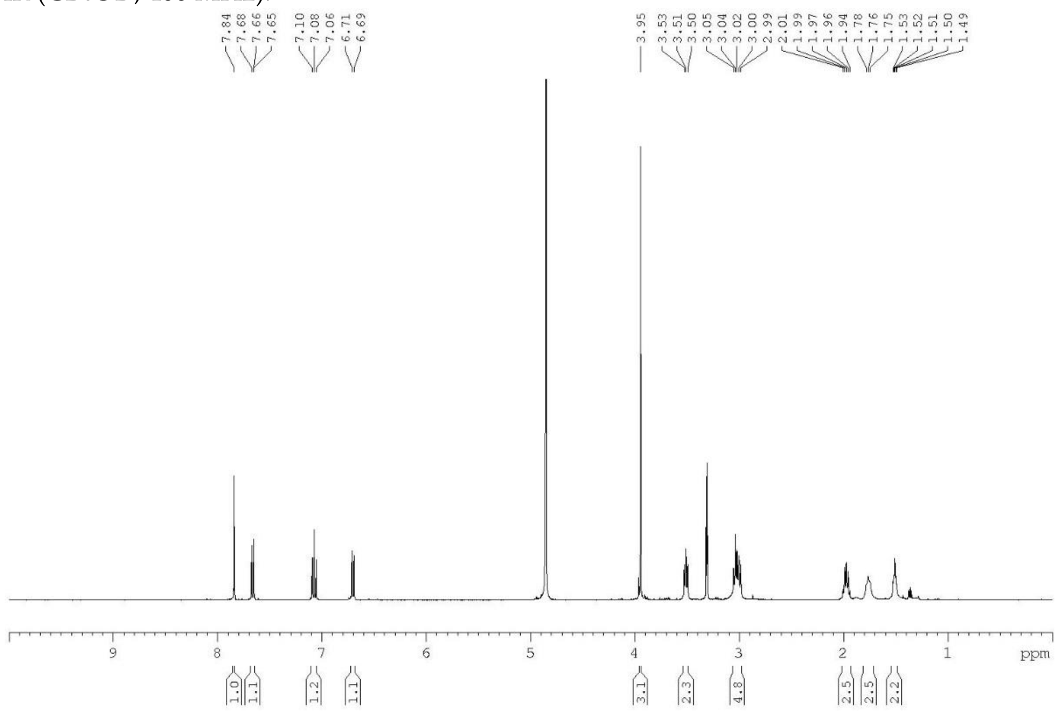

^{13}C NMR (CD_3OD, 100 MHz):

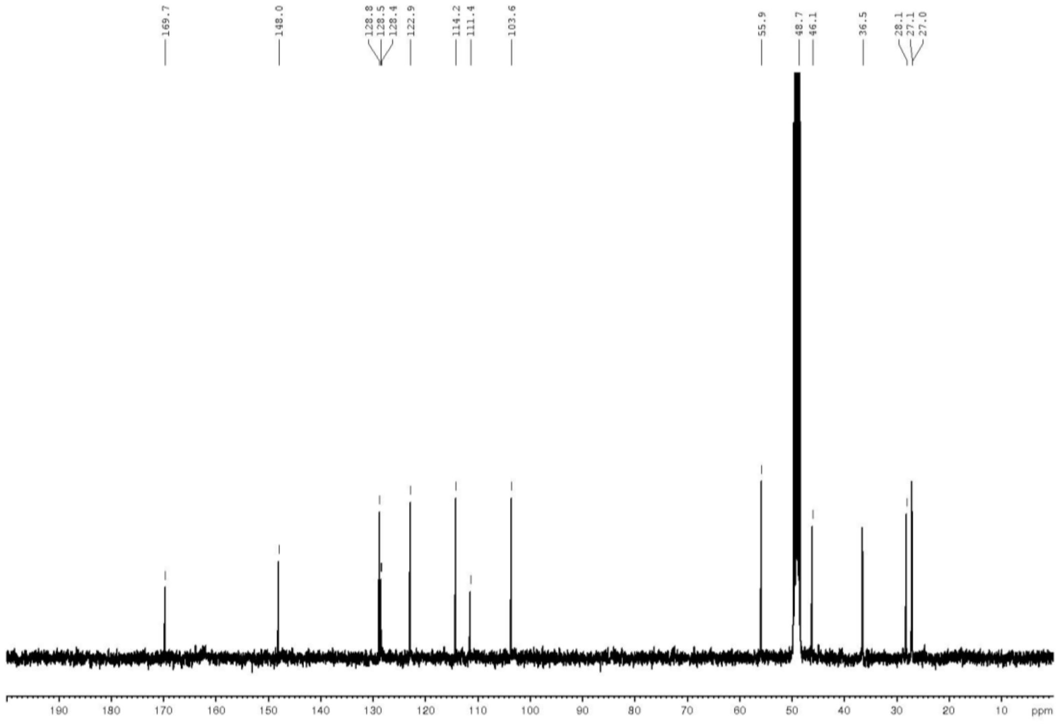

Figure S31 ^{1}H (CD_3OD, 400 MHz) and ^{13}C (CD_3OD, 100 MHz) NMR spectra for **17b**

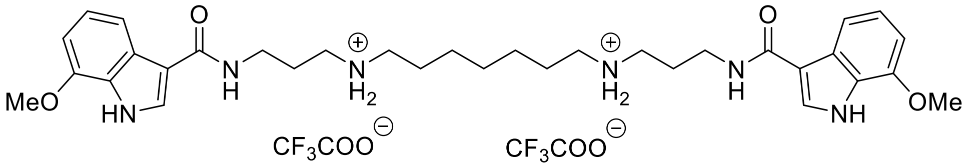

¹H NMR (CD₃OD, 400 MHz):

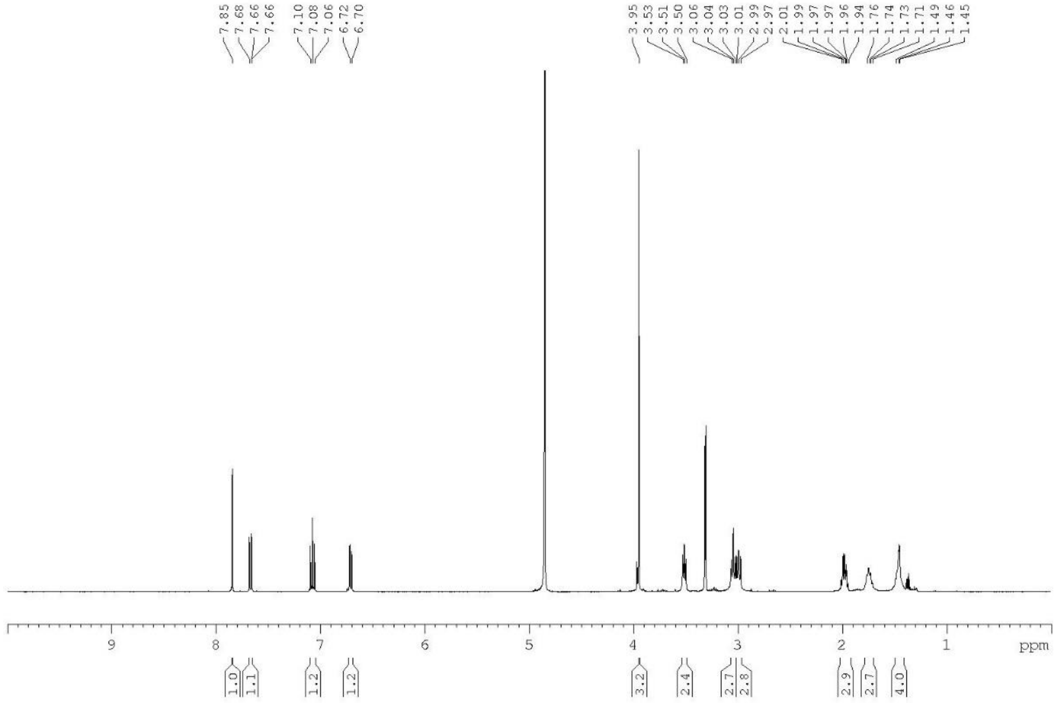

¹³C NMR (CD₃OD, 100 MHz):

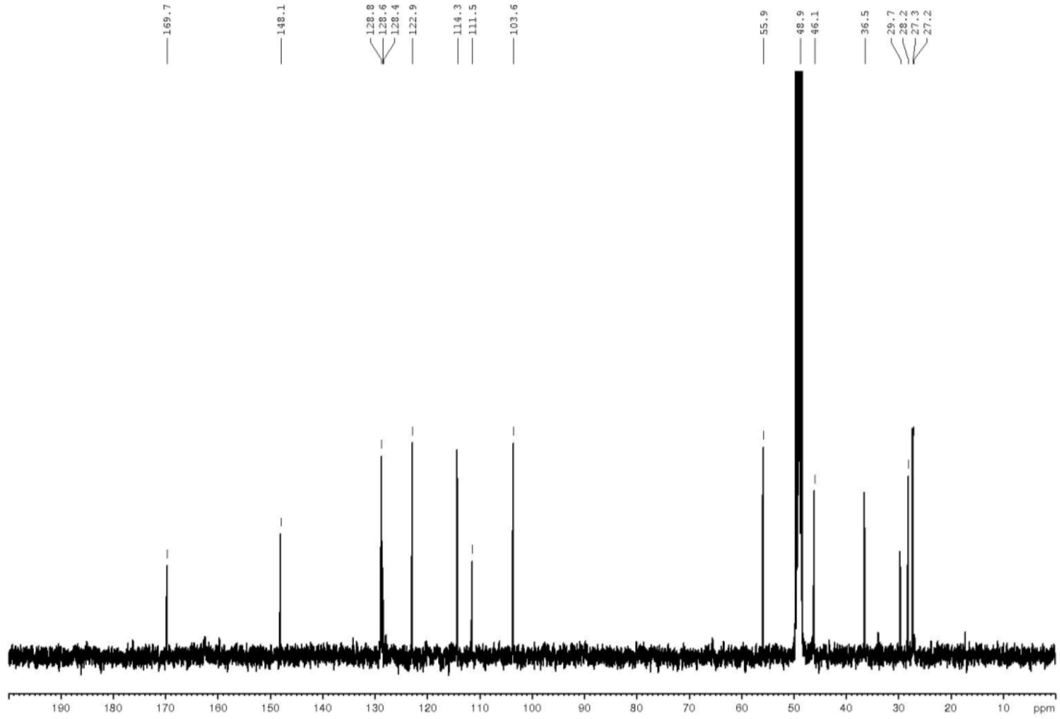

Figure S32 ¹H (CD₃OD, 400 MHz) and ¹³C (CD₃OD, 100 MHz) NMR spectra for **17c**

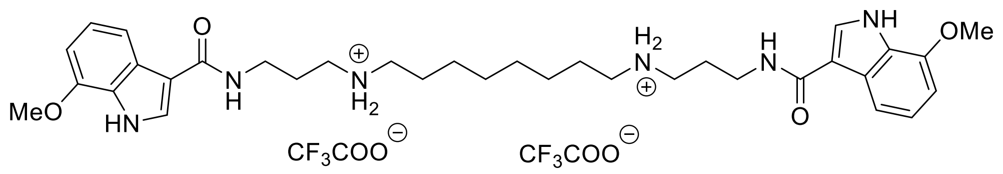

^{1}H NMR (CD_3OD, 400 MHz):

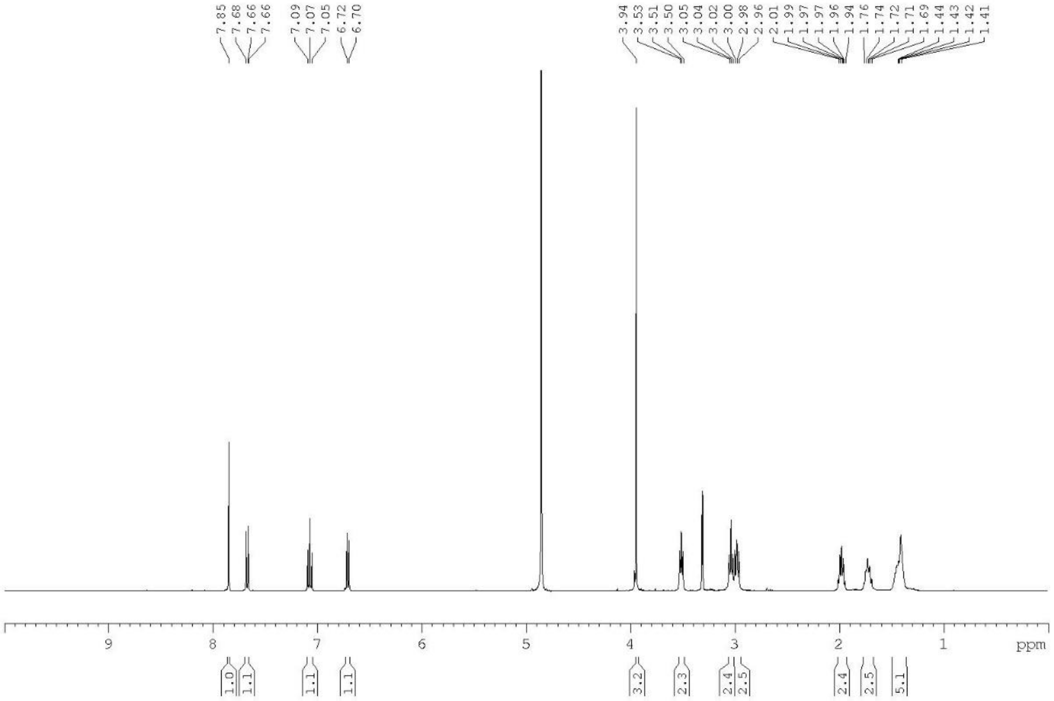

^{13}C NMR (CD_3OD, 100 MHz):

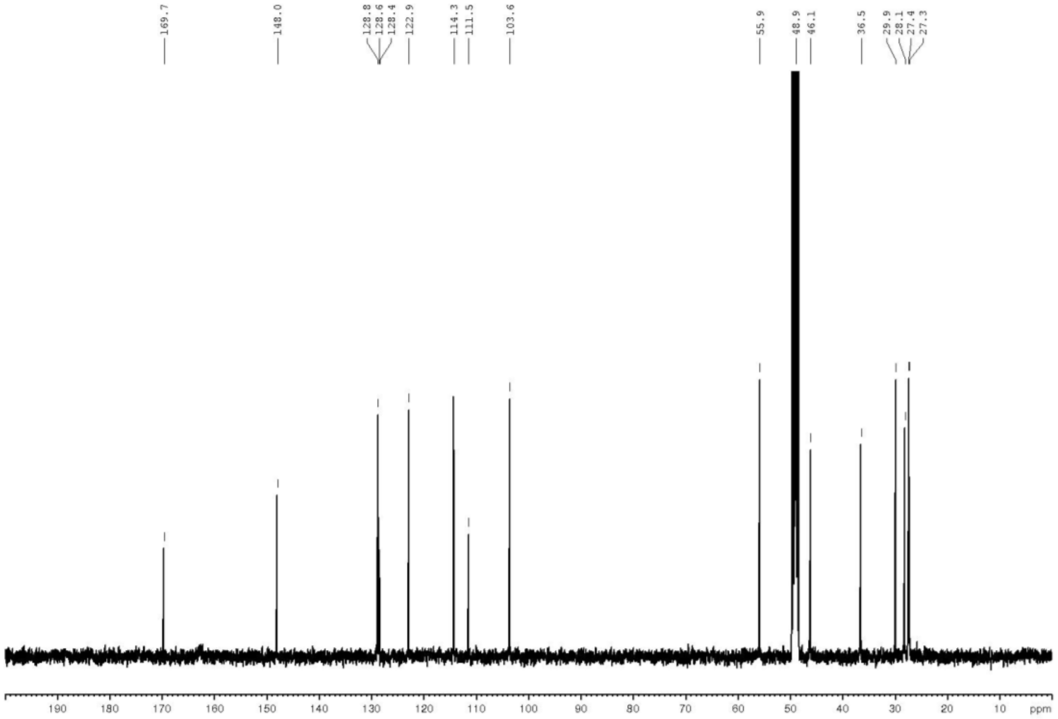

Figure S33 ^{1}H (CD_3OD, 400 MHz) and ^{13}C (CD_3OD, 100 MHz) NMR spectra for **17d**

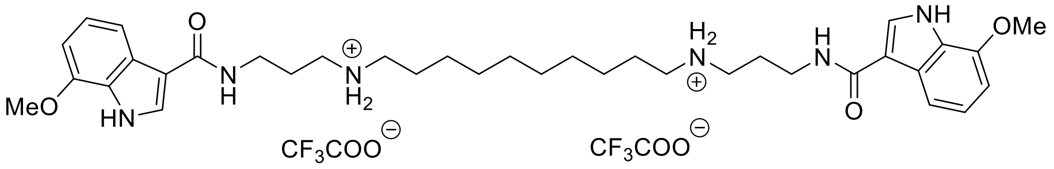

^{1}H NMR (CD_3OD, 400 MHz):

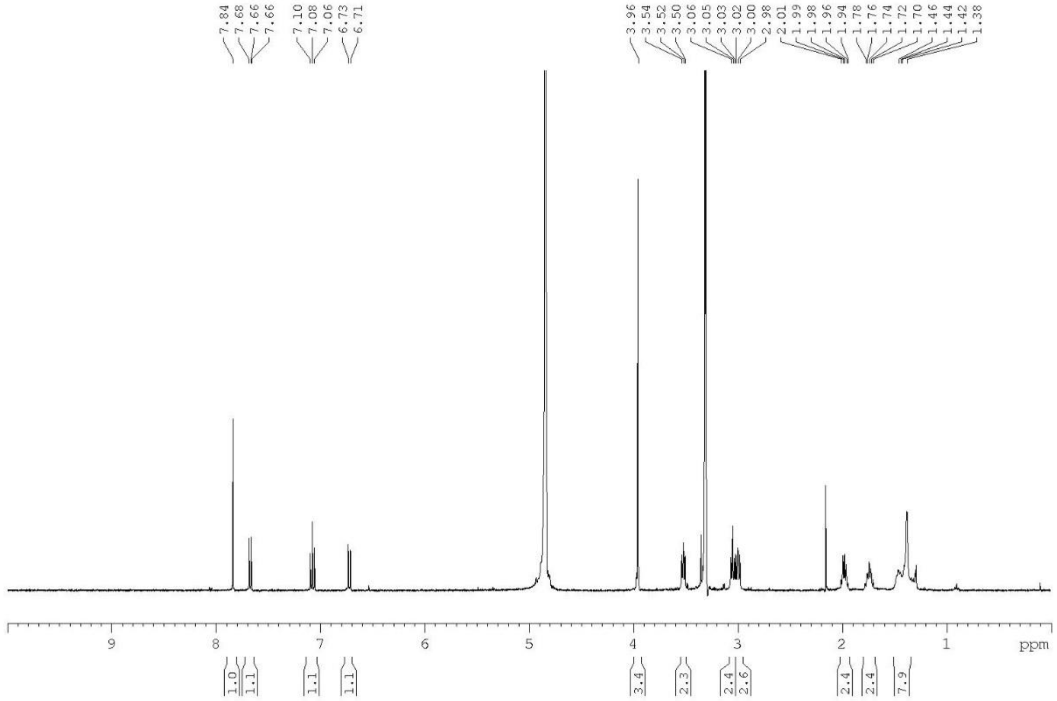

^{13}C NMR (CD_3OD, 100 MHz):

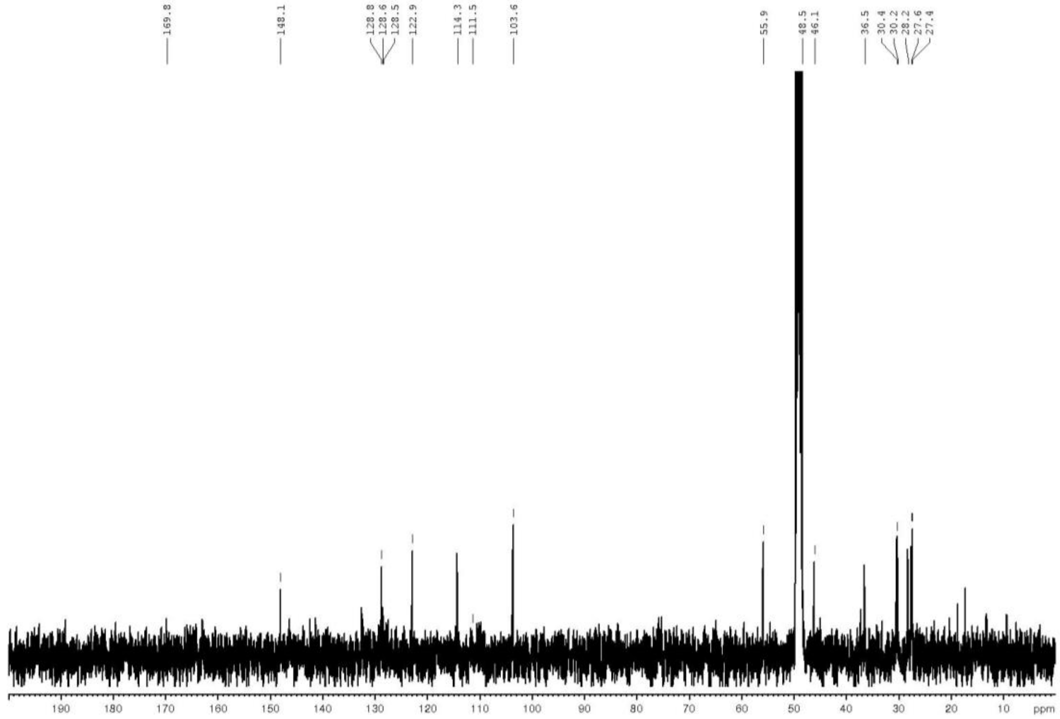

Figure S34 ^{1}H (CD_3OD, 400 MHz) and ^{13}C (CD_3OD, 100 MHz) NMR spectra for **17e**

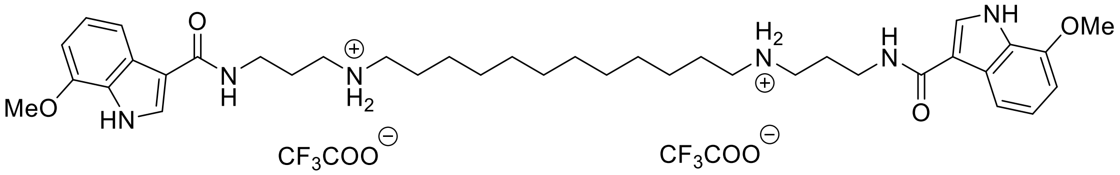

¹H NMR (CD₃OD, 400 MHz):

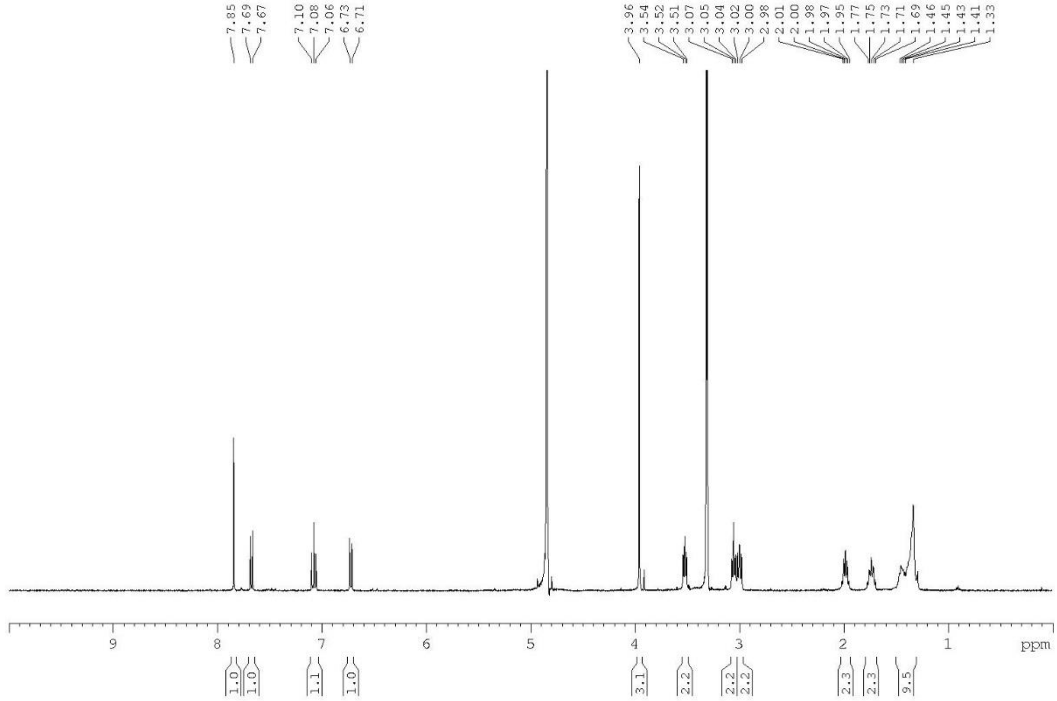

¹³C NMR (CD₃OD, 100 MHz):

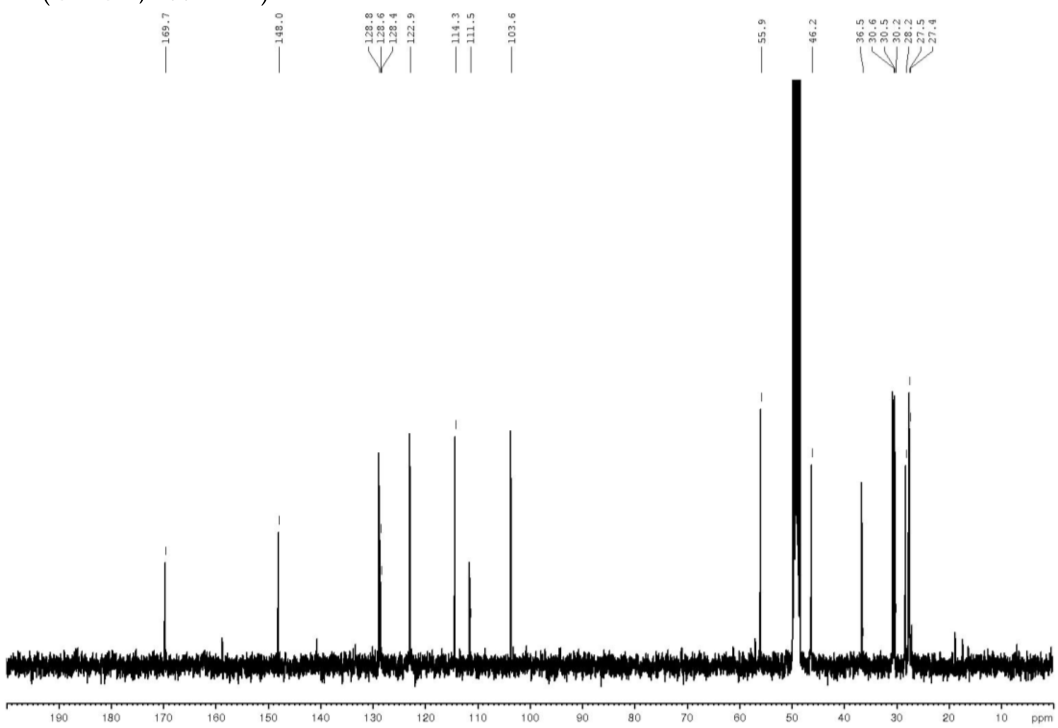

Figure S35 ¹H (CD_3OD, 400 MHz) and ¹³C (CD_3OD, 100 MHz) NMR spectra for **17f**

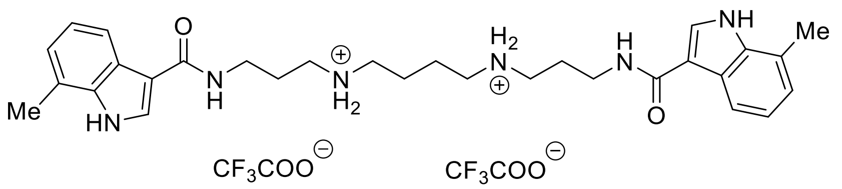

^{1}H NMR (CD_3OD, 400 MHz):

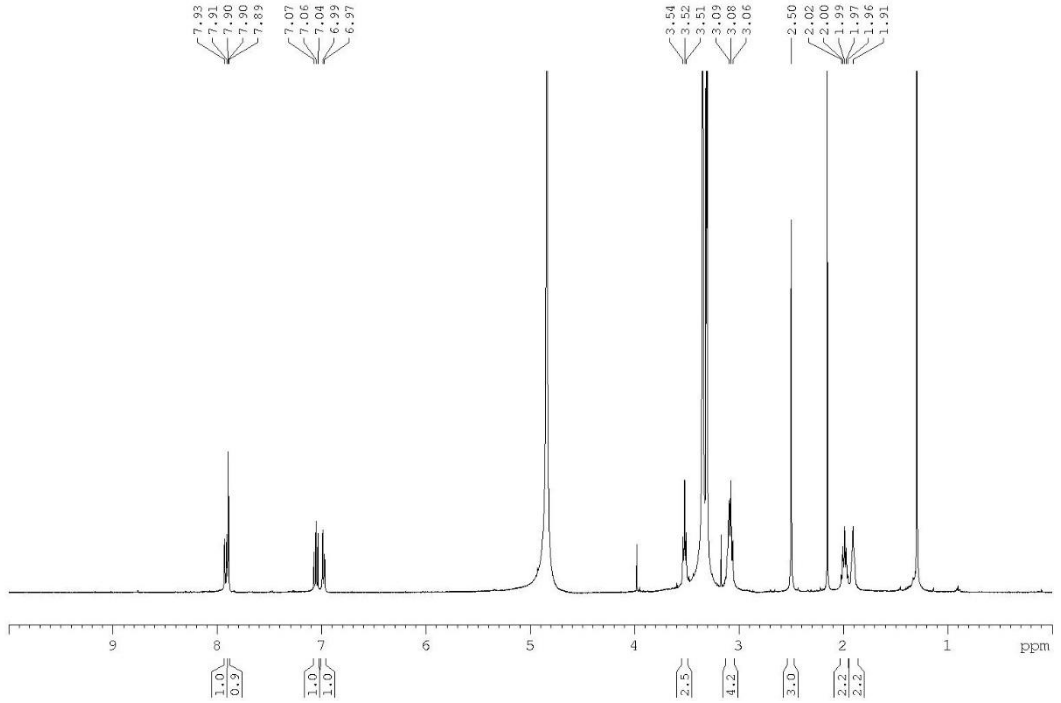

^{13}C NMR (CD_3OD, 100 MHz):

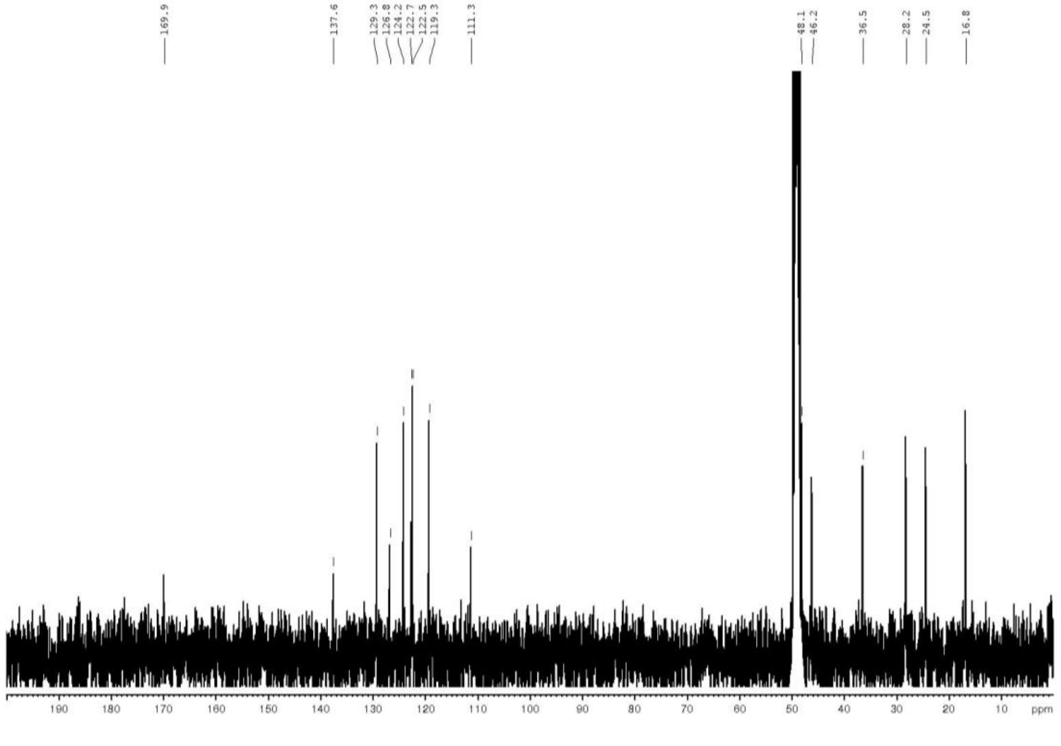

Figure S36 ^{1}H (CD_3OD, 400 MHz) and ^{13}C (CD_3OD, 100 MHz) NMR spectra for **18a**

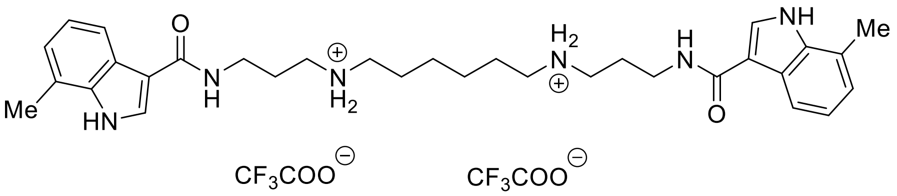

^{1}H NMR (CD_3OD, 400 MHz):

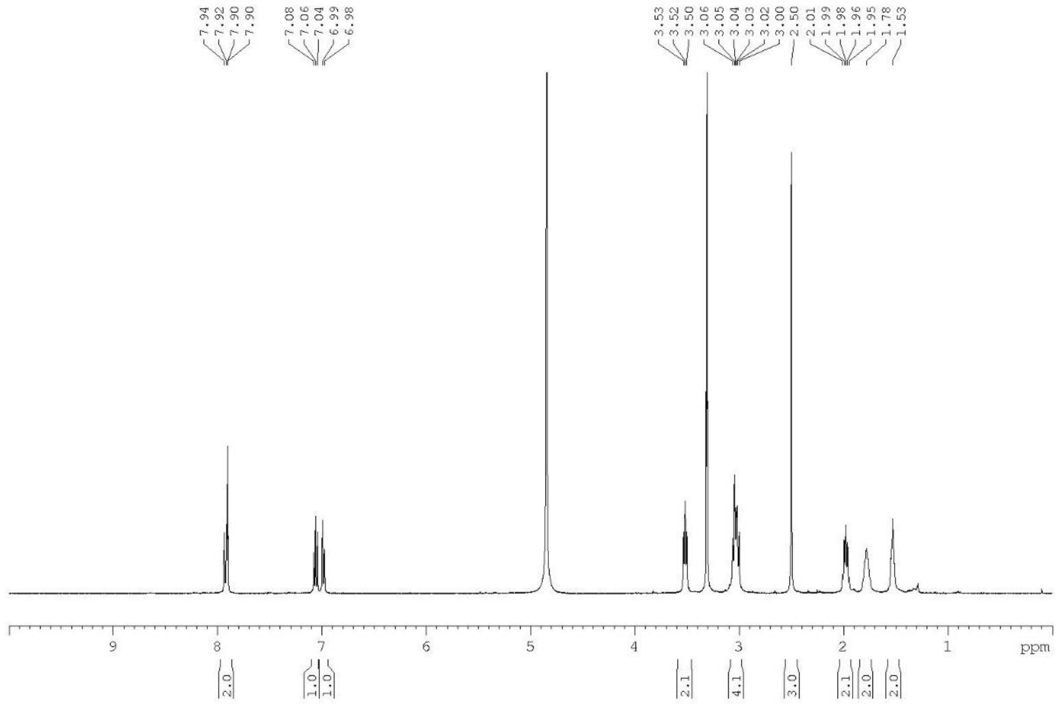

^{13}C NMR (CD_3OD, 100 MHz):

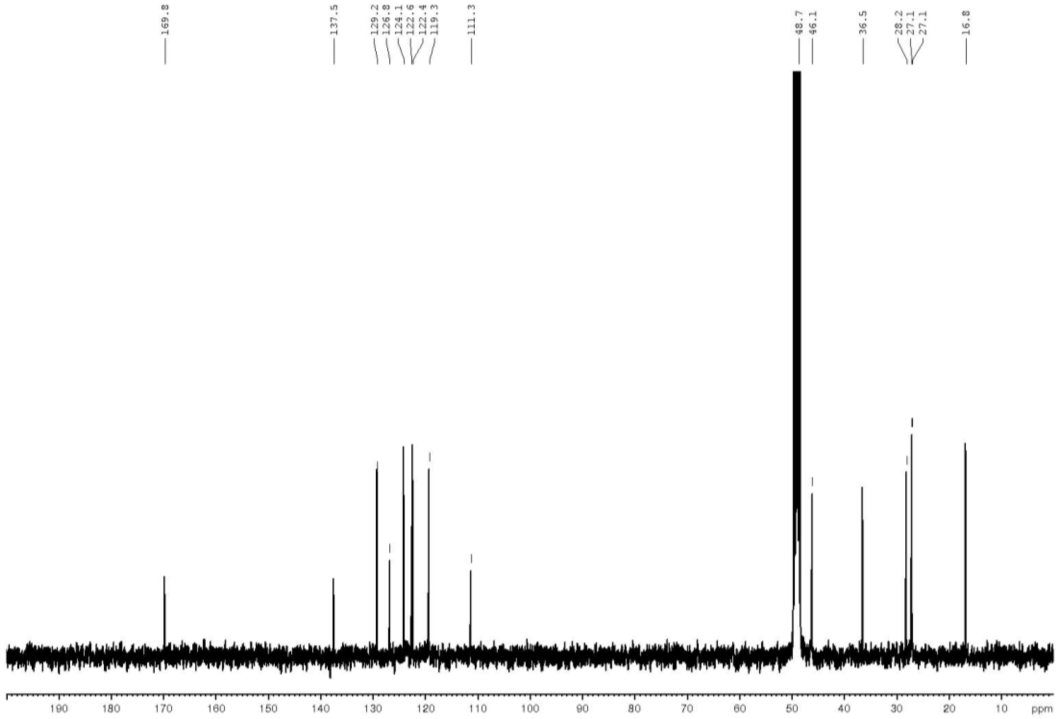

Figure S37 ^{1}H (CD_3OD, 400 MHz) and ^{13}C (CD_3OD, 100 MHz) NMR spectra for **18b**

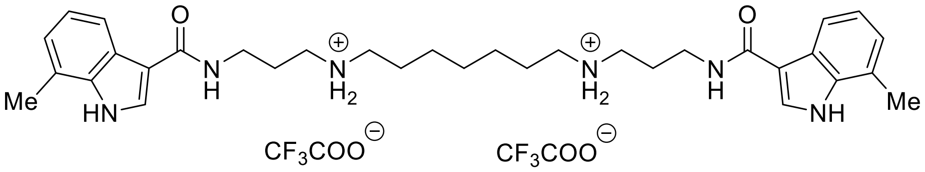

^{1}H NMR (CD_3OD, 400 MHz):

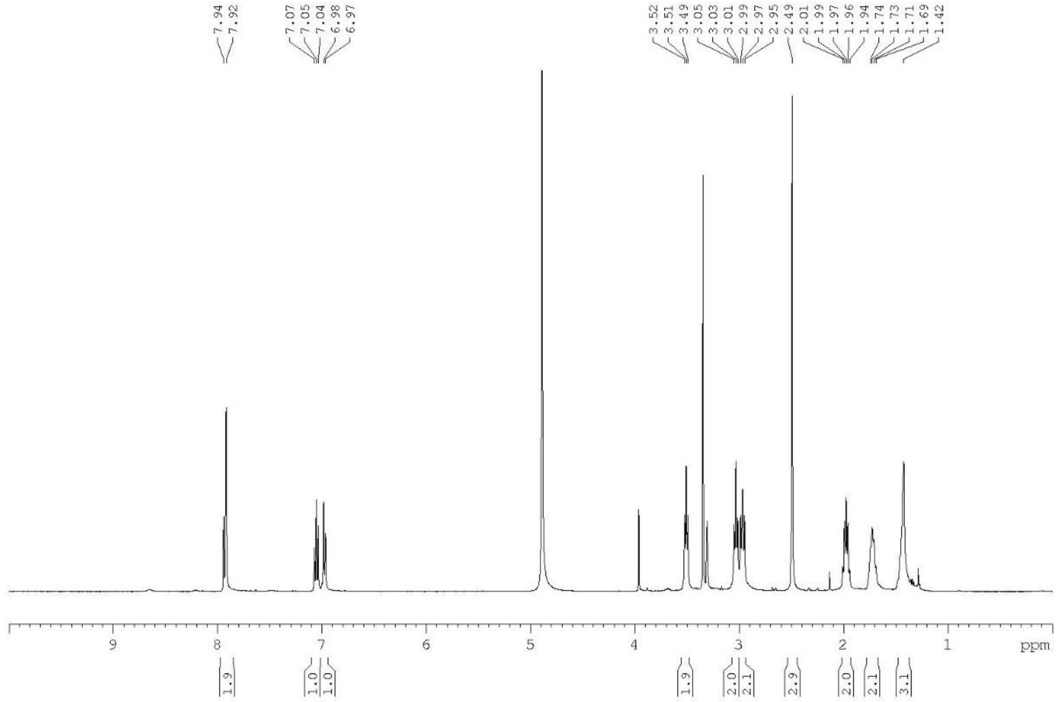

^{13}C NMR (CD_3OD, 100 MHz):

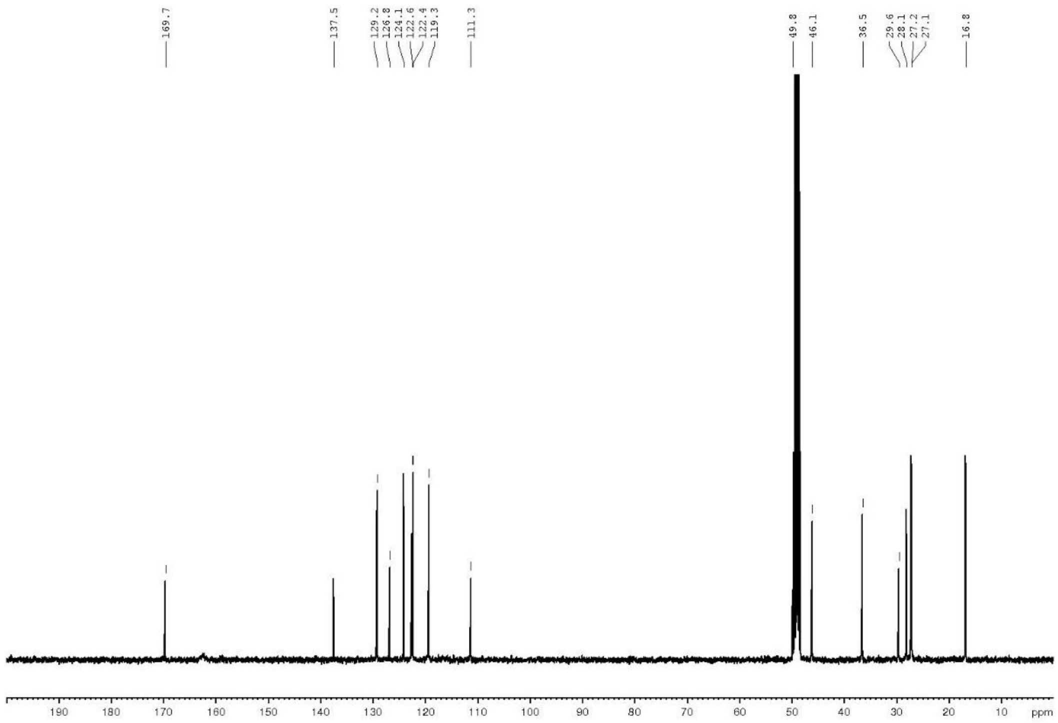

Figure S38 ^{1}H (CD_3OD, 400 MHz) and ^{13}C (CD_3OD, 100 MHz) NMR spectra for **18c**

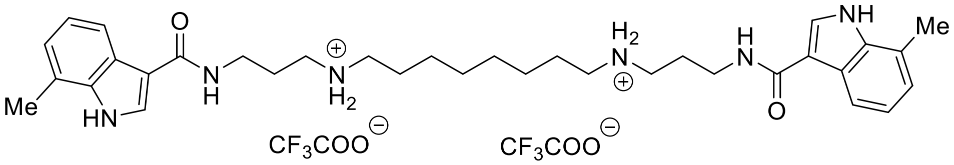

^{1}H NMR (CD_3OD, 400 MHz):

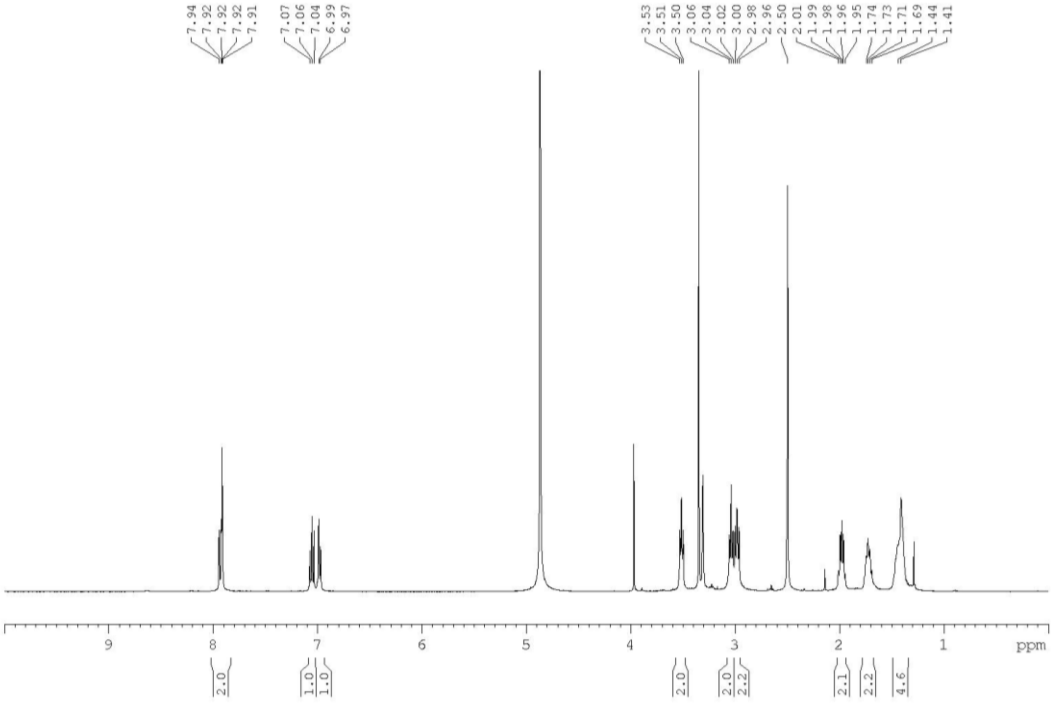

^{13}C NMR (CD_3OD, 100 MHz):

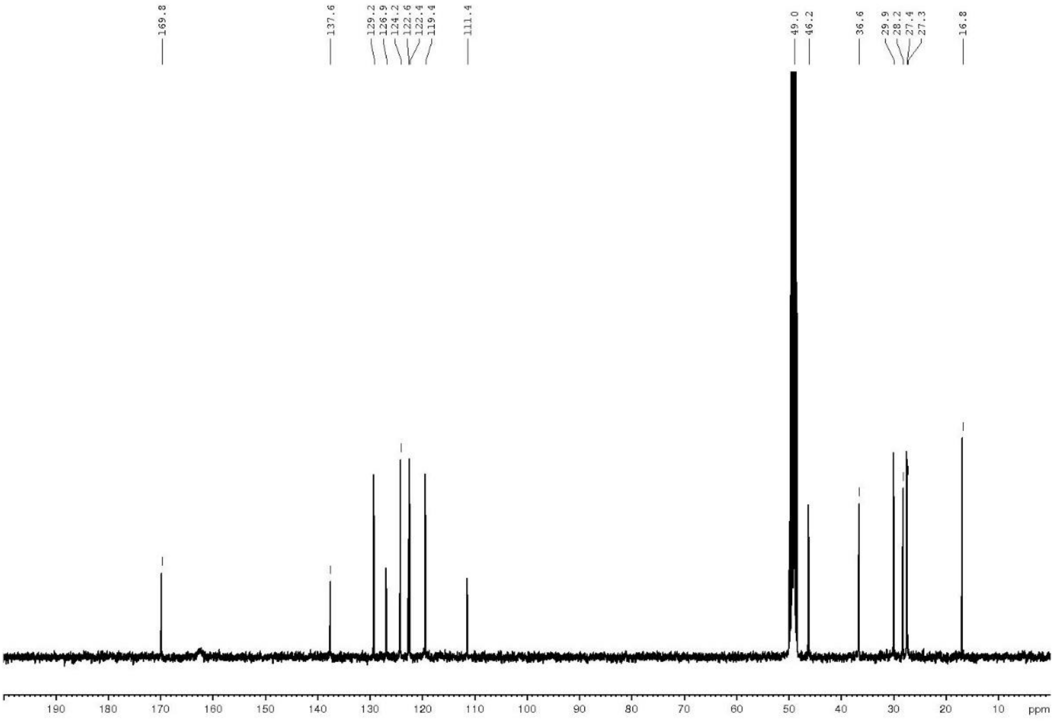

Figure S39 ^{1}H (CD_3OD, 400 MHz) and ^{13}C (CD_3OD, 100 MHz) NMR spectra for **18d**

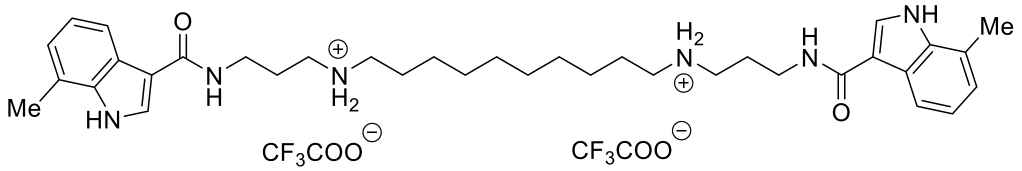

^{1}H NMR (CD_3OD, 400 MHz):

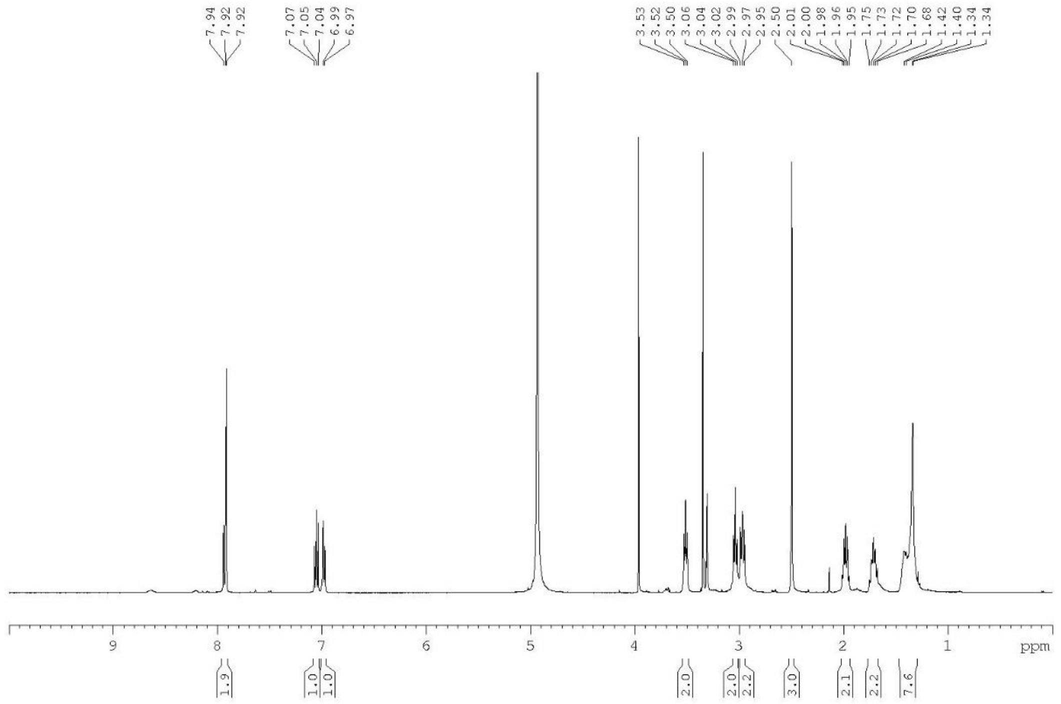

^{13}C NMR (CD_3OD, 100 MHz):

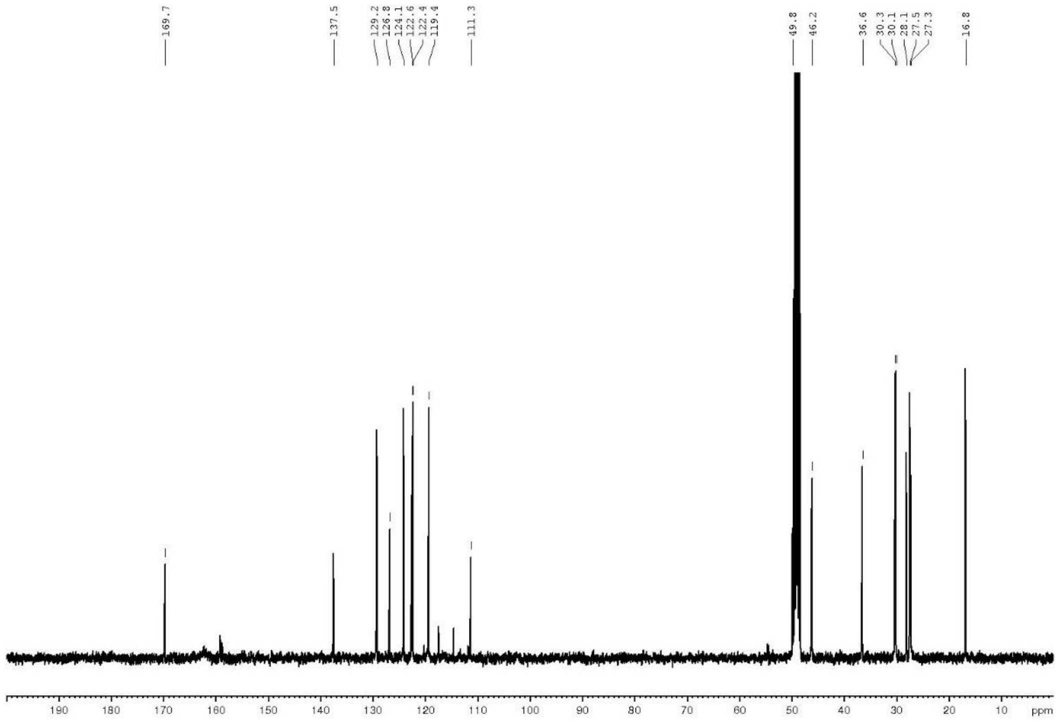

Figure S40 ^{1}H (CD_3OD, 400 MHz) and ^{13}C (CD_3OD, 100 MHz) NMR spectra for **18e**

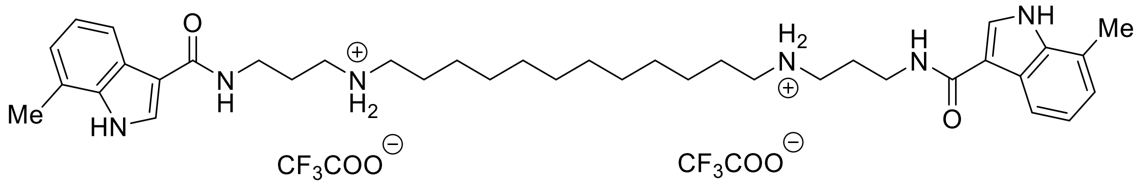

^{1}H NMR (CD_3OD, 400 MHz):

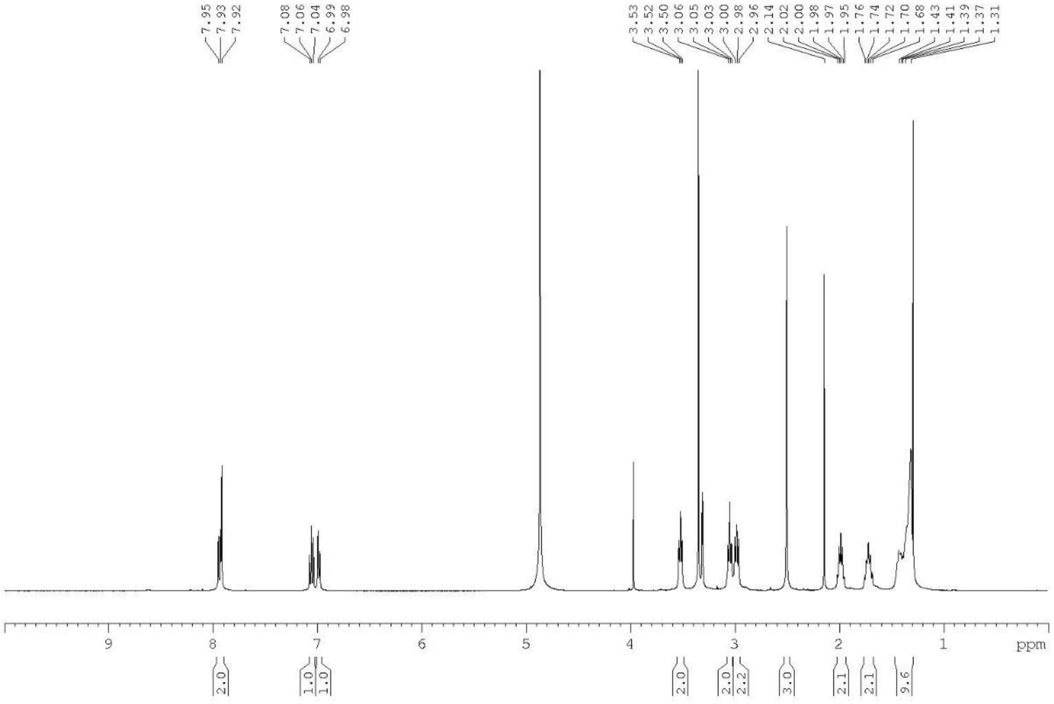

^{13}C NMR (CD_3OD, 100 MHz):

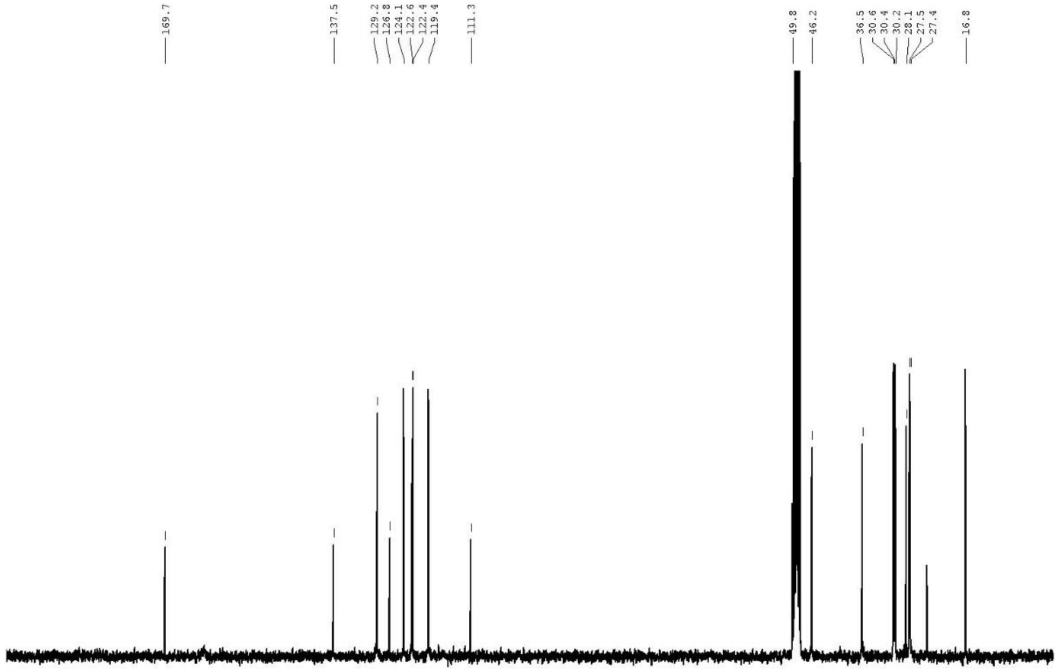

Figure S41 ^{1}H (CD_3OD, 400 MHz) and ^{13}C (CD_3OD, 100 MHz) NMR spectra for **18f**
